# The microbiome biomarkers of pregnant women’s vaginal area predict preterm prelabor rupture in Western China

**DOI:** 10.3389/fcimb.2024.1471027

**Published:** 2024-10-31

**Authors:** Yuanting Tang, Xia Wang, Jialing Huang, Yongmei Jiang, Fan Yu

**Affiliations:** ^1^ Department of Laboratory Medicine, West China Second University Hospital, Sichuan University, Chengdu, China; ^2^ Key Laboratory of Birth Defects and Related Diseases of Women and Children (Sichuan University), Ministry of Education, Chengdu, China; ^3^ Department of Clinical Laboratory, Sun Yat-sen Memorial Hospital, Sun Yat-sen University, Guangzhou, China

**Keywords:** vaginal opportunistic pathogens, PPROM, automatic analysis, microbiological tests, 16S rDNA sequencing technique

## Abstract

**Introduction:**

Intraamniotic infection is crucial in preterm prelabor rupture of membranes(PPROM), a clinical condition resulting from the invasion of vaginal opportunistic microbes into the amniotic cavity. Although previous studies have suggested potential associations between infection and PPROM, the role of vaginalopportunistic bacteria in PPROM has received limited attention.

**Methods:**

This study aimed to confirm the vaginal bacterial etiology of PPROM. We investigated vaginal microbiotas using automatic analysis of vaginal discharge, microbiological tests, and 16s rRNA genehigh-throughput sequencing.

**Results:**

The research findings revealed that the proportion of parabasal epitheliocytes, leukocytes, toxic leukocytes, and bacteria with diameters smaller than 1.5 um was significantly higher in the PPROM group than that in the normal full-term labor (TL) group. The top three vaginal opportunistic bacterial isolates in all participants were 9.47% Escherichia coli, 5.99% Streptococcus agalactiae, and 3.57% Enterococcus faecalis. The bacterial resistance differed, but all the isolates were sensitive to nitrofurantoin. Compared with the vaginal microbiota dysbiosis (VMD) TL (C) group, the VMD PPROM (P) group demonstrated more operational taxonomic units, a high richness of bacterial taxa, and a different beta-diversity index. Indicator species analysis revealed that Lactobacillus jensenii, Lactobacillus crispatus, and Veillonellaceae bacterium DNF00626 were strongly associated with the C group. Unlike the C group, the indicator bacteria in the P group were Enterococcus faecalis, Escherichia coli, and Streptococcus agalactiae.

**Discussion:**

These findings provide solidevidence that an abnormal vaginal microbiome is a very crucial risk factorclosely related to PPROM. There were no unique bacteria in the vaginalmicrobiota of the PPROM group; however, the relative abundance of bacteria inthe abnormal vaginal flora of PPROM pregnancies differed. Antibiotics should bereasonably selected based on drug sensitivity testing. The findings presented in this paper enhance our understanding of Streptococcus agalactiae, Enterococcus faecalis, and Escherichia coli vaginal bacterial etiology of PPROM in Western China.

## Introduction

Preterm prelabor rupture of membranes (PPROM) is the rupture of membranes before the onset of labor, precisely 37 weeks of gestation, occurring in approximately 2%–4% of pregnancies and representing 40%~50% of preterm births (Siegler et al., 2020; Ambalpady et al., 2022). It is associated with severe adverse pregnancy outcomes, including maternal factors such as chorioamnionitis, endometritis, puerperium infection, dystocia, placental abruption, placental retention, and postpartum hemorrhage. Meanwhile, neonatal risks include complications of prematurity, such as respiratory distress, neonatal infection, neonatal premature septicemia, pulmonary hyaline membrane disease, and bronchopulmonary dysplasia (Bouchghoul et al., 2019).

PPROM arises from various causes and mechanisms, such as genetic predisposition, behavioral factors, environmental factors, obstetric complications, and intraamniotic infection (Bonasoni et al., 2022). Among these, intraamniotic infection has been demonstrated to be commonly associated with PPROM (Yang and Wang, 2023). However, the effect of abnormal vaginal bacteria on PPROM remains unclear.

The vaginal flora of pregnant women is dominated by *Lactobacillus* species (Pramanick et al., 2021). When *Lactobacillus* species decrease opportunistic pathogens increase, vaginal microflora dysbiosis occurs (Ma et al., 2022), which can lead to several adverse pregnancy outcomes, including PROM, preterm birth, intrauterine growth restriction, intrauterine infections, chorioamnionitis, Low birth weight infants, miscarriages, and stillbirths (Kumar et al., 2022). Bacteria commonly found in intrauterine tissues in association with preterm labor (PTB) are of vaginal origin, and one of the most common routes is via the vaginal canal (Tian et al., 2023). It has been revealed that treatment for some vaginal infections can reduce the incidence of PTB (Lacroix et al., 2022). However, only two studies in a meta-analysis of 12 studies found an association between PPROM and vaginal microflora dysbiosis (Juliana et al., 2020). Further studies have revealed that treating bacterial vaginosis (BV) with oral or vaginal metronidazole or vaginal clindamycin before 28 weeks of gestation does not reduce the incidence of PTB (Rebouças et al., 2019). Accordingly, additional research is needed.

This study aimed to investigate the vaginal bacterial etiology of PPROM by exploring the association between the vaginal microbiome in PPROM and normal full-term labor (TL), antibiotic susceptibility testing of the major isolates, and vaginal microecological status.

## Materials and methods

The study was reviewed and approved by the ethics committee of West China Second University Hospital of Sichuan University (Medical Research 2020 NO. 050 and 2023 NO. 131).

### Study design and patients

Singleton pregnant women were eligible if their gestational age was >14 weeks based on the last menstrual period or obstetrical estimation and were planning to receive ongoing prenatal and delivery in the Obstetric and Gynecological Department of West China Second University Hospital from January 2020 to December 2023. Written informed consent forms were obtained from all participants.

**Figure 1:**
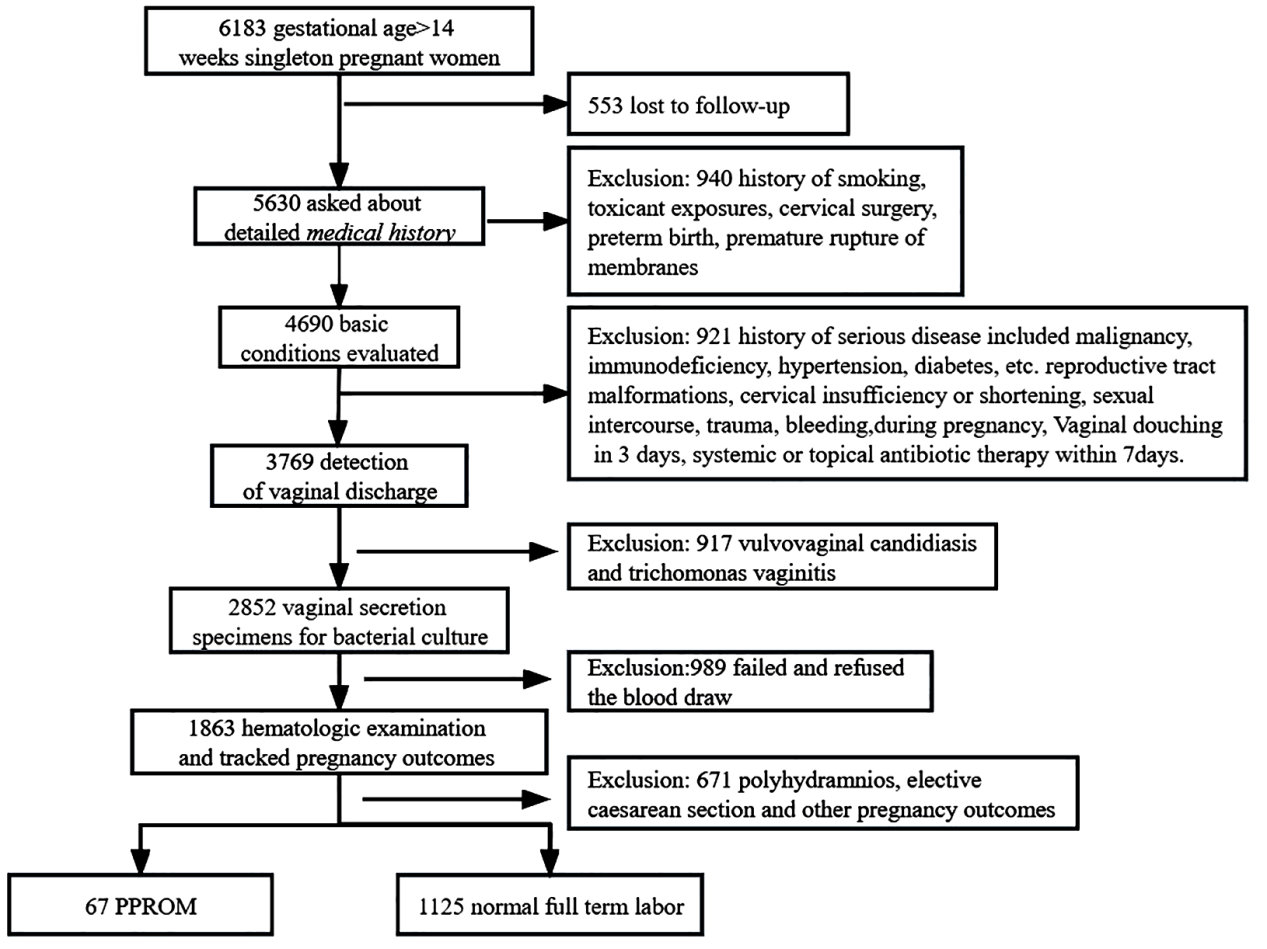
Flowchart for study subject inclusion and exclusion.

The exclusion criteria were as follows: (a) history of substance abuse, smoking, or toxic chemical exposure; contractions; preterm birth; cervical dilatation; or premature rupture of membranes; (b) severe medical diseases, such as hypertension, diabetes mellitus, malignant tumors, and abnormal immune function; (c) presence of specific pathogens, including fungal, trichomonad, viral, mycoplasma, and chlamydia, in the female reproductive system; (d) use of antibiotics within 7 days or inability to follow up, such as due to psychiatric illness; (e) vaginal bleeding, abdomen injury, and sexual intercourse during pregnancy. The inclusion and exclusion criteria for the study subject are detailed in the flowchart (**Figure 1**).

### Vaginal discharge collection

We collected vaginal secretions during routine prenatal check-ups for pregnant women beyond 14 gestational weeks. The time interval between the collection of vaginal secretions and the gestational week of delivery ranges from 2 to 20 days. We collected vaginal discharge that was taken from the posterior fornix or vaginal wall, via three sterile nylon-flocked swabs (Mairuikelin Technology Co. Science and Technology Ltd., Shenzhen, China). All sterile nylon-flocked swabs were put into sterile tubes (Di RuiI Industrial Company, Changchun, China) immediately and sent to the Department of Laboratory Medicine, West China Second University Hospital, Sichuan University. One swab was used for automatic analysis, another was analyzed for microbiological tests, and the last one was stored at −80°C immediately for subsequent deoxyribonucleic (DNA) extraction.

### Automatic analysis of vaginal discharge images

Automatic analysis of vaginal discharge images was performed using an automatic vaginal infections analyzer GMD-S600 (Di Rui Industrial Company, Changchun, China) with plane laminar liquid flow technology, high-speed camera technology, and artificial intelligent recognition technology. We upgraded a novel software analyzer of bacterial morphotype grading; calculated the average gray value, geometrical shape, and bacterial color (Ye et al., 2024); and input the characteristic value into an efficient support vector machine and back-propagation classifier to finish the recognition and classification. The new parameters and settings used in this study provided data on the long diameter of each bacterium, toxic leukocytes, and parabasal epitheliocytes (PBCs).

### Microbiological tests

Vaginal discharge samples were inoculated within 2 h of sampling. The swabs were streaked aerobically on Columbia sheep blood agar (Mérieux Shanghai Science and Technology Development Limited Company, Shanghai, China) and MacConkey plates (Antu Bioengineering Institute Co., Ltd., Zhengzhou, China) for 24–48 h at 35°C. Anaerobic culture was performed on fastidious anaerobic agar plates (Kailin Trading Co., Ltd., Jiangmen, China) for 7 d days at 35°C.

One colony was picked from the total bacterial population after confirmation through Gram staining; then, the strain identification was performed using matrix-assisted laser desorption/ionization time-of-flight mass spectrometry. Assays were repeated twice per strain; *Escherichia coli* ATCC 25922 was used as the quality control strain. Following the manufacturer instructions, the antimicrobial susceptibility testing (AST) of *Enterobacteriaceae, Pseudomonas*, and *Acinetobacter* isolates was determined using the Gram-negative susceptibility card AST-GN13/16; *Staphylococcaceae* and *Enterococcus* isolates were tested using the AST-GP67 (all bioMérieux); *Streptococcus* isolates were tested using the TDR STR-AST (Tian Diren *Streptococcus* antimicrobial susceptibility testing) detection kit (Tian Diren, China).

### 16s rRNA gene high-throughput sequencing

Pregnant women with VMD underwent 16S rRNA gene amplification and sequencing. The DNA was extracted from clinical vaginal swabs using the FastDNA^®^ SPIN Kit for Soil (MP Biomedicals, USA). The 16S polymerase chain reaction amplification targeted the V3–V4 hypervariable region with Forward Primer (5′–3′): 341CCTACGGGNGGCWGCAG and Reverse Primer 805 (5′–3′): GACTACHVGGGTATCTAATCC (Chengdu Qingke Technology, Co., China). Libraries were multiplexed and sequenced on an Illumina MiSeq platform (MiSeq Reagent Kit v3, USA) at 2 × 250 bp. Finally, the sequencing data were bioinformatically analyzed. Microbial diversity was analyzed using usearch, Mothur, QIIME1, and QIIME2 and displayed with Mothur and R version 3.5.3 software (Aberhe et al., 2020; Trifi et al., 2020; Kizilirmak et al., 2021). The Linear discriminant analysis effect size analysis (LEfSe) is performed to determine the features that most likely to explain differences between groups. A |LDA|>2 and a P<0.05 are used for screening.

### Data analysis

Statistical analyses and construction of statistical charts were performed using GraphPad Software version 8.0 (GraphPad Software Inc., CA, USA). The normal distribution of data is expressed as mean ± standard deviation, assessed using the t-test. However, non-normally distributed data are expressed as median (interquartile range) applied using the Mann–Whitney U test. Constituent ratios were assessed using the chi-square or Fisher’s exact probability tests.

## Results

### Basic information and clinical features

A total of 3,769 vaginal discharge samples from 4,690 pregnant women were included in this study. A total of 1,863 cases were tracked for pregnancy outcomes; 3.6% (67/1863) of cases displayed PPROM, and 60.39% (1,125/1,863) controls demonstrated normal full-term labor (TL). A total of 671 participants were excluded due to vaginal bleeding, trauma, sexual intercourse during pregnancy, polyhydramnios, abortion, preterm delivery, placental abruption, placenta praevia, term premature rupture of membranes, and elective cesarean.

The PPROM group aged 19–44, and the TL group aged 17–49. The two groups demonstrated non-significant differences in age (*P* > 0.05). Analysis of the prepregnancy body mass index (BMI) revealed that 56.72% (38/67) were overweight (BMI of 25–29.9) in the PPROM group, whereas 30.04% (338/1,125) were overweight in the TL group. The two groups did not differ statistically, in the rate of cesarean sections. The high incidence of clinical chorioamnionitis and neonatal asphyxia in the PPROM group weighed less than in the TL group (**Table 1**).

**Table 1:** Baseline demographics and clinical characteristics were compared between the PPROM group and the TL group.

	PPROM (n = 67) (n, %)	TL (n = 1125) (n, %)	*P-* value
Age (mean ± SD)	30.42 ± 3.75	30.36 ± 3.79	0.862
<25	3 (4.48)	39 (3.47)	
25-34	54 (80.6)	924 (82.13)	
≥35	10 (14.93)	162 (14.4)	
BMI (mean ± SD)	26.12 ± 2.69	25.94 ± 5.37	<0.001
<25	22 (33.84)	562 (49.96)	
25-29.9	38 (56.72)	338 (30.04)	
≥30	7 (10.44)	225 (20)	
Delivery method			0.397
*Cesarean section*	24 (35.82)	356 (31.64)	
*Vaginal delivery*	43 (64.17)	769 (68.36)	
Delivery gestational week			
<28	6 (9)	–	
28–33^+6^	19 (28.36)	–	
34–36^+6^	42 (62.69)	–	
≥37	–	1,125	
*Clinical chorioamnionitis*	10 (14.93)	39 (3.47)	<0.001
*Histological* *chorioamnionitis*	24 (35.82)	55 (4.89)	<0.001
Neonatal weight (g)			
<1,000	6 (8.96)	–	
<1,500	12 (17.91)	–	
<2,500	49 (73.13)	20 (1.78)	<0.001
1-min Apgar scores			
8–10	56 (83.58)	1,104 (98.13)	<0.001
4-7	8 (11.94)	16 (1.42)	<0.001
0-3	3 (4.48)	5 (0.44)	<0.001

PPROM, preterm prelabor rupture of membranes; TL, normal full-term labor; BMI, body mass index.

### Vaginal discharge examination

Vaginal discharge examination and delivery had a time interval between 2 and 20 days before PPROM in the PPROM group. In the TL group, one pregnant woman presented with vaginal flora disorders, whereas the prevalence was higher in the PPROM group, with five cases (χ^2^ = 6, *P* < 0.001). We found that PPROM has a significant positive correlation with vaginal flora disorders (Spearman correlation coefficient: 0.42, *P* < 0.001). The proportion of PBC, leukocytes, toxic leukocytes, and bacteria with diameters smaller than 1.5 μm was also significantly higher in the PPROM group than that in the TL group (**Table 2**).

**Table 2:** Vaginal discharge imagines analyzed and compared using automatic analyzer GMD-600.

Number	PPROM (n = 67)/μL median (25% IQR, 75% IQR)	TL (n = 1,125)/μL median,(25% IQR, 75% IQR)	*P-* value
Epithelial cells	3,413.12 (2,654.03, 4,275.92)	3,395.92 (2,544.7, 4,458.61)	0.071
PBC	23.75 (11.85, 89.36)	10.28 (4.28, 20.29)	<0.001***
WBC	5,966.68 (3,227.85, 10,333.72)	3,077.64 (484.38, 4,107.1)	<0.001***
Toxic WBC	95 (38.75, 353.25)	75 (11.5, 232.5)	0.008**
Bacteria (s/l diameter)			
<1.5 μm	56,007 (37,595, 69,572)	31,671 (15,705, 43,139)	<0.001***
1.5~3 μm	83.56 (48.69, 194.45)	93 (35.12, 266.2)	0.75
3~4 μm	850.16 (467.55, 1,416.53)	820.84 (323.1, 2,635.03)	0.578
4~5 μm	1,181 (559.37, 1,942.71)	1,458.2 (518.39, 4,366.13)	0.018*
5~6 μm	551.13 (233.26, 1.039.05)	846.97 (279.4, 2,491.51)	0.002**
6~7 μm	483.75 (143.4, 993.47)	1,002 (270.61, 3,089.47)	<0.001***
7~8 μm	192.76 (48.7, 433.3)	554.94 (107.03, 1755.2)	<0.001***
8~9 μm	85.37 (23.8, 192.16)	228.56 (44.85, 803.56)	<0.001***
9~10 μm	23.75 (7.13, 73.05)	78.41 (15.95, 280.44)	<0.001***
>10 μm	13.67 (7.25, 22.6)	120.22 (27.43, 516.36)	<0.001***

PPROM, preterm prelabor rupture of membranes; TL, normal full term labor; PBC, parabasal cells; WBC, white blood cells; s/l diameter, short/long diameter. PPROM group vs. TL group; *P < 0.05, **P < 0.01, and ***P < 0.001.

### Vaginal opportunistic bacterial isolates

**Figure 2:**
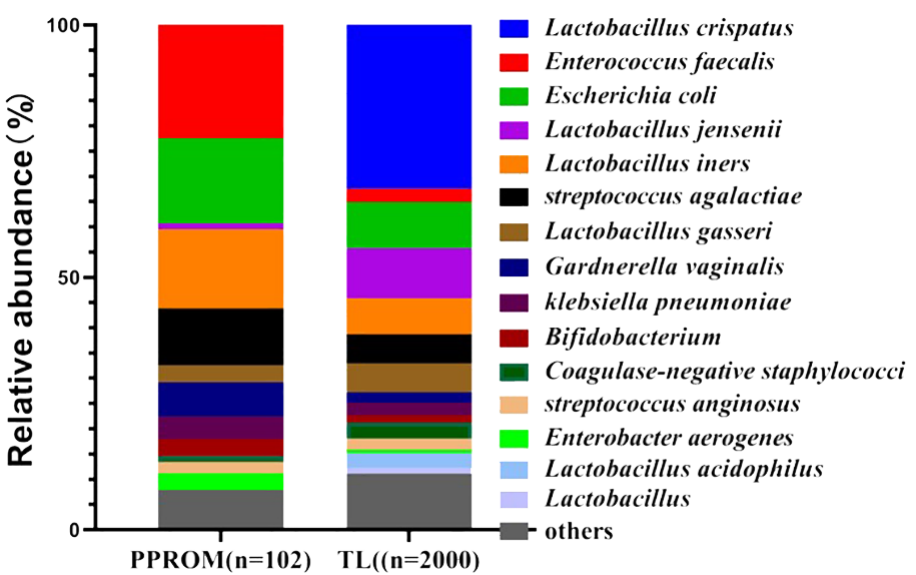
Vaginal microbiota based on the relative abundance of the PPROM group and TL group. PPROM, preterm prelabor rupture of membranes, TL, normal full term labor.

A total of 102 vaginal opportunistic bacterial isolates were collected from 67 pregnant women with PPROM. Among them, *Enterococcus faecalis* was the most common bacteria, comprising 21.57% (22/102) of total isolates. This was followed in order, and the proportion of *Escherichia coli* and *Lactobacillus iners* was 16.67% (17/102). In contrast, A total of 2,000 vaginal bacterial isolates were collected from 1,125 pregnant women in the TL group. The most common vaginal bacterial isolates identified were *Lactobacillus species.*, which included *Lactobacillus crispatus* (32.6%, 652/2000), *Lactobacillus jensenii* (20.8%, 199/2,000), and *Lactobacillus iners* (7.2%, 144/2,000). The proportion of *Escherichia coli* was 9.1% (182/2,000) (**Figure 2**).

### Drug sensitivity tests

The top three vaginal opportunistic bacterial isolates in all participants were *Escherichia coli* (9.47%, 199/2,102), *Streptococcus agalactiae* (5.99%, 126/2,102), and *Enterococcus faecalis* (3.57%, 75/2,102). Moreover, 16.08% (32/199) of *Escherichia coli* were extended-spectrum beta-lactamase producers, 36.51% (46/126) of *Streptococcus agalactiae* were multidrug-resistant organisms (MDROs), 20% (15/75) of *Enterococcus faecalis* were high-level streptomycin-resistant organisms, and 18.67% (14/75) of *Enterococcus faecalis* were high-level gentamicin-resistant organisms.

All isolates were sensitive to nitrofurantoin. In addition, *Escherichia coli* was highly susceptible to amikacin (100%), ertapenem (100%), imipenem (100%), cefepime (98.49%), piperacillin/tazobactam (97.99%), aztreonam (97.49%), cefotetan (96.98%), and ceftazidime (93.97%). However, *Streptococcus agalactiae* and *Enterococcus faecalis* display uniform high susceptibility to vancomycin (100%) and linezolid (100%). For *Streptococcus agalactiae*, no resistance to ampicillin, cefuroxime, ceftriaxone, cefotaxime, cefepime, meropenem, daptomycin, teicoplanin, cotrimoxazole, rifampin, penicillin, or amoxicillin-clavulanate was observed*. Enterococcus faecalis* isolates were susceptible to tigecycline (100%), ampicillin (100%), and penicillin (100%).

**Figure 3:**
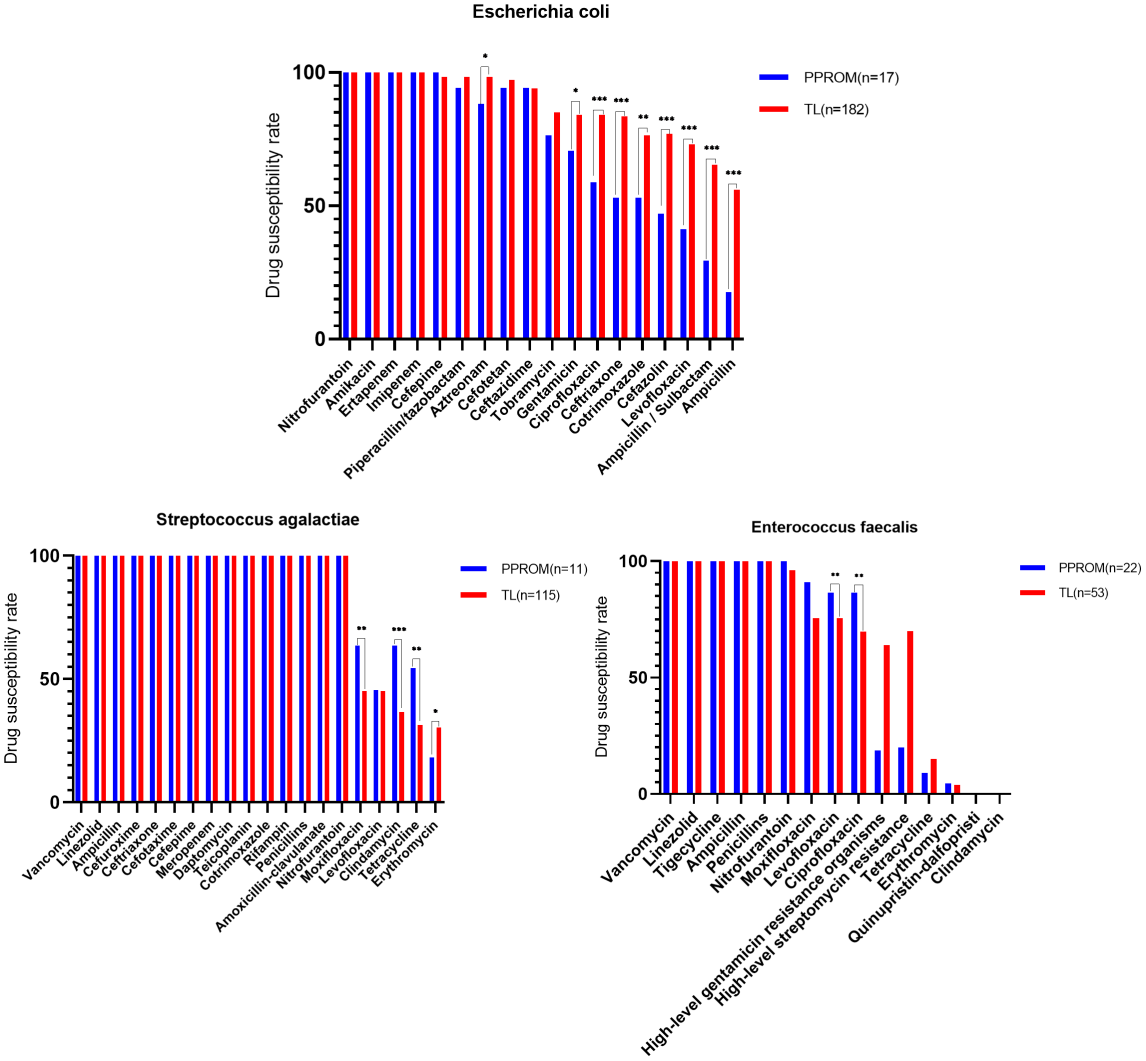
The top 3 vaginal opportunistic bacterial isolates tested for antimicrobial susceptibility of pregnant women. The rate of antimicrobial susceptibilities decreased from left to right: **(A)** *Escherichia coli* was susceptible to nitrofurantoin (100%) and the lowest sensitivity rate of 52.76% to ampicillin, **(B)** *Streptococcus agalactiae* was 100% sensitivity to vancomycin and the lowest sensitivity rate of 52.76% to Erythromycin, **(C)** *Enterococcus faecalis* is 100% sensitive to vancomycin and completely resistant to clindamycin.

However, *Escherichia coli* was resistant to ampicillin (52.76%), ampicillin/sulbactam (62.31%), and levofloxacin (70.35%). Notably, 36.51% of the *Streptococcus agalactiae* were classified as MDROs, with the most common MDR pattern being co–non-susceptibility to erythromycin (29.37%), tetracycline (33.33%), and clindamycin (38.89%). *Enterococcus faecalis* was resistant to clindamycin (0%), quinupristin-dalfopristin (0%), erythromycin (4%), and tetracycline (13.33%). The antimicrobial susceptibilities of *Escherichia coli* were performed using the AST-GN card. These isolates were 100% susceptible to nitrofurantoin, whereas the lowest sensitivity rate was 52.76% for ampicillin. The antimicrobial susceptibilities of *Streptococcus agalactiae* were determined using the TDR STR-AST detection reagent. These isolates were 100% susceptible to vancomycin, whereas the lowest sensitivity rate was 52.76% for erythromycin. The antimicrobial susceptibilities of the *Enterococcus faecalis* were performed using the AST-GP card. These isolates were 100% susceptible to vancomycin, and the lowest sensitivity rate was 52.76% for ampicillin. However, all isolates were resistant to clindamycin (**Figure 3**; **Table 3**).

**Table 3:** The drug sensitivity tests of the top three vaginal opportunistic bacterial isolates in vaginal secretion samples from the PPROM and TL groups.

Species	Medication names	Susceptibility results^#^	PPROM n (%)	TL n (%)	*P-* value
*Escherichia coli*			(n = 17)	(n = 182)	
	*cefepime*	S	17 (100)	179 (98.35)	0.497
		I	0	0	–
		R	0	3	–
	*Piperacillin*/tazobactam	S	16 (94.12)	179 (98.35)	0.479
		I	1	0	–
		R	0	3	–
	Cefotetan	S	16 (94.12)	177 (97.25)	0.498
		I	0	0	–
		R	1	5	–
	*Aztreonam*	S	15 (88.24)	179 (98.35)	0.013*
		I	0	0	–
		R	2	3	–
	*Ceftazidime*	S	16 (94.12)	171 (93.96)	1
		I	0	0	–
		R	1	11	–
	*Gentamicin*	S	12 (70.59)	153 (84.07)	0.028*
		I	0	0	–
		R	5	29	–
	*Levofloxacin*	S	7 (41.18)	133 (73.08)	<0.001***
		I	4	32	–
		R	6	17	–
	Tobramycin	S	13 (76.47)	155 (85.16)	0.108
		I	3	24	–
		R	1	3	–
	*Ciprofloxacin*	S	10 (58.82)	153 (84.07)	<0.001***
		I	0	4	–
		R	7	25	–
	Cotrimoxazole	S	9 (52.94)	139 (76.37)	0.001**
		I	0	0	–
		R	8	43	–
	Ceftriaxone	S	9 (52.94)	152 (83.52)	<0.001***
		I	0	0	–
		R	8	30	–
	*Cefazolin*	S	8 (47.06)	140 (76.92)	<0.001***
		I	0	0	–
		R	9	42	–
	*Ampicillin*/*Sulbactam*	S	5 (29.41)	119 (65.38)	<0.001***
		I	7	25	–
		R	5	38	–
	*Ampicillin*	S	3 (17.65)	102 (56.04)	<0.001***
		I	0	0	–
		R	14	80	–
*Streptococcus agalactiae*			(n = 11)	(n = 115)	
	Moxifloxacin	S	7 (63.64)	52 (45.22)	0.007**
		I	0	0	–
		R	4	63	–
	*Levofloxacin*	S	5 (45.45)	52 (45.22)	1
		I	0	0	–
		R	6	63	–
	*Clindamycin*	S	7 (63.64)	42 (36.52)	<0.001***
		I	0	1	–
		R	4	72	–
	Tetracycline	S	6 (54.55)	36 (31.30)	0.001**
		I	0	0	–
		R	5	79	–
	*Erythromycin*	S	2 (18.18)	35 (30.43)	0.033*
		I	0	1	–
		R	9	79	–
			(n = 22)	(n = 53)	
*Enterococcus faecalis*	Nitrofurantoin	S	22 (100)	51 (96.23)	0.121
		I	0	2	–
		R	0	0	–
	*Levofloxacin*	S	19 (86.36)	40 (75.47)	0.05
		I	0	1	–
		R	3	12	–
	Moxifloxacin	S	20 (90.91)	40 (75.47)	0.005**
		I	0	1	–
		R	2	12	–
	*Ciprofloxacin*	S	19 (86.36)	37 (69.81)	0.006**
		I	0	4	–
		R	3	12	–
	Tetracycline	S	2 (9.09)	8 (15.09)	0.277
		I	0	0	–
		R	20	45	–
	*Erythromycin*	S	1 (4.55)	2 (3.77)	0.733
		I	2	1	–
		R	19	50	–
	Quinupristin-dalfopristin	S	0 (0)	0 (0)	1
		I	0	0	–
		R	22	53	–
	*Clindamycin*	S	0 (0)	0 (0)	1
		I	0	0	–
		R	22	53	–

^#^The susceptibility results are determined according to the Clinical and Laboratory Standards Institute (CLSI) M100 guidelines for antimicrobial S testing. The susceptibility testing for *Escherichia coli* is performed using the Gram-negative bacteria susceptibility card AST-GN; the susceptibility testing for *Streptococcus agalactiae* is conducted using the TDR STR-AST *Streptococcus* detection kit; and the susceptibility testing for *Enterococcus faecalis* is carried out using the Gram-positive bacteria susceptibility card AST-GP.

PPROM, preterm prelabor rupture of membranes; TL, normal full term labor; S, susceptible; I, intermediate-resistant; R, resistant. The statistical analysis is conducted using the chi-square test, and, when the theoretical frequency is less than 1, the Fisher’s exact test is used. PPROM group **vs.** TL group; *P < 0.05, **P < 0.01, and ***P < 0.001.

### Bacterial profiles of vaginal specimens

A total of 80 VMD swab specimens underwent 16S rRNA gene amplification and sequencing. The 66 swab specimens of the pregnant women with VMD produced 4,898,416 reads, averaging 74,218 reads per sample. Unfortunately, 14 samples that did not satisfy the quality control criteria were excluded. A total of 4,436,059 high-quality sequences belonged to two groups, with a length distribution concentrated at 426–431, 408–413, and 420–425 bp. The flat Shannon–Wiener curve indicated that the sequencing data were sufficiently large to reflect most microbial information in these samples. A total of 2,884 operational taxonomic units (OTUs) were clustered into two groups. However, 1,986 (1,965~2,109) OTUs were detected in the VMD-PPROM (P) group, compared with 853 (799~909) OTUs in the VMD-TL (C) group. Venn analysis identified 241 common OTUs in the two groups, compared with 1,724 and 558 unique OTUs in the P and C groups, respectively.

**Figure 4:**
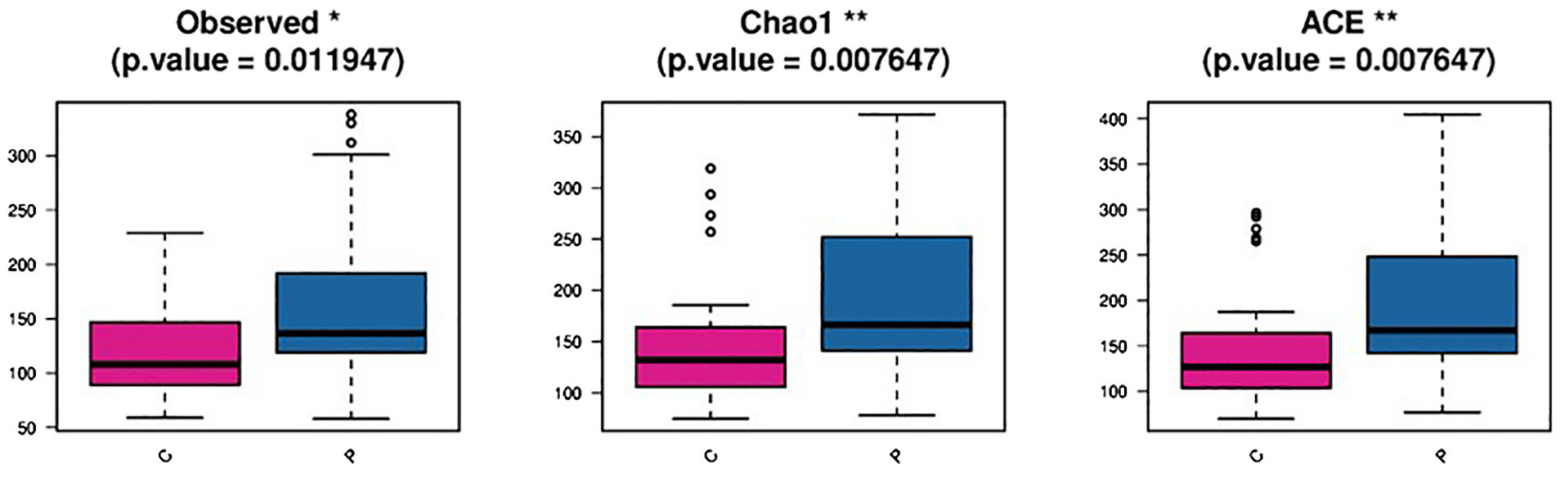
Diversity analysis among groups in the P and C group C: the vaginal microbiota dysbiosis of normal full term labor group, P: the vaginal microbiota dysbiosis of preterm prelabor rupture of membranes group.

The difference in vaginal flora alpha- and beta- diversity between the two groups was noticeable. Alpha- diversity from the directly observed OTUs revealed that the flora variety in the P group was greater than that in the C group. Alpha- diversity indices based on OTUs using Chao1 and abundance-based coverage estimates present a high richness of bacterial taxa in the P group, with the lowest values recorded for the C group. Beta-diversity index analysis revealed differences in vaginal microbiota across the groups through partial least squares discriminant analysis (**Figure 4**).

**Figure 5:**
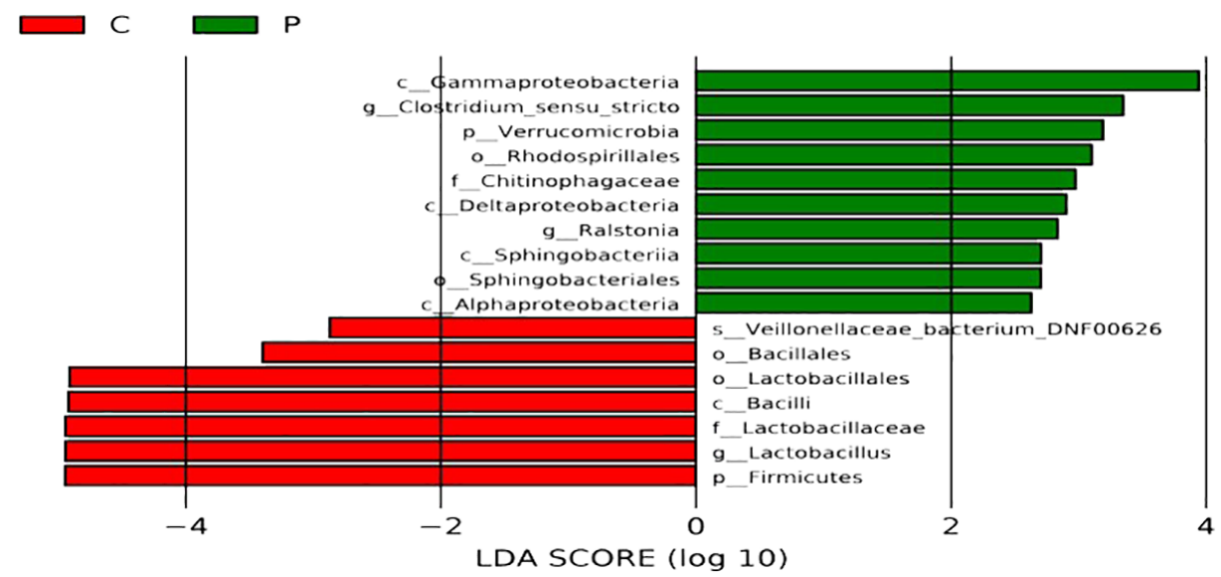
Analyses of differences for bacterial communities within groups. C: the vaginal microbiota dysbiosis of normal full term labor group, P: the vaginal microbiota dysbiosis of preterm prelabor rupture of membranes group. The Linear Discriminant Analysis Effect Size (LEfSe) method was used to select species that are most likely to explain the differences between groups. The non-parametric Kruskal-Wallis rank sum test was employed to screen for species with significantly different abundances between different groups; pairwise Wilcoxon rank sum tests were conducted for sub-group difference analysis; Linear Discriminant Analysis was used to assess the effect size of each significantly different species, that is, the LDA score log (10) with a larger absolute value indicating that the species is more likely to distinguish between groups. The bars of different colors represent the differential species in different groups, and the length of the bars is proportional to the size of the LDA score value.

Indicator species analysis revealed that *Lactobacillus jensenii, Lactobacillus crispatus*, and *Veillonellaceae bacterium DNF00626* were strongly associated with the C group. In contrast, the indicator bacteria in the P group were *Enterococcus faecalis, Escherichia coli*, and *Streptococcus agalactiae* (**Figure 5**).

## Conclusion

Our study indicated that 3.6% of the women experience PPROM, which is the upper end of the range reported in China. This can be attributed to two reasons: (1) as our hospital is a tertiary medical center for severe illness, referral bias cannot be entirely excluded; and (2) the prevalence of PPROM is increasing annually owing to environmental pollution, social pressure, and nutritional problems. Notably, BMI has been implicated as a factor contributing to PPROM. Specifically, both underweight and overweight and overweight conditions increase the risk of PPROM (Lynch et al., 2020). Our results regarding prepregnancy BMI demonstrated that more overweight pregnant women but fewer obese participants experienced PPROM. In the next step, a multicenter prospective longitudinal cohort will be designed. Comparable rates of cesarean deliveries were found among women with PPROM and those in the TL group. In contrast to the TL group, the PPROM group displayed a higher rate of chorioamnionitis. In addition, the PPROM group demonstrated a significantly lower proportion of newborns who weighed over 2,500 grams, and lower 1-min Apgar scores. These results paralleled previous literature reports (Stranik et al., 2021; Mengistu et al., 2022).

An abnormal vaginal microbiome, particularly a reduction of *Lactobacillus* species, is a significant risk factor closely related to PPROM (Pan et al., 2020; Yang et al., 2023). This study results also paralleled findings reported in the literature (Klerk et al., 2022), although in different ways. In this study, PPROM pregnant women were not predominantly colonized with *Group B Streptococcus*, but with various bacteria in the genital tract. However, despite no biomarkers being identified in the study, differences in bacterial abundance were observed in the abnormal vaginal flora of PPROM pregnancies. The main isolates identified in the bacterial cultures included *Enterococcus faecalis, Escherichia coli*, and *Streptococcus agalactiae.* However, high-throughput sequencing of the 16sRNA gene revealed *Enterococcus faecalis, Atopobium* sp.*S3PFAA1-4, Streptococcus anginosus, bifidobacterium longus, Streptococcus agalactiae*, and *Escherichia coli* as the primary isolates. In the literature, considerable differences exist, and four factors may have influenced these results. The first reason is the varying factors, such as ethnicity and geography (Ma et al., 2018; Pan et al., 2020; Tarca et al., 2021). The second factor is the inconsistent timing of urine sample collection during pregnancy (Pan et al., 2020; Bonasoni et al., 2022; Spiliopoulos et al., 2022). The third reason is the differences in the control group. The literature reports differ from this result (Wu et al., 2022); our control group was the VMD of the TL group, and mixed and abnormal vaginal microbes, such as *Gardnerella* and *Prevotella* species, were observed in both groups. The fourth reason is that routine prophylactic antibiotics used during pregnancy may have affected the diagnosis of early-onset neonatal sepsis in mothers with PPROM. Although PPROM has been linked to VMD, the use of antibiotics remains controversial. Studies have demonstrated that more aggressive antibiotic treatment for BV during pregnancy, including the administration of antibiotics such as clindamycin before 22 weeks, should result in a lower incidence of PPROM (Mohr et al., 2019; Ambalpady et al., 2022). However, other studies have highlighted that the abnormal vaginal bacteria can persist from PPROM to early delivery despite antibiotic treatment, and meta-analyses suggest that antibiotics may not prevent PPROM (Sullivan and Soper, 2015; Lorthe et al., 2020). Moreover, studies have indicated that prophylactic use of erythromycin can lead to an aggravated imbalance of vaginal microbiota in PPROM (Kovo et al., 2021).

The American College of Obstetricians and Gynecologists guidelines recommend the use of ampicillin/amoxicillin combined with erythromycin in PPROM pregnant women to prevent verticality spread (Faksh et al., 2011). However, some research in China has revealed that *Escherichia coli* in isolated bacteria from PPROM pregnant women’s membranes demonstrated resistance to tetracycline, ampicillin, piperacillin, trimethoprim-sulfamethoxazole, and cefazolin. In addition, *Enterococcus faecalis* was resistant to erythromycin, tetracycline, rifampicin, and ciprofloxacin, whereas *Streptococcus agalactiae* exhibited a high resistance rate to clindamycin, erythromycin, and tetracycline. Cefotaxime or the combination of ceftazidime and azithromycin improves maternal and infant outcomes in pregnant women with premature rupture of membranes at term (Hume-Nixon et al., 2021). The drug sensitivity test of vaginal secretions isolated from pregnant women in our study indicated that *Streptococcus agalactiae* is highly sensitive to ampicillin. However, *Escherichia coli* has low sensitivity to ampicillin, whereas *Streptococcus agalactiae* and *Enterococcus faecalis* are generally resistant to erythromycin, paralleling domestic research results (Bekele et al., 2022; Carey et al., 2022; Haindongo et al., 2022; Jiao et al., 2022). Considering that studies have indicated erythromycin to be effective in preventing PPROM, this study found that most bacterial strains isolated from the vaginal secretions of pregnant women were highly sensitive to antibiotics such as furantoin. As furantoin is classified as Class B in the FDA’s drug pregnancy safety classification, it may be a reasonable choice for managing in PPROM.

## Data Availability

The data presented in the study are deposited in the the Sequence Read Archive repository, accession number PRJNA1136794. This data has been published.
