# Supplementary material for: The microbiome biomarkers of pregnant women’s vaginal area predict preterm prelabor rupture in Western China
Source: Front Cell Infect Microbiol. 2024 Oct 31;14:1471027. doi: 10.3389/fcimb.2024.1471027 (PMC11560878; doi:10.3389/fcimb.2024.1471027)
Supplement: Supplementary file 1 [file DataSheet1.zip › compare_1/Community/KronaPlot/C19.krona.html]

Javascript must be enabled to view this page.

magnitude
magnitudeUnassigned

C19\_data\_for\_Krona

50717

50717

0

0

0

0

0

0

4468

0

0

0

0

0

0

0

0

0

0

0

0

0

0

4468

4468

0

0

0

0

0

0

170

170

8

162

0

0

0

0

0

0

0

0

0

0

0

0

3

3

0

0

0

0

0

0

3

0

0

0

0

0

0

0

4295

4295

0

1

0

0

177

0

11

0

23

0

0

0

2958

7

1118

0

0

0

0

0

0

0

0

0

0

0

0

0

0

0

0

0

0

0

0

0

0

0

0

0

0

0

0

0

0

0

0

0

0

0

0

0

0

0

0

0

0

0

0

0

0

0

0

0

0

0

0

0

0

0

0

0

0

0

0

0

0

0

0

0

0

0

0

0

0

0

0

0

0

0

0

0

0

0

0

0

0

0

0

0

0

0

0

0

0

0

0

0

0

0

0

0

0

0

0

0

0

0

0

0

0

0

0

0

0

0

0

0

0

0

0

0

0

0

0

0

0

0

0

0

0

0

0

0

0

0

0

0

57

57

55

21

21

13

8

34

0

0

16

0

0

16

0

0

18

18

0

0

0

0

0

0

0

0

0

0

0

0

1

1

1

1

1

1

1

0

0

1

0

0

0

0

0

0

0

0

0

0

0

0

0

0

0

0

0

0

0

0

0

0

0

0

0

0

0

8

3

0

0

0

0

0

0

0

0

0

0

0

0

0

0

0

0

0

0

0

0

0

0

0

0

0

3

3

3

3

0

0

0

0

0

0

0

0

0

0

0

0

0

0

0

0

0

0

0

0

0

0

0

0

0

0

0

0

0

0

0

0

0

0

0

0

0

0

0

0

0

0

0

0

0

0

0

0

0

0

0

0

0

0

0

0

0

0

5

0

0

0

0

1

1

1

1

0

0

0

0

0

4

4

4

4

0

0

0

0

0

0

0

0

0

0

0

0

0

0

0

0

0

0

0

0

0

0

0

0

0

0

0

0

0

0

0

0

0

0

0

0

0

0

0

0

0

0

0

0

0

0

0

0

0

0

0

0

0

0

0

0

0

0

0

46182

609

609

0

0

0

0

0

0

0

0

0

0

0

0

121

5

5

0

0

0

0

0

0

0

0

0

116

116

0

0

0

0

206

206

183

0

0

23

0

0

0

0

0

225

225

225

57

57

57

0

0

0

0

0

44176

44176

0

0

0

0

44176

44176

0

5

44102

69

0

0

0

0

0

0

0

1397

1397

0

0

0

1397

0

0

0

0

0

1397

0

1121

221

55

0

0

0

0

0

0

0

0

0

0

0

0

0

0

0

0

0

0

0

0

0

0

0

0

0

0

0

0

0

0

0

0

0

0

0

0

0

0

0

0

0

0

0

0

0

0

0

0

0

0

0

0

2

2

2

2

2

0

0

0

2

0

0

0

0

0

0

0

0

0

0

0
