# Supplementary material for: The microbiome biomarkers of pregnant women’s vaginal area predict preterm prelabor rupture in Western China
Source: Front Cell Infect Microbiol. 2024 Oct 31;14:1471027. doi: 10.3389/fcimb.2024.1471027 (PMC11560878; doi:10.3389/fcimb.2024.1471027)
Supplement: Supplementary file 1 [file DataSheet1.zip › compare_1/Community/KronaPlot/C23.krona.html]

Javascript must be enabled to view this page.

magnitude
magnitudeUnassigned

C23\_data\_for\_Krona

50717

50717

0

0

0

0

0

0

7282

0

0

0

0

0

0

0

0

0

0

0

0

0

0

7282

7282

0

0

0

0

0

0

122

117

0

0

0

0

0

117

0

0

0

0

5

5

0

0

28

28

0

5

0

0

0

23

0

0

0

0

0

0

0

0

7132

7132

0

2

0

0

0

0

0

0

0

3574

190

0

721

0

2645

0

0

0

0

0

0

0

0

0

0

0

0

0

0

0

0

0

0

0

0

0

0

0

0

0

0

0

0

0

0

0

3

3

3

3

3

0

0

3

0

0

0

0

0

0

0

0

0

0

0

0

0

0

0

0

0

0

0

0

0

0

0

0

0

0

0

0

0

0

0

0

0

0

0

0

0

0

0

0

0

0

0

0

0

0

0

0

0

0

0

0

0

0

0

0

0

0

0

0

0

0

0

0

0

0

0

0

0

0

0

0

0

0

0

0

0

0

0

0

0

0

0

0

0

0

0

0

0

0

0

0

0

0

3405

3405

34

0

0

0

0

34

0

0

8

0

3

5

0

0

26

26

0

0

0

0

0

0

0

0

0

0

0

0

0

0

0

0

0

0

0

0

0

0

3371

3371

0

0

3371

3367

0

0

4

0

0

0

0

0

0

0

0

0

0

0

0

0

0

0

0

0

0

4

1

0

0

0

0

0

0

0

0

0

0

0

0

0

0

0

0

0

0

0

0

0

0

0

0

0

0

0

0

0

0

1

1

1

1

0

0

0

0

3

0

0

0

0

3

0

0

0

0

3

0

0

3

3

0

0

0

0

0

0

0

0

0

0

0

0

0

0

0

0

0

0

0

0

0

0

0

0

0

0

0

0

0

0

0

0

0

0

0

0

0

0

0

0

0

0

0

0

0

0

0

0

0

0

0

0

0

0

0

0

0

0

0

0

0

0

0

0

0

0

0

0

0

0

0

0

0

0

0

0

0

0

0

0

0

0

0

0

0

0

0

0

0

0

0

0

0

0

0

0

0

0

0

0

0

0

0

0

0

0

0

0

0

0

0

40023

920

920

277

276

276

0

0

0

0

0

0

0

1

1

70

0

0

0

0

4

0

0

0

4

0

0

66

66

0

0

0

0

317

317

177

0

0

140

0

0

0

0

0

52

52

52

204

204

0

112

92

0

0

0

32450

32450

0

0

0

0

32450

32450

0

11

32355

84

0

0

0

0

0

0

0

6653

6653

0

0

0

6653

0

0

0

0

0

431

0

138

287

6

6222

0

0

0

6222

0

0

0

0

0

0

0

0

0

0

0

0

0

0

0

0

0

0

0

0

0

0

0

0

0

0

0

0

0

0

0

0

0

0

0

0

0

0

0

0

0

0

0

0

0

0

0

0

0

0

0

0

0

0

0

0

0

0

0

0

0

0

0

0

0

0

0
