# Supplementary material for: The microbiome biomarkers of pregnant women’s vaginal area predict preterm prelabor rupture in Western China
Source: Front Cell Infect Microbiol. 2024 Oct 31;14:1471027. doi: 10.3389/fcimb.2024.1471027 (PMC11560878; doi:10.3389/fcimb.2024.1471027)
Supplement: Supplementary file 1 [file DataSheet1.zip › compare_1/Community/KronaPlot/C8.krona.html]

Javascript must be enabled to view this page.

magnitude
magnitudeUnassigned

C8\_data\_for\_Krona

50717

50717

0

0

0

0

0

0

73

0

0

0

0

0

0

0

0

0

0

0

0

0

0

71

71

0

0

0

0

0

0

3

0

0

0

0

0

0

0

0

0

3

3

0

0

0

0

0

0

0

0

0

0

0

0

0

0

0

0

0

0

0

0

68

68

0

0

0

0

0

0

0

0

0

4

0

0

0

0

64

0

0

0

0

0

0

0

0

0

0

0

0

0

0

0

0

0

2

2

0

0

0

0

0

0

0

2

2

2

0

0

2

2

2

2

2

0

0

2

0

2

2

2

2

0

0

2

2

3

3

3

3

3

3

8

8

8

8

8

5

3

0

0

0

0

0

0

0

0

0

0

0

0

0

0

0

0

0

0

0

0

0

0

0

0

0

0

0

0

0

0

0

0

0

0

0

0

0

0

0

0

0

0

0

0

0

0

0

0

0

0

0

0

0

0

0

0

0

0

0

0

0

0

0

0

0

0

0

0

0

0

0

0

7

7

0

0

0

0

0

0

0

0

0

0

0

0

0

0

0

0

0

0

0

0

0

0

0

3

3

3

3

0

0

0

0

0

0

0

0

0

0

0

4

4

0

0

4

4

0

0

0

0

0

0

0

0

0

0

0

0

0

0

0

0

0

0

0

0

0

11

0

0

0

0

0

0

0

0

0

0

0

0

0

0

0

0

0

0

0

0

0

0

0

0

0

0

0

0

0

0

0

0

0

0

0

0

0

0

0

1

0

0

0

0

1

0

0

0

0

1

1

1

0

0

0

0

0

0

0

0

0

0

0

0

0

0

0

10

8

0

0

0

8

8

8

0

0

0

0

0

0

0

2

2

2

0

0

2

0

0

0

0

0

0

0

0

0

0

0

0

0

0

0

0

0

0

0

0

0

0

0

0

0

0

0

0

0

0

0

0

0

0

0

0

0

0

0

0

0

0

0

0

0

0

0

0

0

3

0

0

0

0

0

0

0

0

0

0

0

3

3

3

3

3

0

0

0

0

0

0

0

0

0

0

0

50601

107

107

0

0

0

0

0

0

0

0

0

0

0

0

35

0

0

0

0

0

0

0

0

0

0

0

35

35

0

2

2

2

6

6

6

0

0

0

0

0

0

0

0

21

21

21

43

43

43

0

0

0

0

0

50472

50472

9

9

9

0

50463

50463

0

6

36265

14192

0

0

0

0

0

0

0

22

22

0

0

0

22

0

0

0

0

0

22

0

0

0

22

0

0

0

0

0

0

0

0

0

0

0

0

0

0

0

0

0

5

0

0

0

0

0

0

0

0

0

0

5

5

5

0

0

5

5

0

0

0

0

0

0

0

0

0

0

0

0

0

0

0

0

0

2

2

2

2

2

0

0

0

0

0

0

0

2

0

0

0

0

0

0

0
