# Supplementary material for: The microbiome biomarkers of pregnant women’s vaginal area predict preterm prelabor rupture in Western China
Source: Front Cell Infect Microbiol. 2024 Oct 31;14:1471027. doi: 10.3389/fcimb.2024.1471027 (PMC11560878; doi:10.3389/fcimb.2024.1471027)
Supplement: Supplementary file 1 [file DataSheet1.zip › compare_1/Community/KronaPlot/P15.krona.html]

Javascript must be enabled to view this page.

magnitude
magnitudeUnassigned

P15\_data\_for\_Krona

50717

50717

0

0

0

0

0

0

44

0

0

0

0

0

0

0

0

0

0

0

0

0

0

40

40

5

5

5

0

0

0

5

0

0

0

0

0

0

0

0

0

0

0

5

5

0

0

0

0

0

0

0

0

0

0

0

0

0

0

0

0

0

0

30

30

0

0

0

0

0

0

0

0

0

27

0

3

0

0

0

0

0

0

0

0

0

0

0

0

4

4

4

4

0

3

0

1

0

0

0

0

0

0

0

0

0

0

0

0

0

0

14

14

14

14

14

10

0

4

0

3

3

3

3

0

0

3

3

0

0

0

0

0

0

51

51

51

51

51

38

13

0

0

0

0

0

0

0

0

0

0

0

0

0

0

0

0

0

0

0

0

0

0

0

0

0

0

0

0

0

0

0

0

0

0

0

0

0

0

0

0

0

0

0

0

0

0

0

0

0

11

11

11

11

11

0

11

0

0

0

0

20

20

20

20

20

20

0

0

0

0

0

0

7

7

7

0

0

0

0

3

0

0

3

0

0

0

0

3

0

0

4

4

4

0

0

0

0

0

0

0

0

0

0

0

0

0

0

0

0

0

0

0

0

0

0

0

0

0

0

0

0

0

0

0

0

0

0

0

0

0

0

0

0

0

0

0

0

0

0

149

24

16

13

13

13

3

3

3

0

0

0

0

0

0

0

0

0

0

0

0

0

0

0

0

0

0

0

0

0

0

0

8

8

8

8

0

0

0

0

9

0

0

0

0

0

0

0

0

0

0

0

0

0

0

0

0

0

2

2

2

0

0

2

7

7

7

7

97

92

0

0

0

92

92

92

0

0

0

0

0

0

0

5

5

5

0

5

0

19

0

0

0

0

10

0

0

0

10

10

5

5

0

8

8

8

8

0

0

0

0

0

0

0

0

1

1

1

0

0

0

1

0

0

0

0

0

0

0

0

0

0

6

6

6

6

6

6

10

0

0

0

0

0

2

2

2

2

2

0

8

8

8

0

0

8

0

0

8

0

0

0

0

0

0

0

50376

196

196

0

0

0

0

0

0

0

0

0

0

0

0

187

0

0

0

0

0

0

0

0

0

0

0

187

187

0

0

0

0

0

0

0

0

0

0

3

3

0

3

0

0

0

0

6

6

6

0

0

0

0

0

50177

50177

0

0

0

0

50177

50177

0

12

50081

84

0

0

0

0

0

0

0

3

3

0

0

0

3

0

0

0

3

3

0

0

0

0

0

0

0

0

0

0

0

0

0

0

0

0

0

0

0

0

0

0

20

8

8

0

0

0

8

8

0

8

0

4

4

4

0

0

0

0

4

4

0

0

8

8

8

8

4

4

0

0

0

0

0

0

0

6

6

6

6

6

6

0

0

0

0

0

0

0

0

0

0

0

0

0

0
