# Supplementary material for: The microbiome biomarkers of pregnant women’s vaginal area predict preterm prelabor rupture in Western China
Source: Front Cell Infect Microbiol. 2024 Oct 31;14:1471027. doi: 10.3389/fcimb.2024.1471027 (PMC11560878; doi:10.3389/fcimb.2024.1471027)
Supplement: Supplementary file 1 [file DataSheet1.zip › compare_1/Community/KronaPlot/P16.krona.html]

Javascript must be enabled to view this page.

magnitude
magnitudeUnassigned

P16\_data\_for\_Krona

50717

50715

4

4

4

4

4

4

98

7

7

2

2

0

2

0

0

5

0

0

0

5

5

83

83

0

0

0

0

0

0

4

0

0

0

0

0

0

0

4

4

0

0

0

0

0

0

74

74

0

20

0

36

10

0

2

0

0

6

0

0

0

0

5

5

0

0

0

0

0

0

0

0

0

5

0

0

0

0

0

0

0

0

0

0

0

0

0

0

0

0

0

0

0

0

0

0

8

8

5

3

3

0

0

2

2

3

3

3

0

0

26

26

26

26

26

0

5

18

3

18

18

18

18

11

11

7

7

0

0

0

0

0

0

115

73

73

73

73

52

21

4

4

4

4

0

4

4

4

4

4

4

0

0

0

0

0

0

0

0

0

0

0

0

0

0

7

7

7

7

7

27

27

27

27

27

0

0

0

0

0

0

0

0

0

0

0

0

0

0

11

11

11

11

11

0

8

3

0

0

0

21

21

21

21

21

21

3

3

3

3

3

3

48

48

8

0

0

0

0

0

0

0

0

0

0

0

0

0

0

0

8

8

8

2

2

2

2

0

0

0

0

0

0

0

0

0

31

31

31

8

23

0

7

7

0

0

7

7

0

0

0

0

0

0

0

0

0

7

3

3

3

3

3

4

4

4

4

2

2

88

20

3

0

0

0

3

0

0

3

3

0

0

0

0

0

0

0

0

3

3

3

3

0

0

0

0

0

0

0

0

0

14

14

14

14

0

0

0

0

39

0

0

0

0

39

12

12

12

0

27

0

0

0

0

27

0

27

0

0

0

0

0

0

0

0

0

0

8

4

0

0

0

0

0

0

4

4

4

0

0

0

0

4

4

4

4

0

0

16

0

0

0

0

10

0

0

0

10

10

6

0

4

0

0

0

0

0

0

0

0

0

0

0

0

6

6

6

3

0

3

0

0

0

0

5

5

5

5

0

2

3

24

24

24

24

24

24

11

3

3

3

3

3

6

6

6

6

2

4

2

2

2

0

0

2

0

2

0

0

0

0

0

0

0

0

50228

170

170

7

7

0

0

0

7

0

0

0

0

0

0

156

0

0

0

0

11

11

0

0

0

0

0

145

145

0

0

0

0

0

0

0

0

0

0

0

0

0

0

0

0

0

0

7

7

0

3

4

0

0

0

50056

50056

0

0

0

0

50052

50052

0

22

7605

42425

4

4

4

0

0

0

0

2

2

0

0

0

2

0

0

0

0

0

0

0

0

0

0

2

0

2

0

0

0

0

0

0

0

0

0

0

0

0

0

0

2

0

0

0

0

0

0

0

0

0

0

0

0

0

0

0

0

0

0

0

0

0

2

2

2

2

0

0

2

3

3

3

3

3

3

8

8

8

8

8

0

0

0

0

0

6

0

2

2

2

2

2

2

2

2
