# Supplementary material for: The microbiome biomarkers of pregnant women’s vaginal area predict preterm prelabor rupture in Western China
Source: Front Cell Infect Microbiol. 2024 Oct 31;14:1471027. doi: 10.3389/fcimb.2024.1471027 (PMC11560878; doi:10.3389/fcimb.2024.1471027)
Supplement: Supplementary file 1 [file DataSheet1.zip › compare_1/Community/KronaPlot/P18.krona.html]

Javascript must be enabled to view this page.

magnitude
magnitudeUnassigned

P18\_data\_for\_Krona

50717

50717

0

0

0

0

0

0

11259

0

0

0

0

0

0

0

0

0

0

0

0

0

0

11259

11259

0

0

0

0

0

0

0

0

0

0

0

0

0

0

0

0

0

0

0

0

0

0

4

4

0

0

0

0

0

0

4

0

0

0

0

0

0

0

11255

11255

0

0

0

0

0

0

0

0

0

5542

703

0

0

0

5010

0

0

0

0

0

0

0

0

0

0

0

0

0

0

0

0

0

0

0

0

0

0

0

0

0

0

0

0

0

0

0

0

0

0

0

0

0

0

0

0

0

0

0

0

0

0

0

0

0

0

0

0

0

0

0

0

0

0

0

0

0

0

0

0

0

0

0

0

0

0

0

0

0

0

0

0

0

0

0

0

0

0

0

0

0

0

0

0

0

0

0

0

0

0

0

0

0

0

0

0

0

0

0

0

0

0

0

0

0

0

0

0

0

0

0

0

0

0

0

0

0

0

0

0

0

0

0

0

0

0

0

0

0

2687

2687

2586

0

0

0

0

2586

0

0

138

0

138

0

0

0

2448

2448

0

0

0

0

0

0

0

8

8

8

8

0

0

0

0

0

0

0

0

0

0

0

93

93

0

0

0

0

0

0

0

0

0

93

93

0

0

0

0

0

0

0

0

0

0

0

0

0

0

1315

0

0

0

0

0

0

0

0

0

0

0

0

0

0

0

0

0

0

0

0

0

0

0

0

0

0

0

0

0

0

0

0

0

0

0

0

0

0

0

1302

1302

1302

1302

1302

0

0

0

0

0

0

0

0

0

0

0

0

0

0

0

0

0

0

0

0

0

0

0

13

13

0

0

0

13

13

13

0

0

0

0

0

0

0

0

0

0

0

0

0

0

0

0

0

0

0

0

0

0

0

0

0

0

0

0

0

0

0

0

0

0

0

0

0

0

0

0

0

0

0

0

0

0

0

0

0

0

0

0

0

0

0

0

0

0

0

0

0

0

0

0

0

0

0

0

0

0

0

0

0

0

0

0

0

0

0

0

0

0

0

0

0

0

0

0

0

0

35456

414

414

0

0

0

0

0

0

0

0

0

0

0

0

333

0

0

0

0

0

0

0

0

0

0

0

333

333

0

0

0

0

0

0

0

0

0

0

0

0

0

0

0

0

0

0

81

81

0

81

0

0

0

0

33392

33392

19009

19009

19009

0

14383

14383

0

10

14110

263

0

0

0

0

0

0

0

1650

1650

0

0

0

1650

0

0

0

0

0

1650

0

0

1363

287

0

0

0

0

0

0

0

0

0

0

0

0

0

0

0

0

0

0

0

0

0

0

0

0

0

0

0

0

0

0

0

0

0

0

0

0

0

0

0

0

0

0

0

0

0

0

0

0

0

0

0

0

0

0

0

0

0

0

0

0

0

0

0

0

0

0

0

0

0

0

0

0
