# Supplementary material for: The microbiome biomarkers of pregnant women’s vaginal area predict preterm prelabor rupture in Western China
Source: Front Cell Infect Microbiol. 2024 Oct 31;14:1471027. doi: 10.3389/fcimb.2024.1471027 (PMC11560878; doi:10.3389/fcimb.2024.1471027)
Supplement: Supplementary file 1 [file DataSheet1.zip › compare_1/Community/KronaPlot/P19.krona.html]

Javascript must be enabled to view this page.

magnitude
magnitudeUnassigned

P19\_data\_for\_Krona

50717

50717

0

0

0

0

0

0

7

0

0

0

0

0

0

0

0

0

0

0

0

0

0

7

7

0

0

0

0

0

0

0

0

0

0

0

0

0

0

0

0

0

0

0

0

0

0

0

0

0

0

0

0

0

0

0

0

0

0

0

0

0

0

7

7

0

0

0

0

0

0

0

0

0

7

0

0

0

0

0

0

0

0

0

0

0

0

0

0

0

0

0

0

0

0

0

0

0

0

0

0

0

0

0

0

0

0

0

0

0

0

0

0

0

0

0

0

0

0

0

0

0

0

0

0

0

0

0

0

0

0

0

0

0

11

8

8

8

8

4

4

0

0

0

0

0

0

0

0

0

0

0

0

0

0

0

0

0

0

0

3

3

3

3

3

0

0

0

0

0

0

0

0

0

0

0

0

0

0

0

0

0

0

0

0

0

0

0

0

0

2

2

2

2

2

2

0

0

0

0

0

2

2

2

2

2

2

0

0

0

0

0

0

2332

2332

5

0

0

0

0

0

0

0

0

0

0

0

0

0

0

0

5

5

5

0

0

0

0

13

13

13

13

0

0

0

0

0

0

0

0

0

0

0

2314

2314

0

0

2314

2314

0

0

0

0

0

0

0

0

0

0

0

0

0

0

0

0

0

0

0

0

0

21

0

0

0

0

0

0

0

0

0

0

0

0

0

0

0

0

0

0

0

0

0

0

0

0

0

0

0

0

0

0

0

0

0

0

0

0

0

0

0

2

0

0

0

0

0

0

0

0

0

0

0

0

0

0

0

0

0

2

2

2

2

0

0

0

0

0

0

16

13

0

0

0

13

13

13

0

0

0

0

0

0

0

3

3

3

0

3

0

3

0

0

0

0

3

0

0

0

3

3

3

0

0

0

0

0

0

0

0

0

0

0

0

0

0

0

0

0

0

0

0

0

0

0

0

0

0

0

0

0

0

0

2

2

2

2

2

2

1

0

0

0

0

0

0

0

0

0

0

0

1

1

1

0

0

1

1

0

0

0

0

0

0

0

0

0

48335

100

100

0

0

0

0

0

0

0

0

0

0

0

0

86

6

6

0

0

0

0

0

0

0

2

2

78

78

0

0

0

0

2

2

2

0

0

0

12

12

0

0

12

0

0

0

0

0

0

0

0

0

0

0

48235

48235

13

13

13

0

48219

48219

0

28

48044

147

3

3

3

0

0

0

0

0

0

0

0

0

0

0

0

0

0

0

0

0

0

0

0

0

0

0

0

0

0

0

0

0

0

0

0

0

0

0

0

0

4

2

2

0

0

0

2

2

2

0

0

2

2

2

0

0

2

2

0

0

0

0

0

0

0

0

0

0

0

0

0

0

0

0

0

0

0

0

0

0

0

0

0

0

0

0

0

0

0

0

0

0

0

0

0
