# Supplementary material for: The microbiome biomarkers of pregnant women’s vaginal area predict preterm prelabor rupture in Western China
Source: Front Cell Infect Microbiol. 2024 Oct 31;14:1471027. doi: 10.3389/fcimb.2024.1471027 (PMC11560878; doi:10.3389/fcimb.2024.1471027)
Supplement: Supplementary file 1 [file DataSheet1.zip › compare_1/Community/KronaPlot/P2.krona.html]

Javascript must be enabled to view this page.

magnitude
magnitudeUnassigned

P2\_data\_for\_Krona

50717

50717

0

0

0

0

0

0

76

0

0

0

0

0

0

0

0

0

0

0

0

0

0

76

76

0

0

0

0

0

0

0

0

0

0

0

0

0

0

0

0

0

0

0

0

0

0

6

6

0

0

0

0

0

6

0

0

0

0

0

0

0

0

70

70

0

0

0

0

0

0

0

0

0

39

6

0

0

0

25

0

0

0

0

0

0

0

0

0

0

0

0

0

0

0

0

0

0

0

0

0

0

0

0

0

0

0

0

0

0

0

0

0

0

0

0

0

0

0

0

0

0

0

0

0

0

0

0

0

0

0

0

0

0

6

6

6

6

6

6

0

0

0

0

0

0

0

0

0

0

0

0

0

0

0

0

0

0

0

0

0

0

0

0

0

0

0

0

0

0

0

0

0

0

0

0

0

0

0

0

0

0

0

0

0

0

0

0

0

0

0

0

0

0

0

0

0

0

0

0

0

0

0

0

0

0

0

0

0

0

0

0

0

69

69

2

0

0

0

0

2

0

0

2

0

0

2

0

0

0

0

0

0

0

0

0

0

0

9

9

9

9

0

0

0

0

0

0

0

0

0

0

0

58

58

0

0

58

58

0

0

0

0

0

0

0

0

0

0

0

0

0

0

0

0

0

0

0

0

0

28

0

0

0

0

0

0

0

0

0

0

0

0

0

0

0

0

0

0

0

0

0

0

0

0

0

0

0

0

0

0

0

0

0

0

0

0

0

0

0

2

0

0

0

0

0

0

0

0

0

0

0

0

0

0

0

0

0

2

2

2

0

2

0

0

0

0

0

22

18

0

0

0

18

18

18

0

0

0

0

0

0

0

4

4

4

0

4

0

4

0

0

0

0

0

0

0

0

0

0

0

0

0

0

0

0

0

0

0

0

0

0

0

0

0

4

4

4

0

0

0

0

0

4

0

0

0

0

0

0

0

0

0

0

0

0

0

0

0

0

0

0

0

0

0

0

0

0

0

0

0

0

0

0

0

0

0

0

0

0

0

0

0

0

0

0

50538

43

43

0

0

0

0

0

0

0

0

0

0

0

0

29

0

0

0

0

0

0

0

0

0

0

0

29

29

0

0

0

0

11

11

0

0

0

11

0

0

0

0

0

3

3

3

0

0

0

0

0

0

0

0

50478

50478

26

26

26

0

50342

50342

197

2864

2795

44486

110

110

110

0

0

0

0

17

17

0

0

0

17

0

0

0

0

0

12

0

0

0

12

5

5

0

0

0

0

0

0

0

0

0

0

0

0

0

0

0

0

0

0

0

0

0

0

0

0

0

0

0

0

0

0

0

0

0

0

0

0

0

0

0

0

0

0

0

0

0

0

0

0

0

0

0

0

0

0

0

0

0

0

0

0

0

0

0

0

0

0

0

0

0

0
