# Supplementary material for: The microbiome biomarkers of pregnant women’s vaginal area predict preterm prelabor rupture in Western China
Source: Front Cell Infect Microbiol. 2024 Oct 31;14:1471027. doi: 10.3389/fcimb.2024.1471027 (PMC11560878; doi:10.3389/fcimb.2024.1471027)
Supplement: Supplementary file 1 [file DataSheet1.zip › compare_1/Community/KronaPlot/P21.krona.html]

Javascript must be enabled to view this page.

magnitude
magnitudeUnassigned

P21\_data\_for\_Krona

50717

50717

0

0

0

0

0

0

976

0

0

0

0

0

0

0

0

0

0

0

0

0

0

976

976

0

0

0

0

0

0

0

0

0

0

0

0

0

0

0

0

0

0

0

0

0

0

8

8

8

0

0

0

0

0

0

0

0

0

0

0

0

0

968

968

0

0

0

0

0

0

0

0

0

0

0

0

0

0

968

0

0

0

0

0

0

0

0

0

0

0

0

0

0

0

0

0

0

0

0

0

0

0

0

0

0

0

0

0

0

0

0

0

0

0

0

0

0

0

0

0

0

0

0

0

0

0

0

0

0

0

0

0

0

0

0

0

0

0

0

0

0

0

0

0

0

0

0

0

0

0

0

0

0

0

0

0

0

0

0

0

0

0

0

0

0

0

0

0

0

0

0

0

0

0

0

0

0

0

0

0

0

0

0

0

0

0

0

0

0

0

0

0

0

0

0

0

0

0

0

0

0

0

0

0

0

0

0

0

0

0

0

0

153

153

146

0

0

0

0

146

0

0

146

0

0

146

0

0

0

0

0

0

0

0

0

0

0

0

0

0

0

0

0

0

0

0

0

0

0

0

0

0

7

7

0

0

4

4

0

0

0

3

3

0

0

0

0

0

0

0

0

0

0

0

0

0

0

0

0

0

0

0

0

0

0

0

0

0

0

0

0

0

0

0

0

0

0

0

0

0

0

0

0

0

0

0

0

0

0

0

0

0

0

0

0

0

0

0

0

0

0

0

0

0

0

0

0

0

0

0

0

0

0

0

0

0

0

0

0

0

0

0

0

0

0

0

0

0

0

0

0

0

0

0

0

0

0

0

0

0

0

0

0

0

0

0

0

0

0

0

0

0

0

0

0

0

0

0

0

0

0

0

0

0

0

0

0

0

0

0

0

0

0

0

0

0

0

0

0

0

0

0

0

0

0

0

0

0

0

0

0

0

0

0

0

0

0

0

0

0

0

0

0

0

0

0

0

0

0

0

0

0

0

0

0

0

0

0

0

0

0

0

0

0

0

49588

0

0

0

0

0

0

0

0

0

0

0

0

0

0

0

0

0

0

0

0

0

0

0

0

0

0

0

0

0

0

0

0

0

0

0

0

0

0

0

0

0

0

0

0

0

0

0

0

0

0

0

0

0

0

49316

49316

36

36

36

0

49280

49280

0

5

49240

35

0

0

0

0

0

0

0

272

272

0

0

0

272

0

0

0

0

0

0

0

0

0

0

272

0

0

272

0

0

0

0

0

0

0

0

0

0

0

0

0

0

0

0

0

0

0

0

0

0

0

0

0

0

0

0

0

0

0

0

0

0

0

0

0

0

0

0

0

0

0

0

0

0

0

0

0

0

0

0

0

0

0

0

0

0

0

0

0

0

0

0

0

0

0

0
