# Supplementary material for: The microbiome biomarkers of pregnant women’s vaginal area predict preterm prelabor rupture in Western China
Source: Front Cell Infect Microbiol. 2024 Oct 31;14:1471027. doi: 10.3389/fcimb.2024.1471027 (PMC11560878; doi:10.3389/fcimb.2024.1471027)
Supplement: Supplementary file 1 [file DataSheet1.zip › compare_1/Community/KronaPlot/P22.krona.html]

Javascript must be enabled to view this page.

magnitude
magnitudeUnassigned

P22\_data\_for\_Krona

50717

50717

0

0

0

0

0

0

1054

0

0

0

0

0

0

0

0

0

0

0

0

0

0

1054

1054

0

0

0

0

0

0

2

0

0

0

0

0

0

0

0

0

0

0

2

0

0

2

0

0

0

0

0

0

0

0

0

0

0

0

0

0

0

0

1052

1052

0

0

0

0

0

0

0

0

0

986

46

0

0

0

20

0

0

0

0

0

0

0

0

0

0

0

0

0

0

0

0

0

0

0

0

0

0

0

0

0

0

0

0

0

0

0

0

0

0

0

0

0

0

0

0

0

0

0

0

0

0

0

0

0

0

0

0

0

0

0

0

0

0

0

0

0

0

0

0

0

0

0

0

0

0

0

0

0

0

0

0

0

0

0

0

0

0

0

0

0

0

0

0

0

0

0

0

0

0

0

0

0

0

0

0

0

0

0

0

0

0

0

0

0

0

0

0

0

0

0

0

0

0

0

0

0

0

0

0

0

0

0

0

0

0

0

0

0

1

1

0

0

0

0

0

0

0

0

0

0

0

0

0

0

0

0

0

0

0

0

0

0

0

0

0

0

0

0

0

0

0

0

0

0

0

0

0

0

1

1

1

1

0

0

0

0

0

0

0

0

0

0

0

0

0

0

0

0

0

0

0

0

0

0

0

9

0

0

0

0

0

0

0

0

0

0

0

0

0

0

0

0

0

0

0

0

0

0

0

0

0

0

0

0

0

0

0

0

0

0

0

0

0

0

0

0

0

0

0

0

0

0

0

0

0

0

0

0

0

0

0

0

0

0

0

0

0

0

0

0

0

0

0

9

7

0

0

0

7

7

7

0

0

0

0

0

0

0

2

2

2

2

0

0

0

0

0

0

0

0

0

0

0

0

0

0

0

0

0

0

0

0

0

0

0

0

0

0

0

0

0

0

0

0

0

0

0

0

0

0

0

0

0

0

0

0

0

0

0

0

0

0

0

0

0

0

0

0

0

0

0

0

0

0

0

0

0

0

0

0

0

0

0

0

0

0

0

0

0

0

0

49653

14

14

0

0

0

0

0

0

0

0

0

0

0

0

11

0

0

0

0

0

0

0

0

0

0

0

11

11

0

0

0

0

3

3

0

0

0

3

0

0

0

0

0

0

0

0

0

0

0

0

0

0

0

0

49583

49583

10

10

10

0

49573

49573

0

8

11221

38344

0

0

0

0

0

0

0

52

52

0

0

0

52

0

0

0

0

0

52

0

0

0

52

0

0

0

0

0

0

0

0

0

0

4

4

4

0

0

4

4

0

0

0

0

0

0

0

0

0

0

0

0

0

0

0

0

0

0

0

0

0

0

0

0

0

0

0

0

0

0

0

0

0

0

0

0

0

0

0

0

0

0

0

0

0

0

0

0

0

0

0

0

0

0

0
