# Supplementary material for: The microbiome biomarkers of pregnant women’s vaginal area predict preterm prelabor rupture in Western China
Source: Front Cell Infect Microbiol. 2024 Oct 31;14:1471027. doi: 10.3389/fcimb.2024.1471027 (PMC11560878; doi:10.3389/fcimb.2024.1471027)
Supplement: Supplementary file 1 [file DataSheet1.zip › compare_1/Community/KronaPlot/P23.krona.html]

Javascript must be enabled to view this page.

magnitude
magnitudeUnassigned

P23\_data\_for\_Krona

50717

50717

0

0

0

0

0

0

27

1

1

1

0

0

0

1

1

0

0

0

0

0

0

22

22

0

0

0

0

0

0

0

0

0

0

0

0

0

0

0

0

0

0

0

0

0

0

18

18

0

0

0

0

0

18

0

0

0

0

0

0

0

0

4

4

0

0

0

0

0

0

0

0

0

4

0

0

0

0

0

0

0

0

0

0

0

0

0

0

0

0

0

0

0

0

0

0

4

4

4

0

0

2

2

2

2

0

0

0

0

0

0

0

0

0

0

0

0

0

0

0

0

0

0

0

0

0

0

0

0

0

0

0

0

0

0

0

0

0

0

0

0

0

0

0

0

0

0

0

0

0

0

0

0

0

0

0

0

0

0

0

0

0

0

0

0

0

0

0

0

0

0

0

0

0

0

0

0

0

0

0

0

0

0

0

0

0

0

0

0

4

4

4

4

4

0

0

0

2

0

2

3

3

3

3

3

3

0

0

0

0

0

0

7

7

0

0

0

0

0

0

0

0

0

0

0

0

0

0

0

0

0

0

0

3

3

3

3

0

0

0

0

0

0

0

0

0

0

0

0

0

0

0

4

4

0

0

4

4

0

0

0

0

0

0

0

0

0

2

0

0

0

0

0

2

2

2

2

0

2

8

4

3

0

0

0

3

3

3

0

0

0

0

0

0

0

0

0

0

0

0

0

0

0

0

0

0

0

0

0

0

0

1

1

1

1

0

0

0

0

1

0

0

0

0

1

0

0

0

0

1

0

0

0

0

1

1

0

0

0

0

0

0

0

0

0

0

0

3

3

3

3

3

0

0

0

0

0

0

0

0

0

0

0

0

0

0

0

0

0

0

0

0

0

0

0

0

0

0

0

0

0

0

0

0

0

0

0

0

0

0

0

0

0

0

0

0

0

0

0

0

0

0

0

0

0

0

0

0

0

0

0

2

2

2

2

2

2

0

0

0

0

0

0

0

0

0

0

0

0

0

0

0

0

0

0

0

0

0

0

0

0

0

0

0

0

50659

34

34

0

0

0

0

0

0

0

0

0

0

0

0

34

3

3

0

0

0

0

0

0

0

0

0

31

31

0

0

0

0

0

0

0

0

0

0

0

0

0

0

0

0

0

0

0

0

0

0

0

0

0

0

50552

50552

0

0

0

0

50447

50447

0

14

7772

42661

105

105

105

0

0

0

0

73

73

0

0

0

73

73

0

73

0

0

0

0

0

0

0

0

0

0

0

0

0

0

0

0

0

0

0

0

0

0

0

0

4

2

2

2

2

2

0

0

0

0

0

0

0

0

0

0

0

0

0

0

0

0

2

2

2

2

0

0

2

0

0

0

0

0

0

1

1

1

1

1

0

0

0

0

0

1

0

0

0

0

0

0

0

0

0
