# Supplementary material for: The microbiome biomarkers of pregnant women’s vaginal area predict preterm prelabor rupture in Western China
Source: Front Cell Infect Microbiol. 2024 Oct 31;14:1471027. doi: 10.3389/fcimb.2024.1471027 (PMC11560878; doi:10.3389/fcimb.2024.1471027)
Supplement: Supplementary file 1 [file DataSheet1.zip › compare_1/Community/KronaPlot/P26.krona.html]

Javascript must be enabled to view this page.

magnitude
magnitudeUnassigned

P26\_data\_for\_Krona

50717

50717

0

0

0

0

0

0

123

2

2

0

0

0

0

0

0

2

2

0

2

0

0

120

120

0

0

0

0

0

0

13

8

0

6

2

0

0

0

0

0

0

0

5

0

5

0

36

36

6

0

2

0

0

15

0

0

13

0

0

0

0

0

71

71

13

0

0

0

0

0

0

0

0

13

6

0

13

0

26

0

0

0

0

0

0

0

0

0

1

1

1

1

0

0

1

0

0

0

0

0

0

0

0

0

0

0

0

0

0

0

0

0

0

0

0

0

0

0

0

0

0

0

0

0

0

0

0

0

0

0

0

0

0

4

0

0

0

0

0

0

0

0

0

0

0

0

0

0

0

0

0

0

0

0

0

0

0

0

0

0

0

0

0

0

0

0

0

0

0

0

4

4

4

4

4

0

0

0

0

0

0

0

0

0

0

0

0

0

0

0

0

0

0

0

0

0

0

0

0

0

0

0

0

0

0

0

0

0

0

0

0

0

41

41

0

0

0

0

0

0

0

0

0

0

0

0

0

0

0

0

0

0

0

0

0

0

0

8

8

8

8

0

0

0

0

0

0

0

0

0

0

0

33

33

0

0

33

33

0

0

0

0

0

0

0

0

0

0

0

0

0

0

0

0

0

0

0

0

0

145

18

0

0

0

0

0

0

0

0

0

3

3

3

3

6

6

6

6

0

0

0

0

0

0

0

0

0

0

0

0

0

9

9

9

9

0

0

0

0

10

0

0

0

0

10

0

0

0

0

10

0

0

5

5

5

5

0

0

0

0

0

0

0

0

0

0

0

115

115

0

0

0

115

115

115

0

0

0

0

0

0

0

0

0

0

0

0

0

2

0

0

0

0

0

0

0

0

0

0

0

0

0

0

0

0

0

0

0

0

0

0

0

0

0

2

2

2

0

0

0

0

0

0

2

0

0

0

0

0

0

0

0

0

0

0

0

0

0

0

0

0

0

0

0

0

0

0

0

0

0

0

0

0

0

0

0

0

0

0

0

0

0

0

0

0

50389

136

136

13

11

8

3

0

0

0

0

0

0

2

2

78

0

0

0

0

0

0

0

0

0

0

0

78

78

0

0

0

0

0

0

0

0

0

0

15

15

15

0

0

0

0

0

30

30

30

0

0

0

0

0

50229

50224

0

0

0

0

50221

50221

210

29

49626

356

3

3

3

5

5

5

5

24

24

0

0

0

24

0

0

0

0

0

24

0

17

7

0

0

0

0

0

0

0

0

0

0

0

0

0

0

0

0

0

0

9

3

3

0

0

0

3

3

0

0

3

4

4

4

0

0

0

0

4

0

0

4

2

2

2

2

0

0

2

0

0

0

0

0

0

6

6

6

6

6

0

0

0

2

4

0

0

0

0

0

0

0

0

0

0
