# Supplementary material for: The microbiome biomarkers of pregnant women’s vaginal area predict preterm prelabor rupture in Western China
Source: Front Cell Infect Microbiol. 2024 Oct 31;14:1471027. doi: 10.3389/fcimb.2024.1471027 (PMC11560878; doi:10.3389/fcimb.2024.1471027)
Supplement: Supplementary file 1 [file DataSheet1.zip › compare_1/Community/KronaPlot/P28.krona.html]

Javascript must be enabled to view this page.

magnitude
magnitudeUnassigned

P28\_data\_for\_Krona

50717

50717

0

0

0

0

0

0

956

0

0

0

0

0

0

0

0

0

0

0

0

0

0

956

956

0

0

0

3

3

3

93

93

0

16

77

0

0

0

0

0

0

0

0

0

0

0

19

19

4

0

0

0

0

6

6

3

0

0

0

0

0

0

841

841

0

2

0

0

0

0

0

0

25

8

33

0

0

0

773

0

0

0

0

0

0

0

0

0

0

0

0

0

0

0

0

0

0

0

0

0

0

0

0

0

0

0

0

0

0

0

0

0

0

0

0

0

0

0

0

0

0

0

0

0

0

0

0

0

0

0

0

0

0

0

0

0

0

0

0

0

0

0

0

0

0

0

0

0

0

0

0

0

0

0

0

0

0

0

0

0

0

0

0

0

0

0

0

0

0

0

0

0

0

0

0

0

0

0

0

0

0

0

0

0

0

0

0

0

0

0

0

0

0

0

0

0

0

0

0

0

0

0

0

0

0

0

0

0

0

0

0

0

90

90

9

0

0

0

0

9

0

0

9

9

0

0

0

0

0

0

0

0

0

0

0

0

0

0

0

0

0

0

0

0

0

0

0

0

0

0

0

0

81

81

0

0

74

33

0

0

41

0

0

7

0

0

7

0

0

0

0

0

0

0

0

0

0

0

0

4

0

0

0

0

0

0

0

0

0

0

0

0

0

0

0

0

0

0

0

0

0

0

0

0

0

0

0

0

0

0

0

0

0

0

0

0

0

0

0

1

1

1

1

1

0

0

0

0

0

0

0

0

0

0

0

0

0

0

0

0

0

0

0

0

0

0

0

0

0

0

0

0

0

0

0

0

0

0

0

0

0

0

0

0

0

0

0

0

3

3

3

3

3

0

0

0

0

0

0

0

0

0

0

0

0

0

0

0

0

0

0

0

0

0

0

0

0

0

0

0

0

0

0

0

0

0

0

0

0

0

0

0

0

0

0

0

0

0

0

0

0

0

0

0

0

0

0

0

0

0

0

0

0

0

0

0

0

0

0

0

0

0

0

0

0

49667

898

898

4

4

0

0

4

0

0

0

0

0

0

0

329

0

0

0

0

0

0

0

0

0

0

0

329

329

0

0

0

0

31

31

8

2

0

21

0

0

0

0

0

511

511

511

18

18

18

0

0

5

5

5

48570

48570

0

0

0

0

48570

48570

0

6

17525

31039

0

0

0

0

0

0

0

199

199

0

0

0

199

0

0

0

0

0

199

0

0

19

180

0

0

0

0

0

0

0

0

0

0

0

0

0

0

0

0

0

0

0

0

0

0

0

0

0

0

0

0

0

0

0

0

0

0

0

0

0

0

0

0

0

0

0

0

0

0

0

0

0

0

0

0

0

0

0

0

0

0

0

0

0

0

0

0

0

0

0

0

0

0

0

0
