# Supplementary material for: The microbiome biomarkers of pregnant women’s vaginal area predict preterm prelabor rupture in Western China
Source: Front Cell Infect Microbiol. 2024 Oct 31;14:1471027. doi: 10.3389/fcimb.2024.1471027 (PMC11560878; doi:10.3389/fcimb.2024.1471027)
Supplement: Supplementary file 1 [file DataSheet1.zip › compare_1/Community/KronaPlot/P3.krona.html]

Javascript must be enabled to view this page.

magnitude
magnitudeUnassigned

P3\_data\_for\_Krona

50717

50717

0

0

0

0

0

0

86

0

0

0

0

0

0

0

0

0

0

0

0

0

0

86

86

0

0

0

0

0

0

6

6

0

6

0

0

0

0

0

0

0

0

0

0

0

0

0

0

0

0

0

0

0

0

0

0

0

0

0

0

0

0

80

80

3

0

0

0

0

0

0

0

2

65

10

0

0

0

0

0

0

0

0

0

0

0

0

0

0

0

0

0

0

0

0

0

0

0

0

0

0

0

0

0

0

0

0

0

0

0

0

0

0

0

0

0

0

0

0

0

0

0

0

0

0

0

0

0

0

0

0

0

0

0

0

0

0

0

0

0

0

0

0

0

0

0

0

0

0

0

0

0

0

0

0

0

0

0

0

0

0

0

0

0

0

0

0

0

0

0

0

0

0

0

0

0

0

0

0

0

0

0

0

0

0

0

0

0

0

0

0

0

0

0

0

0

0

0

0

0

0

0

0

0

0

0

0

0

0

0

0

0

4151

4151

0

0

0

0

0

0

0

0

0

0

0

0

0

0

0

0

0

0

0

0

0

0

0

0

0

0

0

0

0

0

0

0

0

0

0

0

0

0

4151

4151

0

0

4151

4151

0

0

0

0

0

0

0

0

0

0

0

0

0

0

0

0

0

0

0

0

0

15

0

0

0

0

0

0

0

0

0

0

0

0

0

0

0

0

0

0

0

0

0

0

0

0

0

0

0

0

0

0

0

0

0

0

0

0

0

0

0

0

0

0

0

0

0

0

0

0

0

0

0

0

0

0

0

0

0

0

0

0

0

0

0

0

0

0

0

15

12

0

0

0

12

12

12

0

0

0

0

0

0

0

3

3

3

2

0

1

0

0

0

0

0

0

0

0

0

0

0

0

0

0

0

0

0

0

0

0

0

0

0

0

0

0

0

0

0

0

0

0

0

0

0

0

0

0

0

0

0

0

0

0

0

0

0

0

0

0

0

0

0

0

0

0

0

0

0

0

0

0

0

0

0

0

0

0

0

0

0

0

0

0

0

0

0

46463

14

14

0

0

0

0

0

0

0

0

0

0

0

0

14

0

0

0

0

0

0

0

0

0

0

0

14

14

0

0

0

0

0

0

0

0

0

0

0

0

0

0

0

0

0

0

0

0

0

0

0

0

0

0

46439

46439

29

29

29

0

46410

46410

0

14

46273

123

0

0

0

0

0

0

0

10

10

0

0

0

10

0

0

0

0

0

6

0

0

0

6

4

0

0

0

4

0

0

0

0

0

0

0

0

0

0

0

0

2

2

2

0

0

0

2

2

2

0

0

0

0

0

0

0

0

0

0

0

0

0

0

0

0

0

0

0

0

0

0

0

0

0

0

0

0

0

0

0

0

0

0

0

0

0

0

0

0

0

0

0

0

0

0
