# Supplementary material for: The microbiome biomarkers of pregnant women’s vaginal area predict preterm prelabor rupture in Western China
Source: Front Cell Infect Microbiol. 2024 Oct 31;14:1471027. doi: 10.3389/fcimb.2024.1471027 (PMC11560878; doi:10.3389/fcimb.2024.1471027)
Supplement: Supplementary file 1 [file DataSheet1.zip › compare_1/Community/KronaPlot/P30.krona.html]

Javascript must be enabled to view this page.

magnitude
magnitudeUnassigned

P30\_data\_for\_Krona

50717

50717

0

0

0

0

0

0

5279

0

0

0

0

0

0

0

0

0

0

0

0

0

0

5082

5082

0

0

0

0

0

0

196

196

0

196

0

0

0

0

0

0

0

0

0

0

0

0

0

0

0

0

0

0

0

0

0

0

0

0

0

0

0

0

4886

4886

0

0

20

91

0

0

0

0

0

32

0

0

0

0

4743

197

197

197

197

197

0

0

0

0

0

0

0

0

0

0

0

0

0

0

0

0

0

0

0

0

0

0

0

0

0

0

0

0

0

0

0

0

0

0

0

0

0

0

0

0

0

0

0

0

0

0

0

0

0

0

0

0

0

0

0

0

0

0

0

0

0

0

0

0

0

0

0

0

0

0

0

0

0

0

0

0

0

0

0

0

0

0

0

0

0

0

0

0

0

0

0

0

0

0

0

0

0

0

0

0

0

0

0

0

0

0

0

0

0

0

0

0

0

0

0

0

2

2

2

2

2

2

0

0

0

0

0

0

38666

38666

51

0

0

0

0

51

0

0

51

0

0

51

0

0

0

0

0

0

0

0

0

0

0

38604

38604

38604

38604

0

0

0

0

0

0

0

0

0

0

0

11

11

11

11

0

0

0

0

0

0

0

0

0

0

0

0

0

0

0

0

0

0

0

0

0

0

0

2

0

0

0

0

0

0

0

0

0

0

0

0

0

0

0

0

0

0

0

0

0

0

0

0

0

0

0

0

0

0

0

0

0

0

0

0

0

0

0

0

0

0

0

0

0

0

0

0

0

0

0

0

0

0

0

0

0

0

0

0

0

0

0

0

0

0

0

2

0

0

0

0

0

0

0

0

0

0

2

2

2

2

0

0

0

0

0

0

0

0

0

0

0

0

0

0

0

0

0

0

0

0

0

0

0

0

0

0

0

0

0

0

0

0

0

0

0

0

0

0

0

0

0

0

0

0

0

0

0

0

0

0

0

0

0

0

0

0

0

0

0

0

0

0

0

0

0

0

0

0

0

0

0

0

0

0

0

0

0

0

0

0

0

0

0

6768

2515

2515

0

0

0

0

0

0

0

0

0

0

0

0

81

0

0

0

0

0

0

0

0

0

0

0

81

80

1

0

0

0

4

4

0

0

4

0

11

11

0

0

11

0

0

0

2419

2419

0

482

1937

0

0

0

3662

3662

1603

1603

1603

0

2059

2059

0

12

1998

49

0

0

0

0

0

0

0

588

588

0

0

0

588

0

0

0

0

0

588

2

0

233

353

0

0

0

0

0

0

0

0

0

0

3

3

3

0

0

3

3

0

0

0

0

0

0

0

0

0

0

0

0

0

0

0

0

0

0

0

0

0

0

0

0

0

0

0

0

0

0

0

0

0

0

0

0

0

0

0

0

0

0

0

0

0

0

0

0

0

0

0

0

0

0

0
