# Supplementary material for: The microbiome biomarkers of pregnant women’s vaginal area predict preterm prelabor rupture in Western China
Source: Front Cell Infect Microbiol. 2024 Oct 31;14:1471027. doi: 10.3389/fcimb.2024.1471027 (PMC11560878; doi:10.3389/fcimb.2024.1471027)
Supplement: Supplementary file 1 [file DataSheet1.zip › compare_1/Community/KronaPlot/P36.krona.html]

Javascript must be enabled to view this page.

magnitude
magnitudeUnassigned

P36\_data\_for\_Krona

50717

50717

0

0

0

0

0

0

301

0

0

0

0

0

0

0

0

0

0

0

0

0

0

301

301

0

0

0

0

0

0

16

16

0

12

0

4

0

0

0

0

0

0

0

0

0

0

0

0

0

0

0

0

0

0

0

0

0

0

8

8

8

0

277

277

0

0

0

0

0

0

24

0

30

7

0

0

20

0

196

0

0

0

0

0

0

0

0

0

0

0

0

0

0

0

0

0

0

0

0

0

0

0

0

0

0

0

0

0

0

0

6

6

6

6

6

0

0

0

6

0

0

0

0

0

0

0

0

0

0

0

0

0

0

11

0

0

0

0

0

0

0

0

0

0

0

0

0

0

0

0

0

0

0

0

0

0

0

0

0

0

0

0

0

0

0

0

0

0

0

0

11

11

11

6

6

5

5

0

0

0

0

0

0

0

0

0

0

0

0

0

0

0

0

0

0

0

0

0

0

0

0

0

0

0

0

0

0

0

0

0

0

0

107

107

107

0

0

0

0

107

0

0

107

107

0

0

0

0

0

0

0

0

0

0

0

0

0

0

0

0

0

0

0

0

0

0

0

0

0

0

0

0

0

0

0

0

0

0

0

0

0

0

0

0

0

0

0

0

0

0

0

0

0

0

0

0

0

0

0

18

0

0

0

0

0

0

0

0

0

0

0

0

0

0

0

0

0

0

0

0

0

0

0

0

0

0

0

0

0

0

0

0

0

0

0

0

0

0

0

2

0

0

0

0

2

2

2

0

2

0

0

0

0

0

0

0

0

0

0

0

0

0

0

0

0

0

0

6

0

0

0

0

0

0

0

0

0

0

6

6

6

6

0

0

0

0

0

0

10

0

0

0

0

0

0

0

0

0

0

0

0

0

0

0

0

0

10

10

10

10

0

0

0

0

0

0

0

0

0

0

0

0

0

0

0

0

0

0

0

0

0

12

12

12

12

12

12

0

0

0

0

0

0

0

0

0

0

0

0

0

0

0

0

0

0

0

0

0

0

0

0

0

0

0

0

50262

182

182

0

0

0

0

0

0

0

0

0

0

0

0

74

0

0

0

0

7

0

7

0

0

0

0

67

67

0

0

0

0

0

0

0

0

0

0

0

0

0

0

0

16

16

16

92

92

70

22

0

0

0

0

49978

49978

14

14

14

0

49953

49953

0

0

49718

235

11

11

11

0

0

0

0

94

94

0

0

0

94

0

0

0

0

0

94

0

0

10

84

0

0

0

0

0

8

8

8

8

8

0

0

0

0

0

0

0

0

0

0

0

0

0

0

0

0

0

0

0

0

0

0

0

0

0

0

0

0

0

0

0

0

0

0

0

0

0

0

0

0

0

0

0

0

0

0

0

0

0

0

0

0

0

0

0

0

0

0

0

0

0

0
