# Supplementary material for: The microbiome biomarkers of pregnant women’s vaginal area predict preterm prelabor rupture in Western China
Source: Front Cell Infect Microbiol. 2024 Oct 31;14:1471027. doi: 10.3389/fcimb.2024.1471027 (PMC11560878; doi:10.3389/fcimb.2024.1471027)
Supplement: Supplementary file 1 [file DataSheet1.zip › compare_1/Community/KronaPlot/P9.krona.html]

Javascript must be enabled to view this page.

magnitude
magnitudeUnassigned

P9\_data\_for\_Krona

50717

50717

0

0

0

0

0

0

80

9

9

9

6

6

0

3

3

0

0

0

0

0

0

68

68

0

0

0

0

0

0

0

0

0

0

0

0

0

0

0

0

0

0

0

0

0

0

59

59

0

13

0

0

0

46

0

0

0

0

0

0

0

0

9

9

0

0

0

0

0

0

0

0

0

9

0

0

0

0

0

0

0

0

0

0

0

0

0

0

0

0

0

0

0

0

0

0

3

3

2

0

0

0

0

2

2

1

0

0

1

1

2

2

2

2

2

0

0

2

0

0

0

0

0

0

0

0

0

0

0

0

0

0

0

20

20

20

20

20

18

2

0

0

0

0

0

0

0

0

0

0

0

0

0

0

0

0

0

0

0

0

0

0

0

0

0

0

0

0

0

0

0

0

0

0

0

0

0

0

0

0

0

0

0

0

0

0

0

0

0

0

0

0

0

0

0

0

0

0

0

0

0

0

0

0

0

0

0

0

0

0

0

0

21

21

0

0

0

0

0

0

0

0

0

0

0

0

0

0

0

0

0

0

0

0

0

0

0

15

15

15

15

0

0

0

0

0

0

0

0

0

0

0

6

6

0

0

6

6

0

0

0

0

0

0

0

0

0

0

0

0

0

0

0

0

0

0

0

0

0

33

4

0

0

0

0

0

0

0

0

0

0

0

0

0

0

0

0

0

0

0

0

0

2

2

2

2

0

0

0

0

0

2

2

2

2

0

0

0

0

0

0

0

0

0

0

0

0

0

0

0

0

0

0

0

0

0

0

0

0

0

0

0

0

0

0

0

0

13

13

0

0

0

13

13

13

0

0

0

0

0

0

0

0

0

0

0

0

0

16

0

0

0

0

0

0

0

0

0

0

0

0

0

10

10

10

10

0

0

0

0

0

0

0

0

6

6

6

0

6

0

0

0

0

0

0

0

0

0

0

0

0

0

0

0

0

0

0

5

0

0

0

0

0

3

3

3

3

0

3

2

2

2

0

0

2

0

0

0

2

0

0

0

0

0

0

50552

117

117

7

0

0

0

0

0

0

0

0

0

7

7

101

7

7

0

0

20

20

0

0

0

0

0

74

74

0

6

6

6

0

0

0

0

0

0

0

0

0

0

0

1

1

1

2

2

0

0

2

0

0

0

50431

50431

9

9

9

0

50422

50422

0

26

49954

442

0

0

0

0

0

0

0

4

4

0

0

0

4

0

0

0

0

0

4

0

0

4

0

0

0

0

0

0

0

0

0

0

0

0

0

0

0

0

0

0

2

0

0

0

0

0

0

0

0

0

0

2

2

2

2

2

0

0

0

0

0

0

0

0

0

0

0

0

0

0

0

0

0

0

0

2

2

2

2

2

0

0

0

0

0

0

2

0

0

0

0

0

0

0

0
