# Supplementary figures and images for: The microbiome biomarkers of pregnant women’s vaginal area predict preterm prelabor rupture in Western China
Source: Front Cell Infect Microbiol. 2024 Oct 31;14:1471027. doi: 10.3389/fcimb.2024.1471027 (PMC11560878; doi:10.3389/fcimb.2024.1471027)

Rank–abundance distribution curve

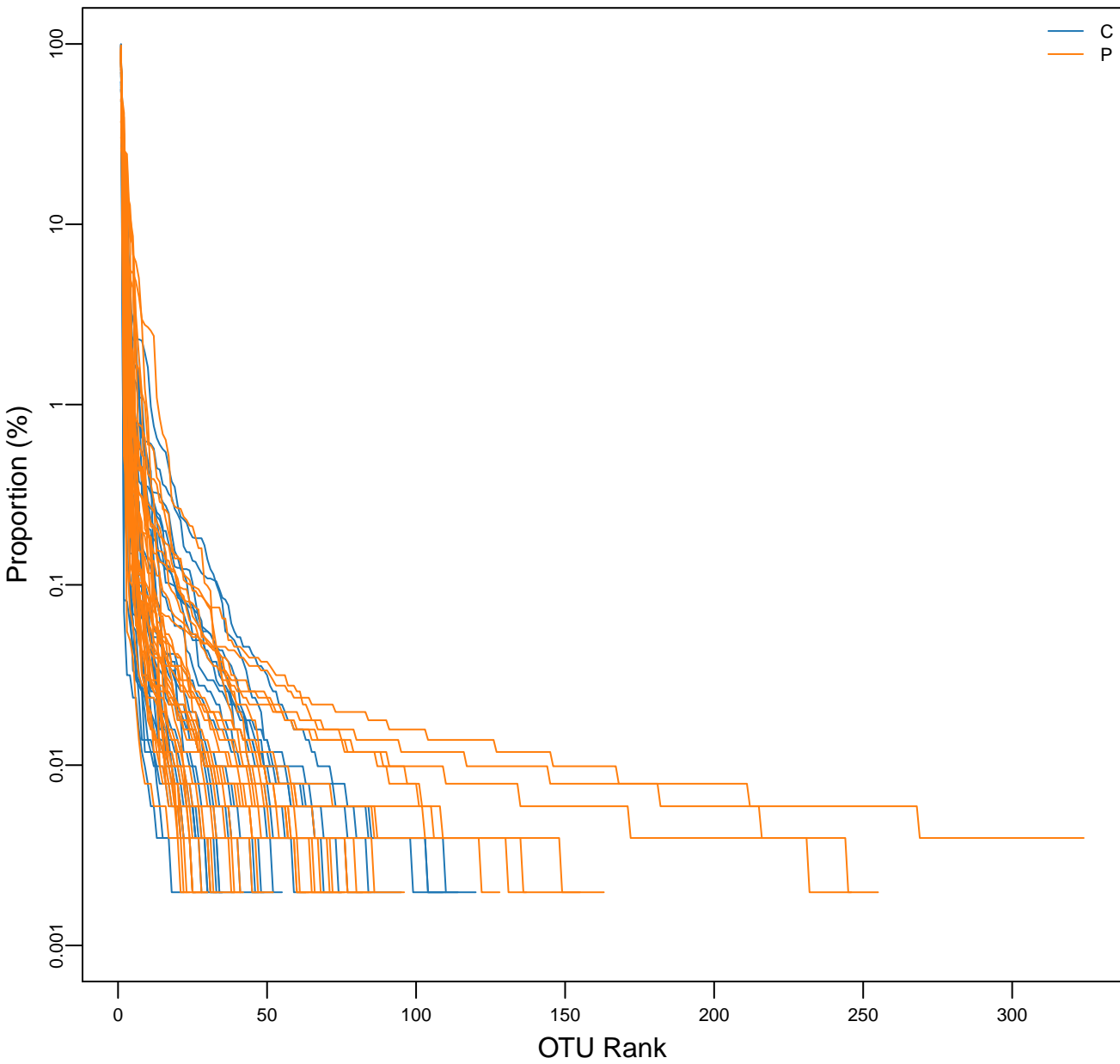

Supplement: Supplementary file 1 [file DataSheet1.zip › compare_1/AlphaDiversity/RankAbundance/Rank.Abundance.pdf]

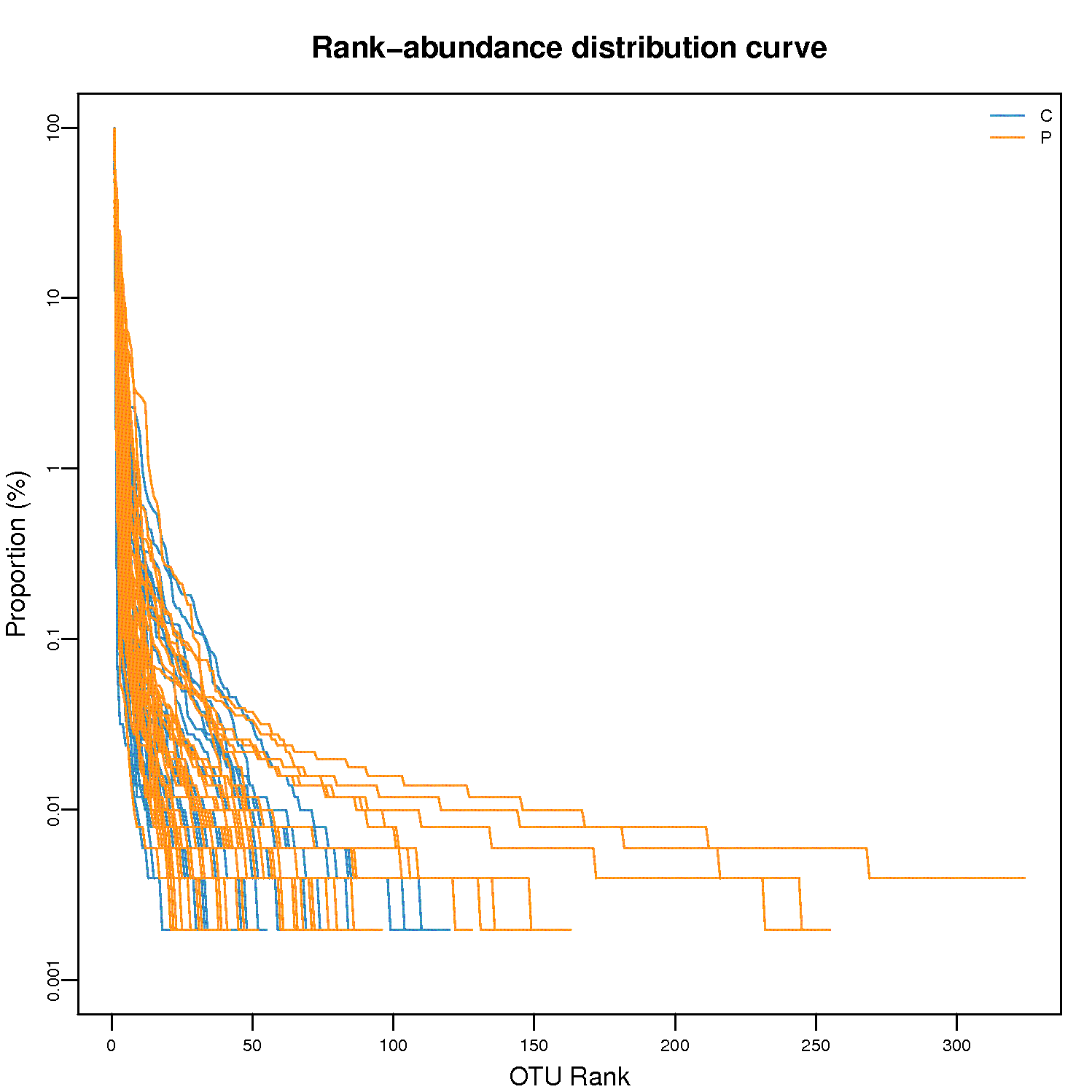

Supplement: Supplementary file 1 [file DataSheet1.zip › compare_1/AlphaDiversity/RankAbundance/Rank.Abundance.pdf.png]

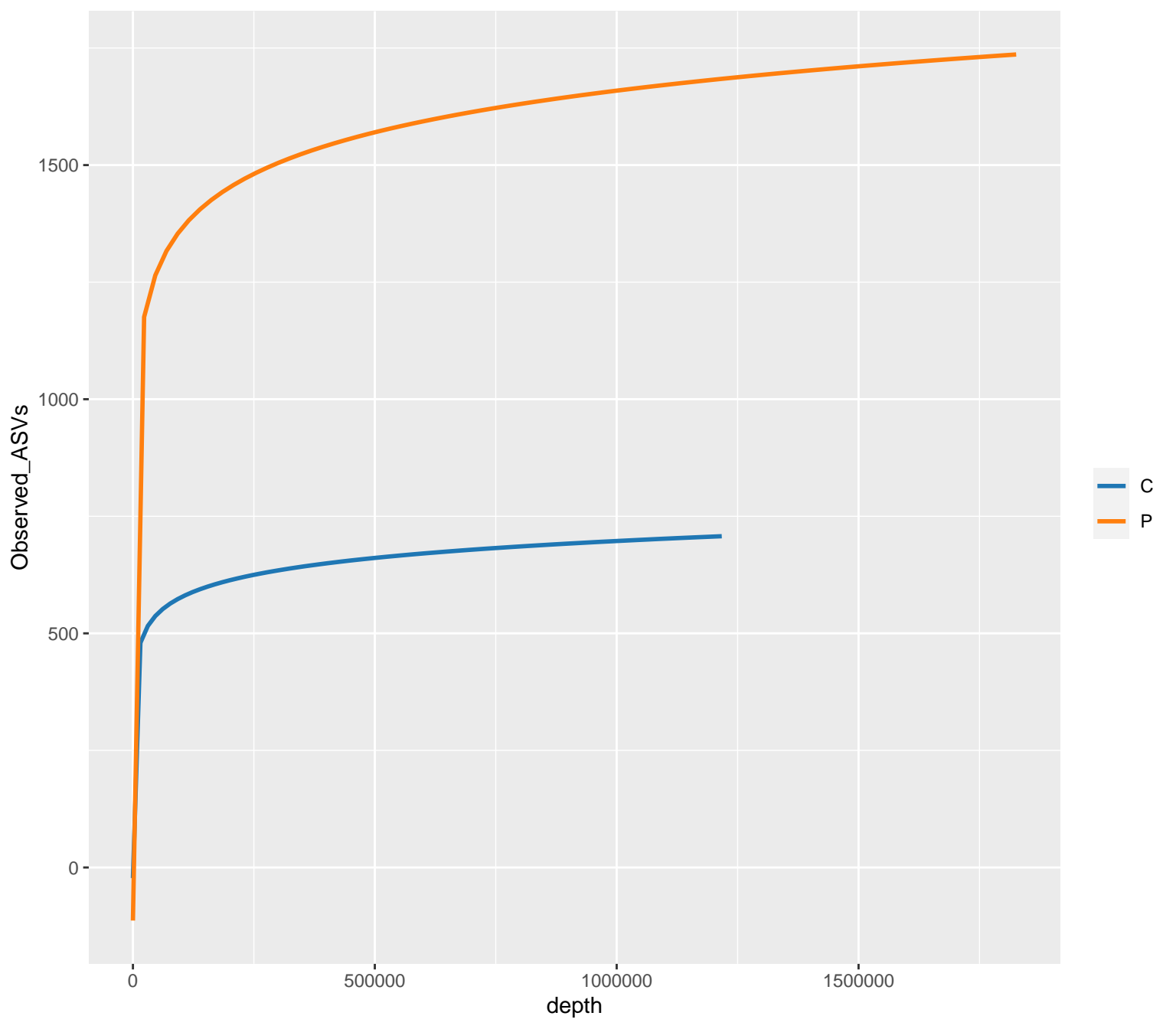

Supplement: Supplementary file 1 [file DataSheet1.zip › compare_1/AlphaDiversity/Rarefaction/group.rarefaction.pdf]

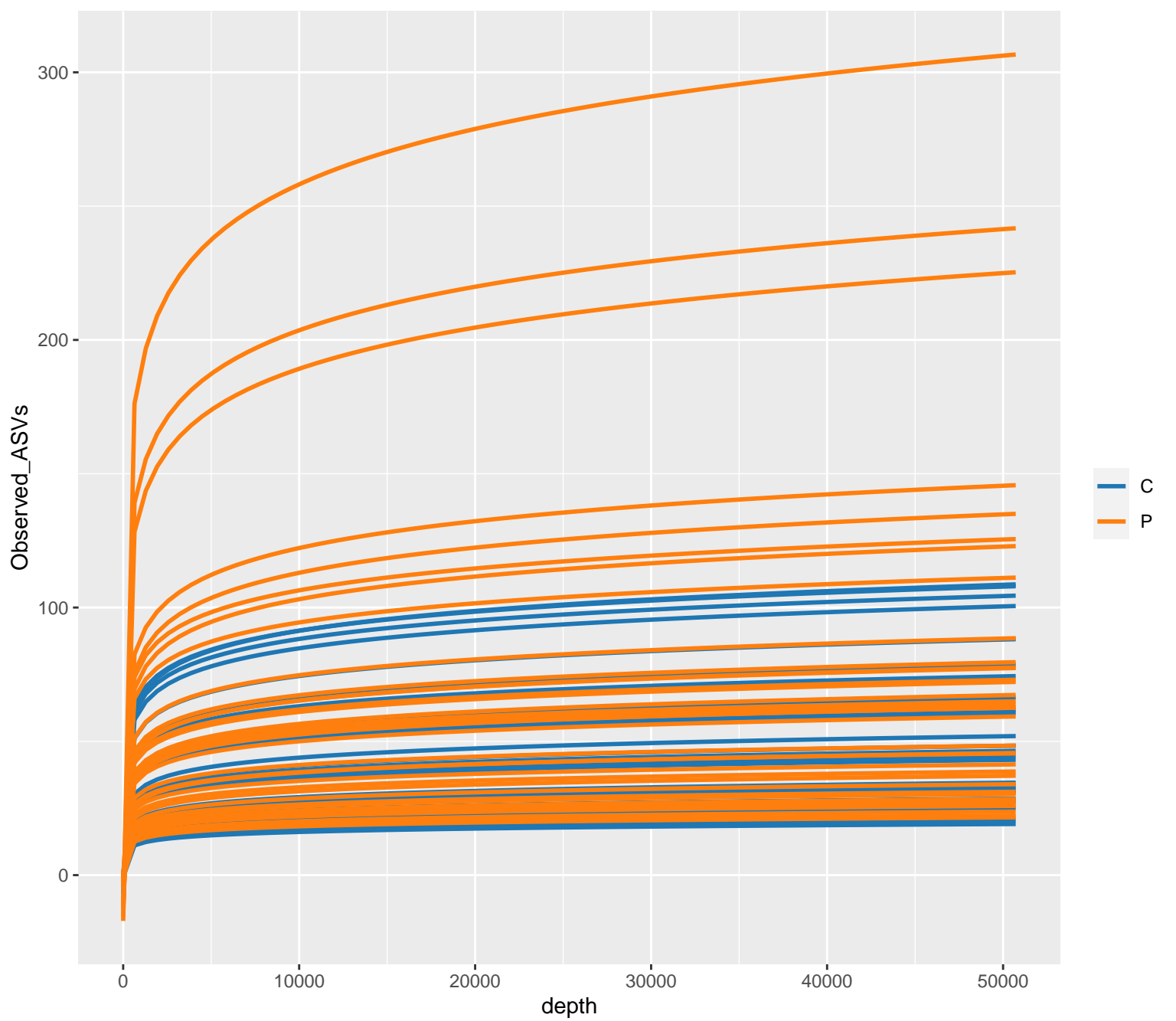

Supplement: Supplementary file 1 [file DataSheet1.zip › compare_1/AlphaDiversity/Rarefaction/sample.rarefaction.pdf]

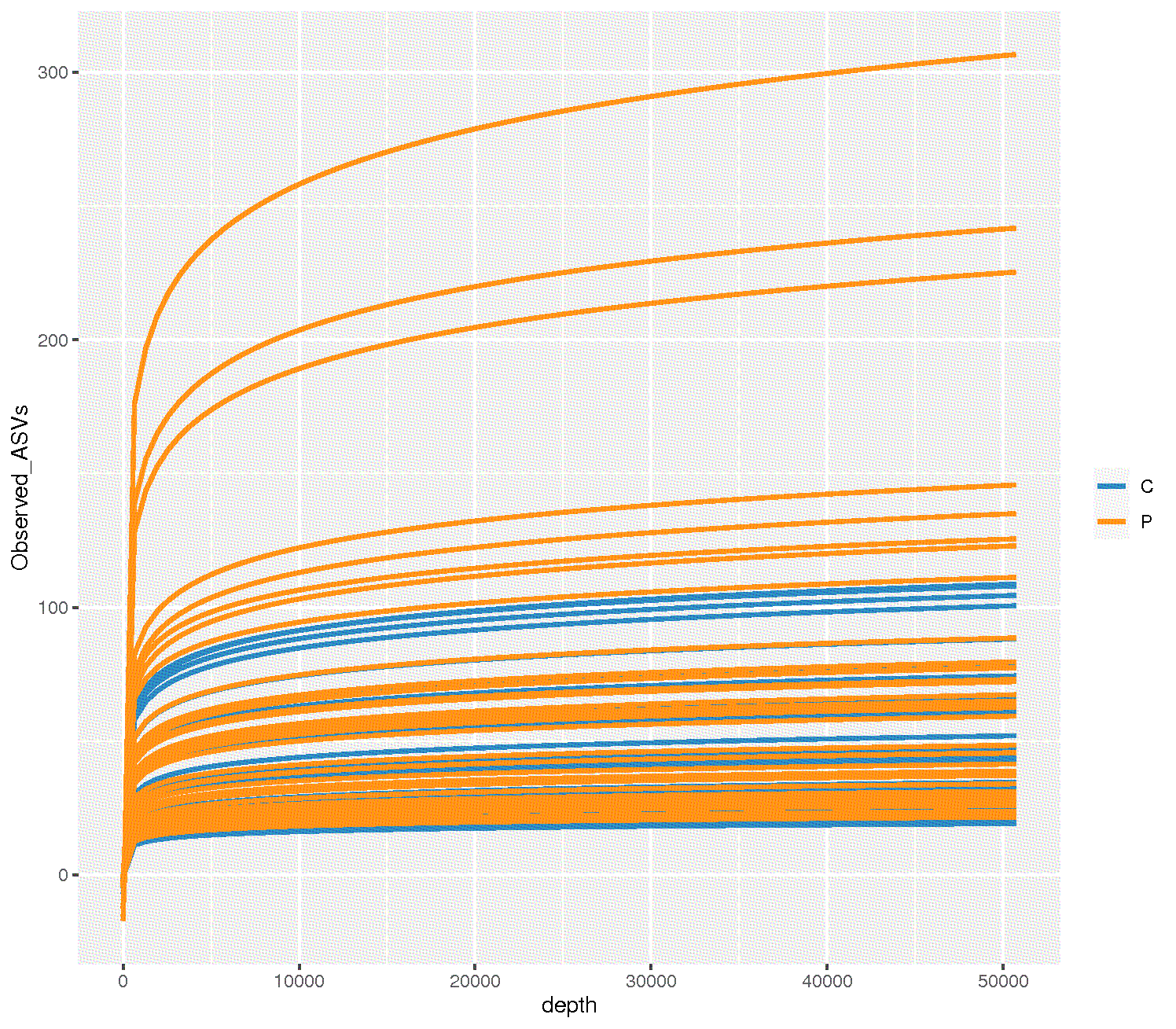

Supplement: Supplementary file 1 [file DataSheet1.zip › compare_1/AlphaDiversity/Rarefaction/sample.rarefaction.pdf.png]

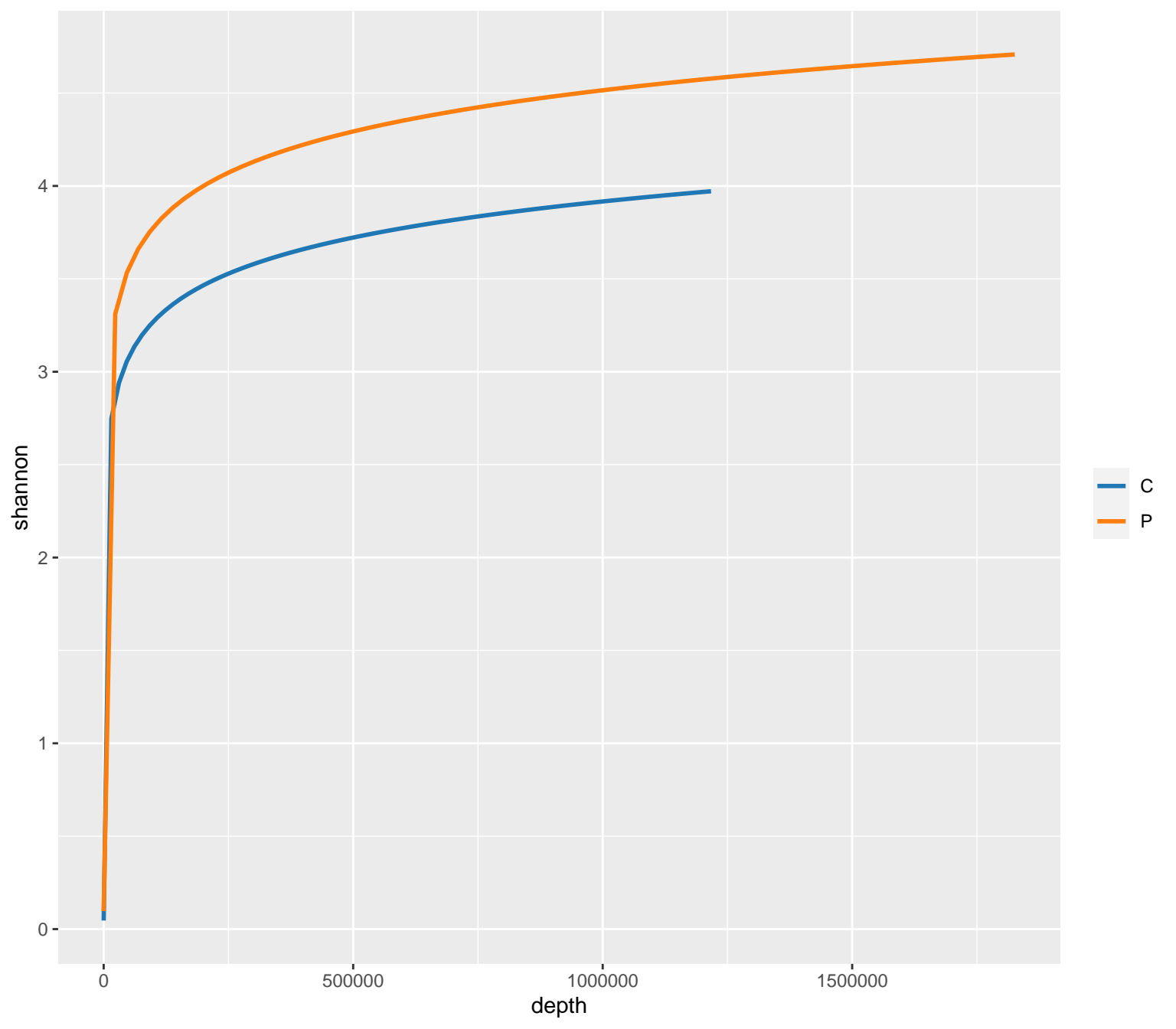

Supplement: Supplementary file 1 [file DataSheet1.zip › compare_1/AlphaDiversity/Shannon/group.shannon.pdf]

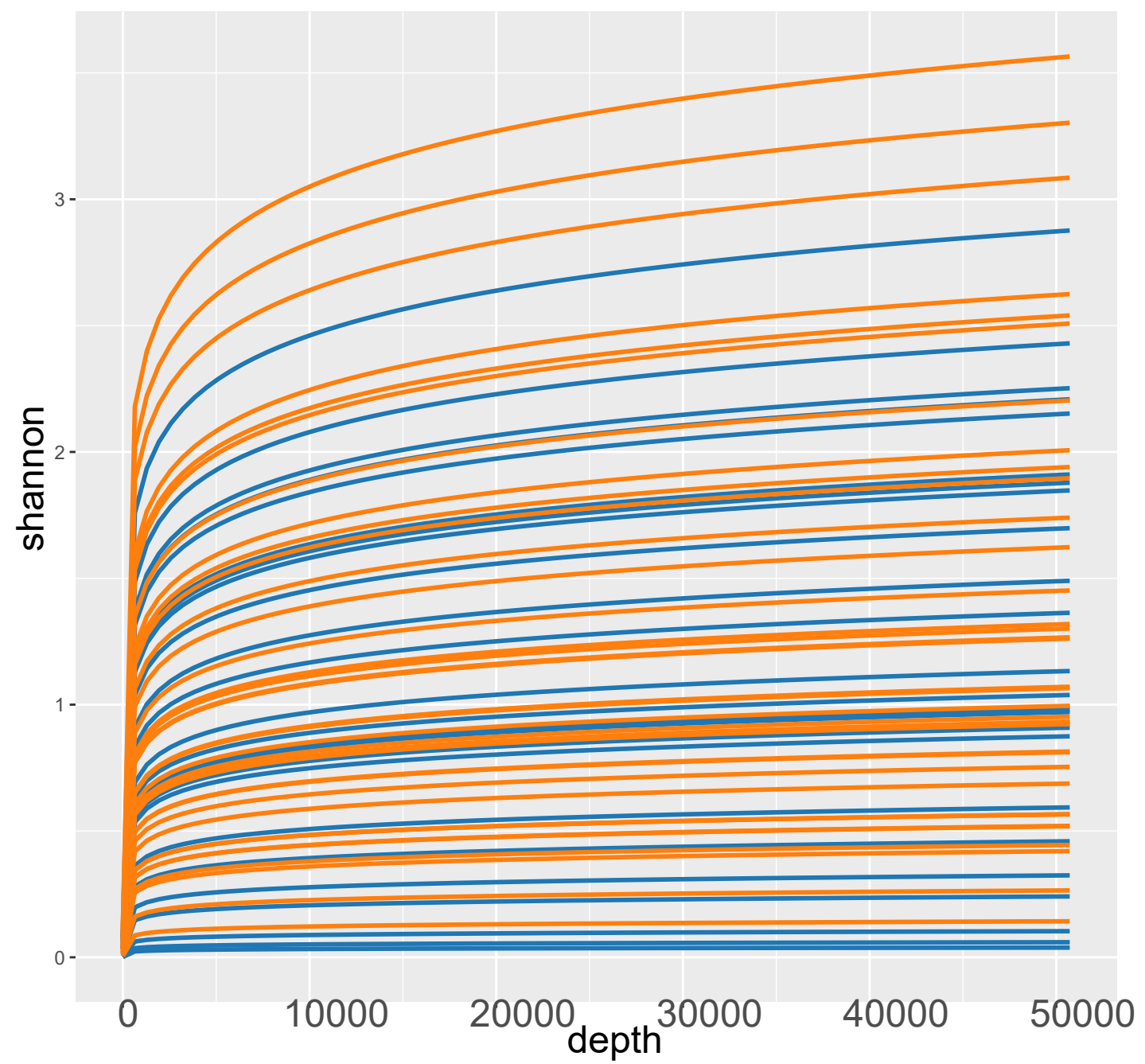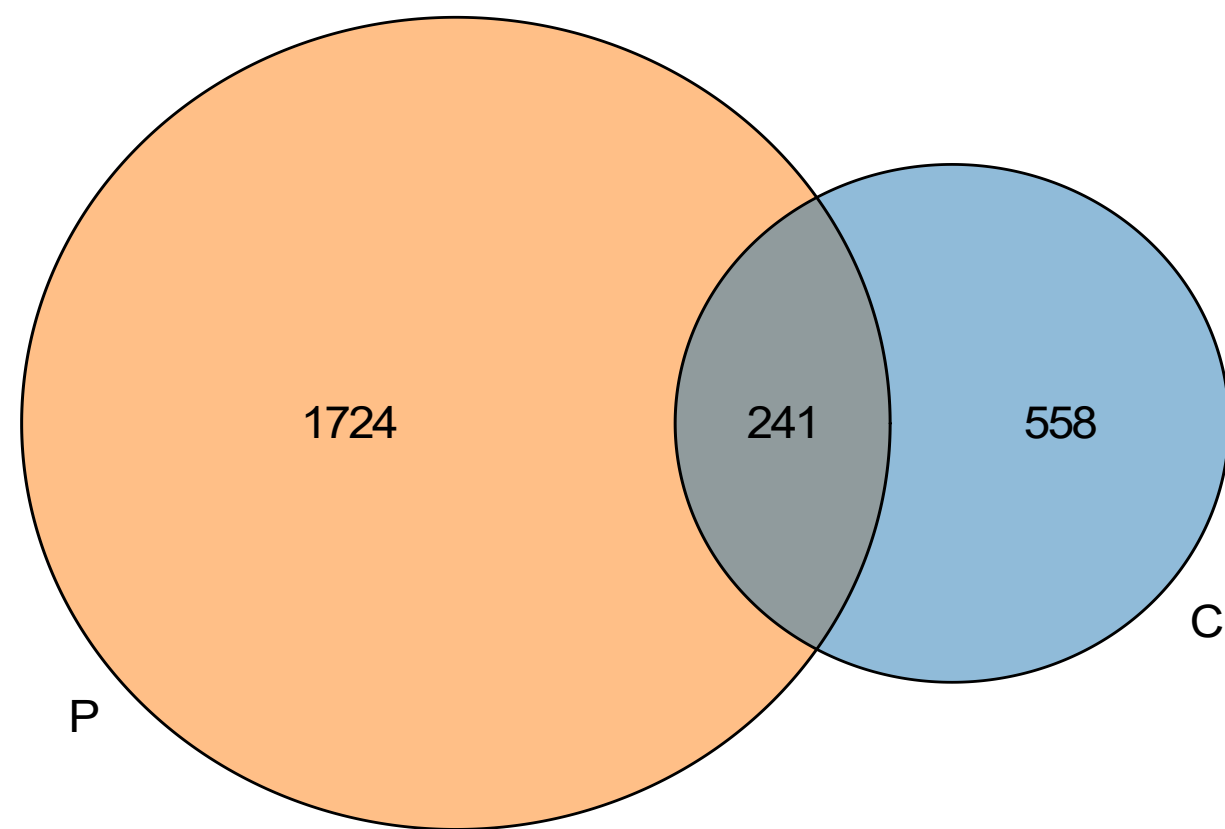

Supplement: Supplementary file 1 [file DataSheet1.zip › compare_1/AlphaDiversity/Shannon/sample.shannon.pdf]

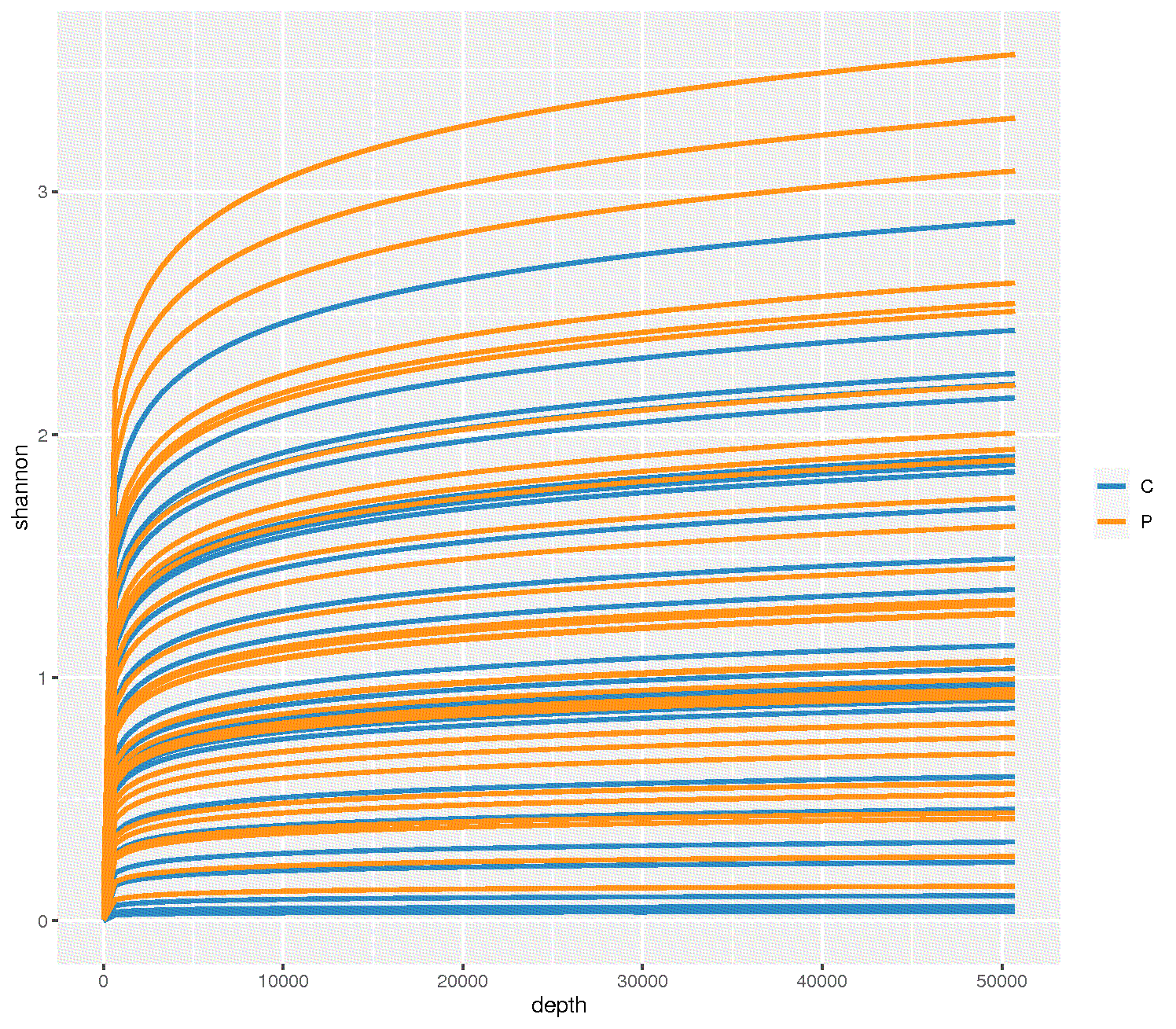

Supplement: Supplementary file 1 [file DataSheet1.zip › compare_1/AlphaDiversity/Shannon/sample.shannon.pdf.png]

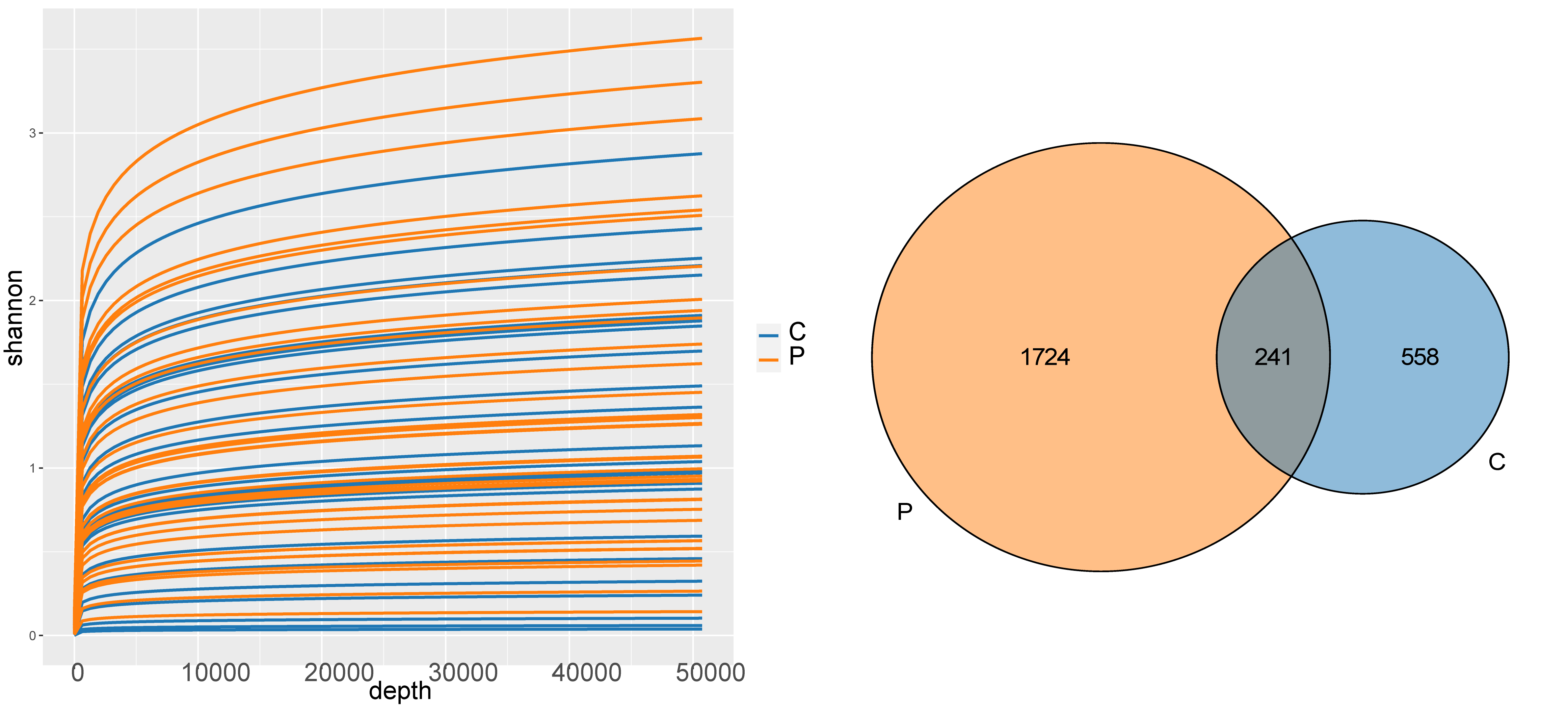

Supplement: Supplementary file 1 [file DataSheet1.zip › compare_1/AlphaDiversity/Shannon/sample.shannon.tif]

species accumulation curve

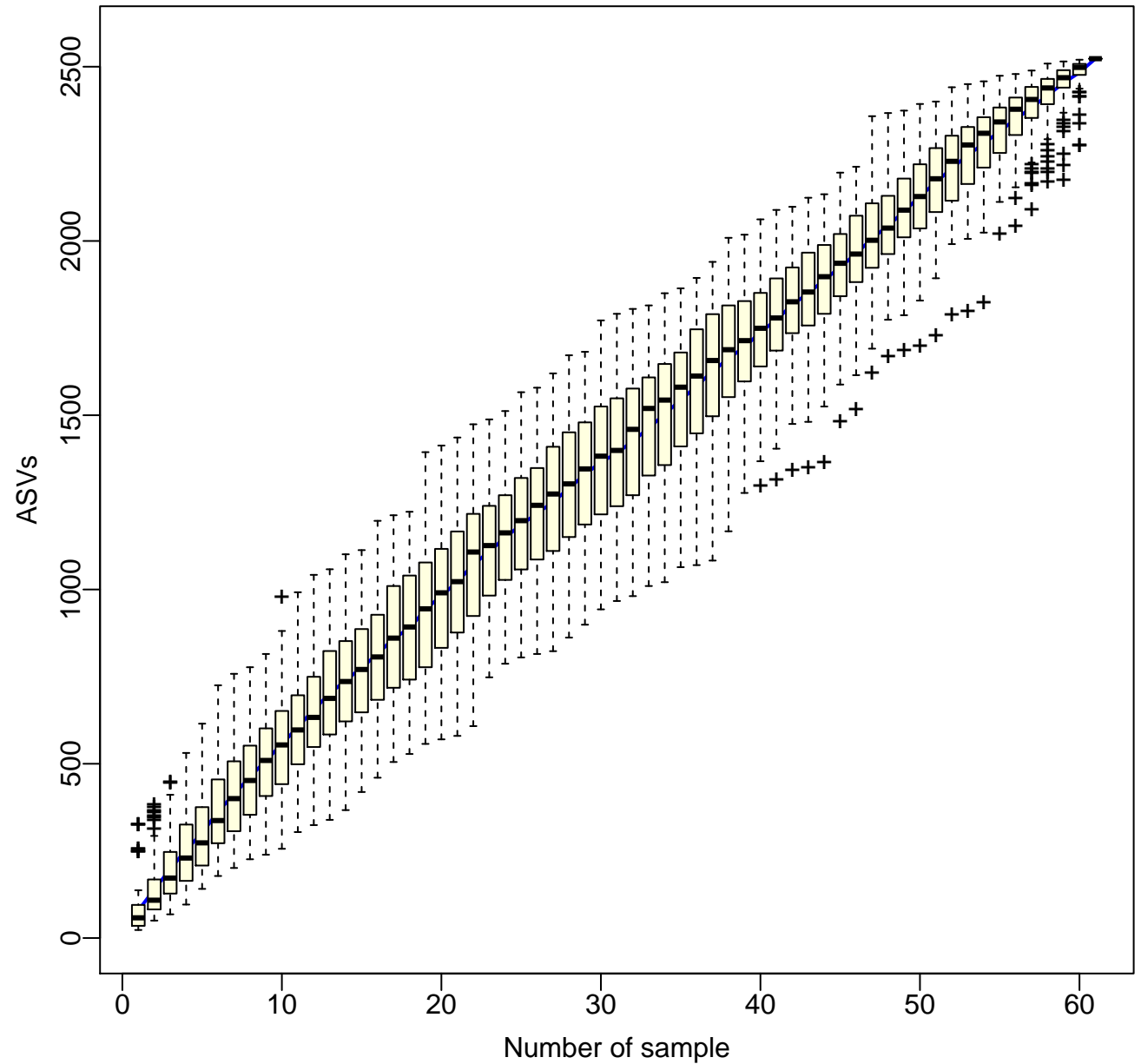

Supplement: Supplementary file 1 [file DataSheet1.zip › compare_1/AlphaDiversity/Specaccum/all.sample.specaccum.pdf]

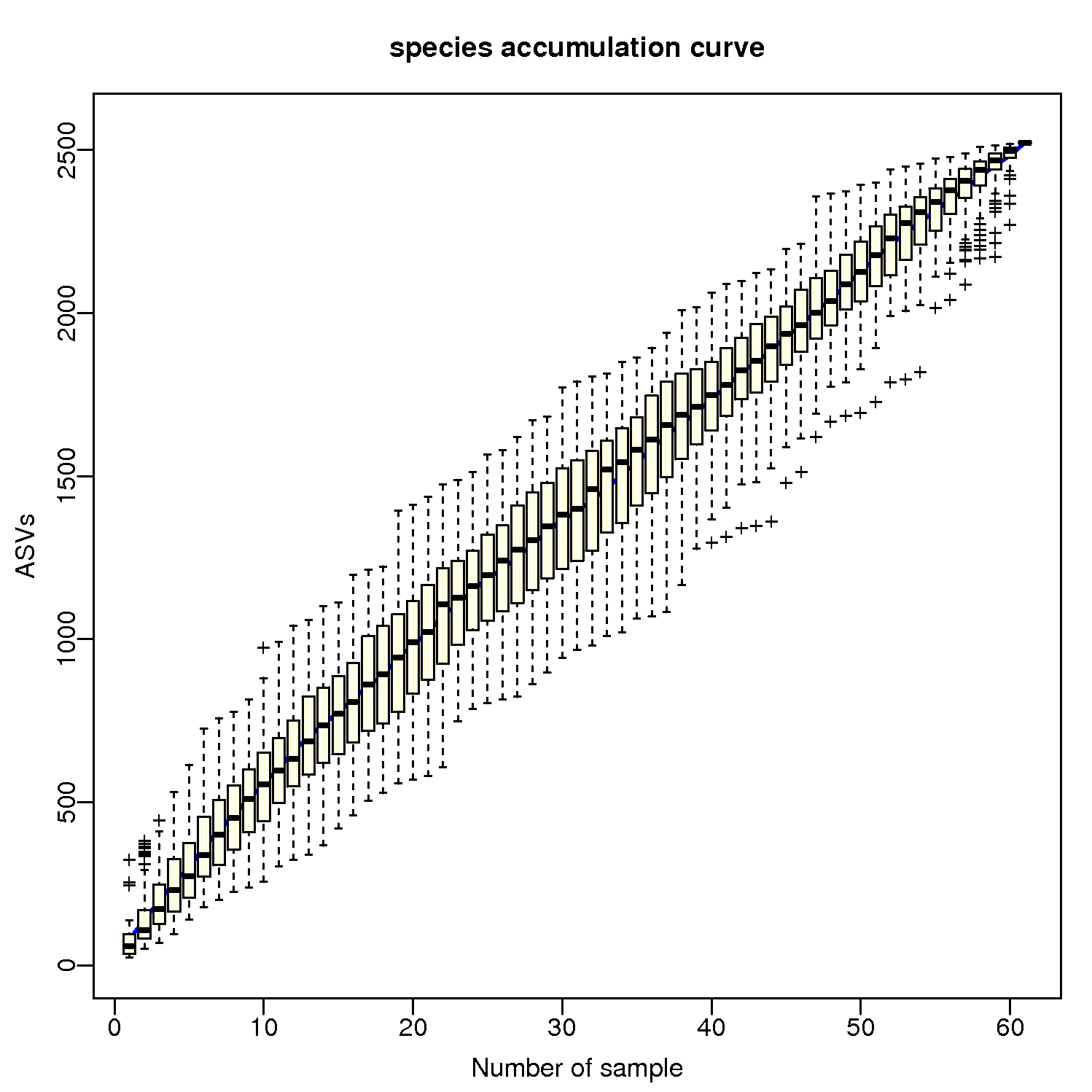

Supplement: Supplementary file 1 [file DataSheet1.zip › compare_1/AlphaDiversity/Specaccum/all.sample.specaccum.pdf.png]

species accumulation curve

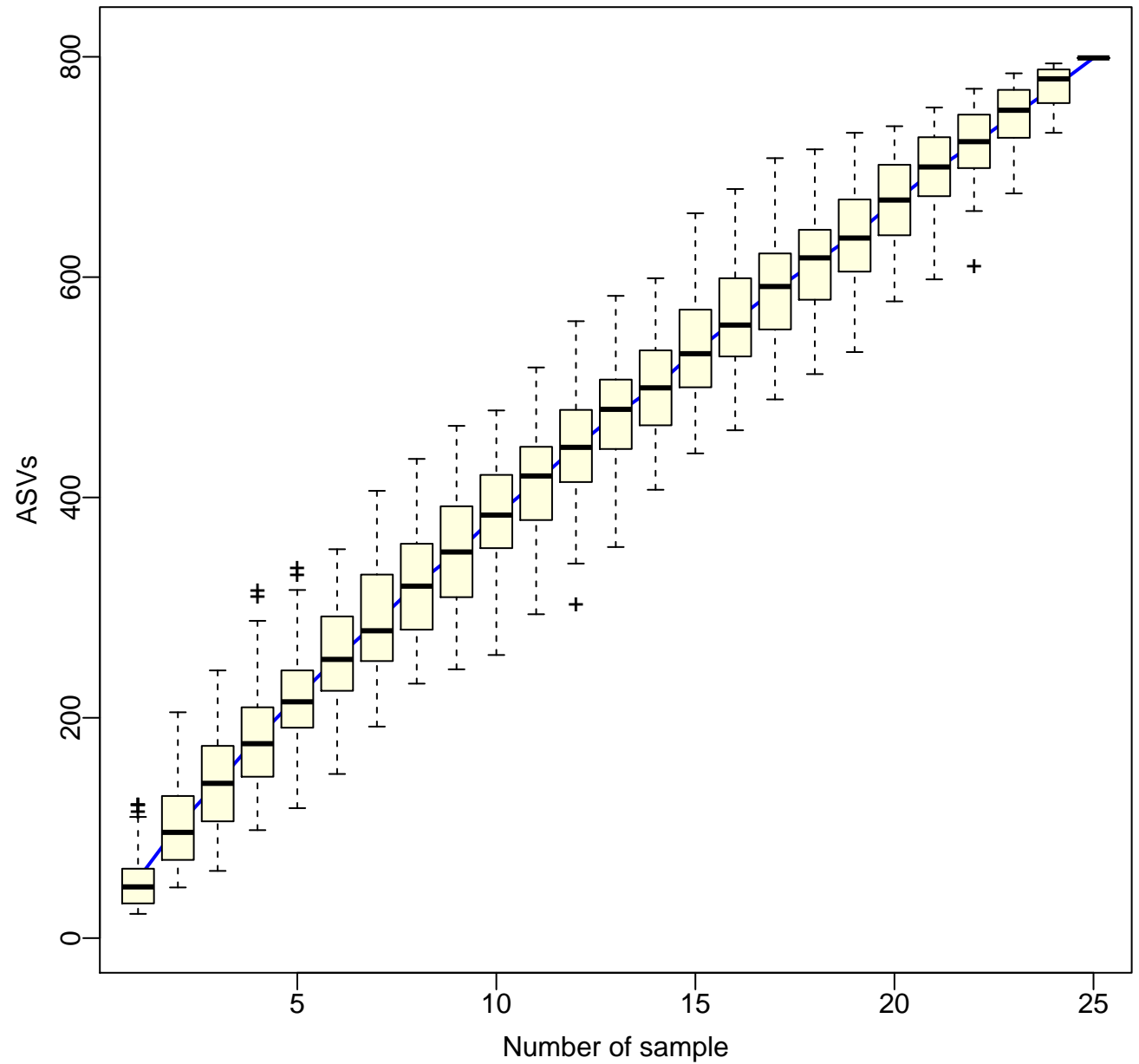

Supplement: Supplementary file 1 [file DataSheet1.zip › compare_1/AlphaDiversity/Specaccum/C.specaccum.pdf]

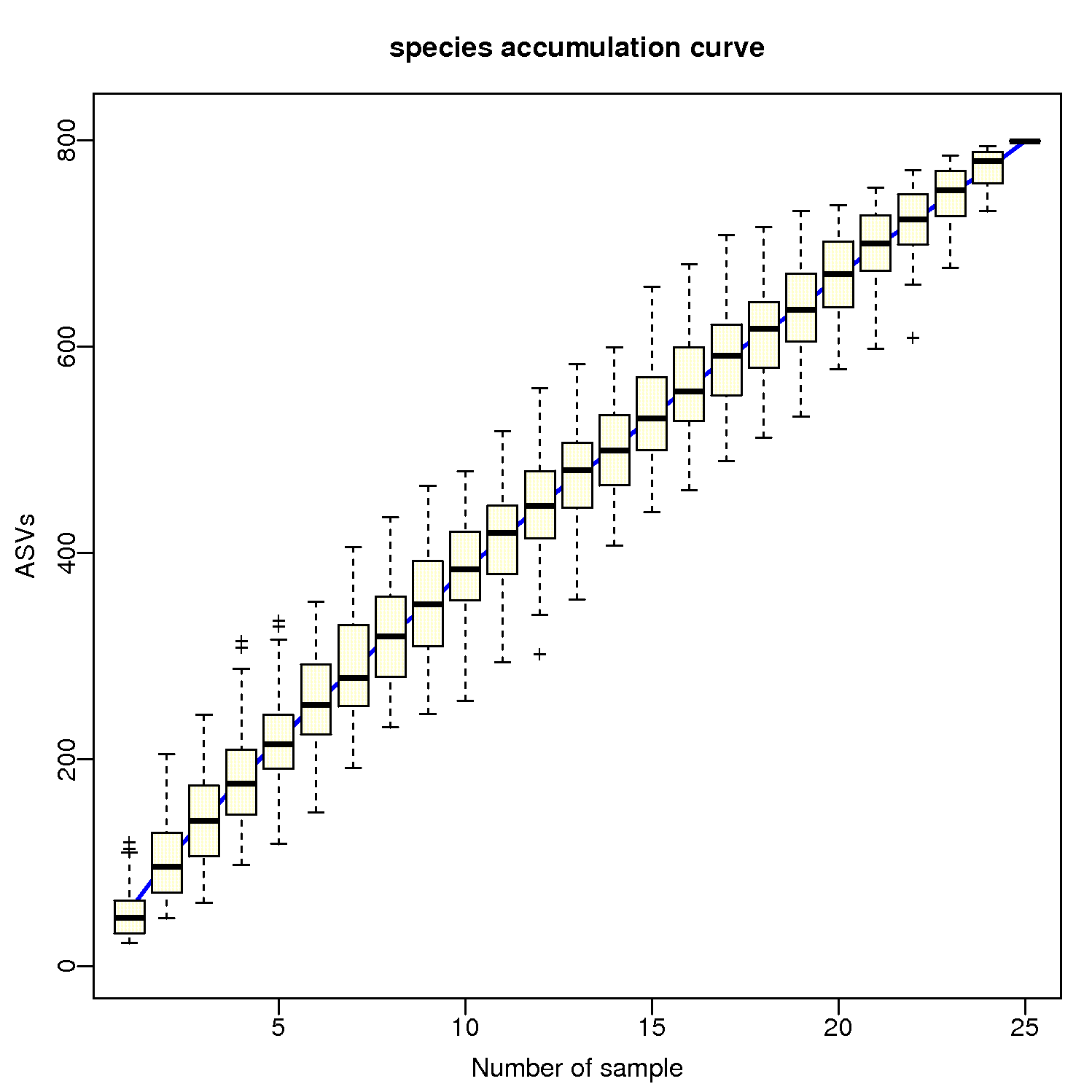

Supplement: Supplementary file 1 [file DataSheet1.zip › compare_1/AlphaDiversity/Specaccum/C.specaccum.pdf.png]

species accumulation curve

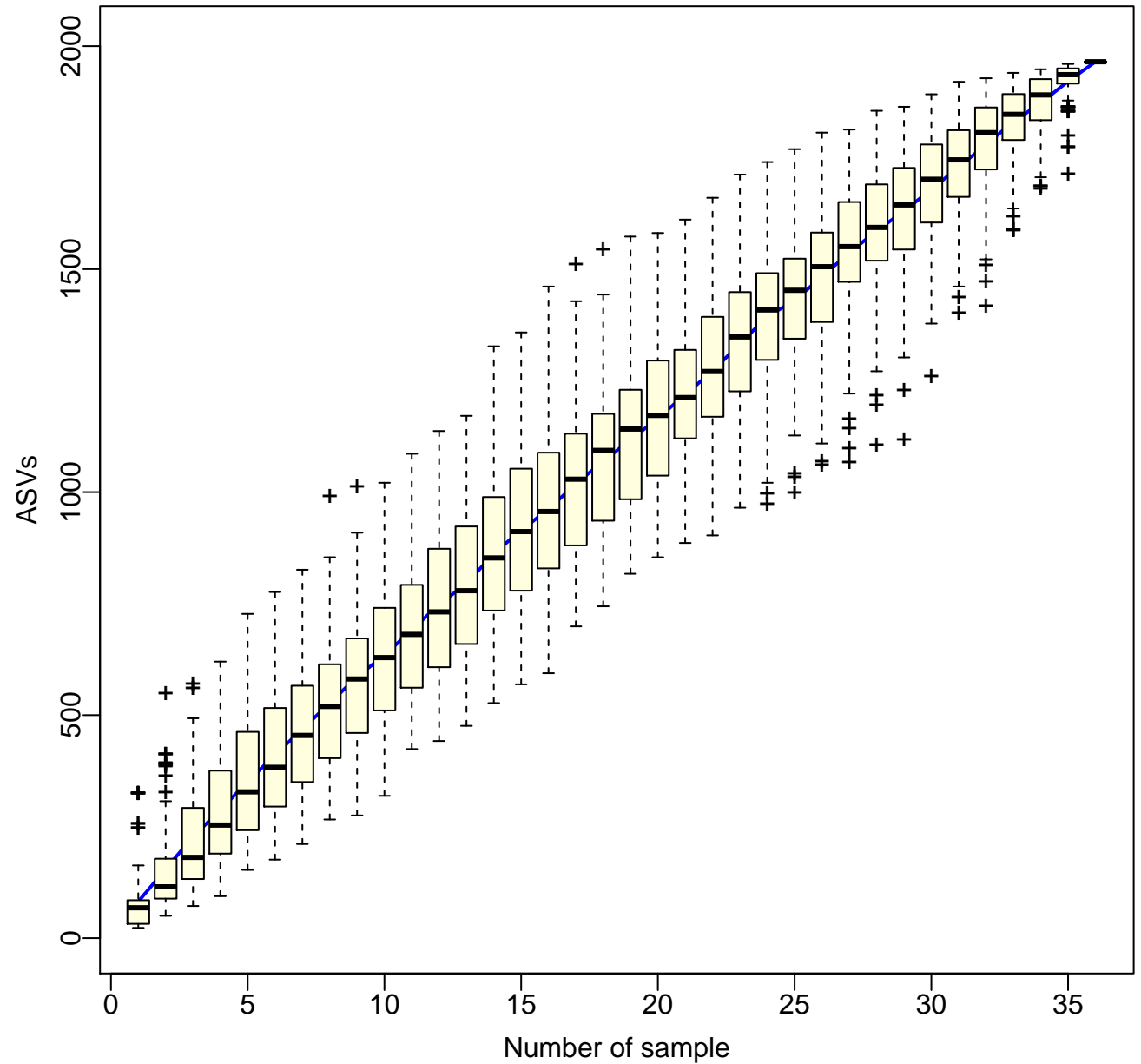

Supplement: Supplementary file 1 [file DataSheet1.zip › compare_1/AlphaDiversity/Specaccum/P.specaccum.pdf]

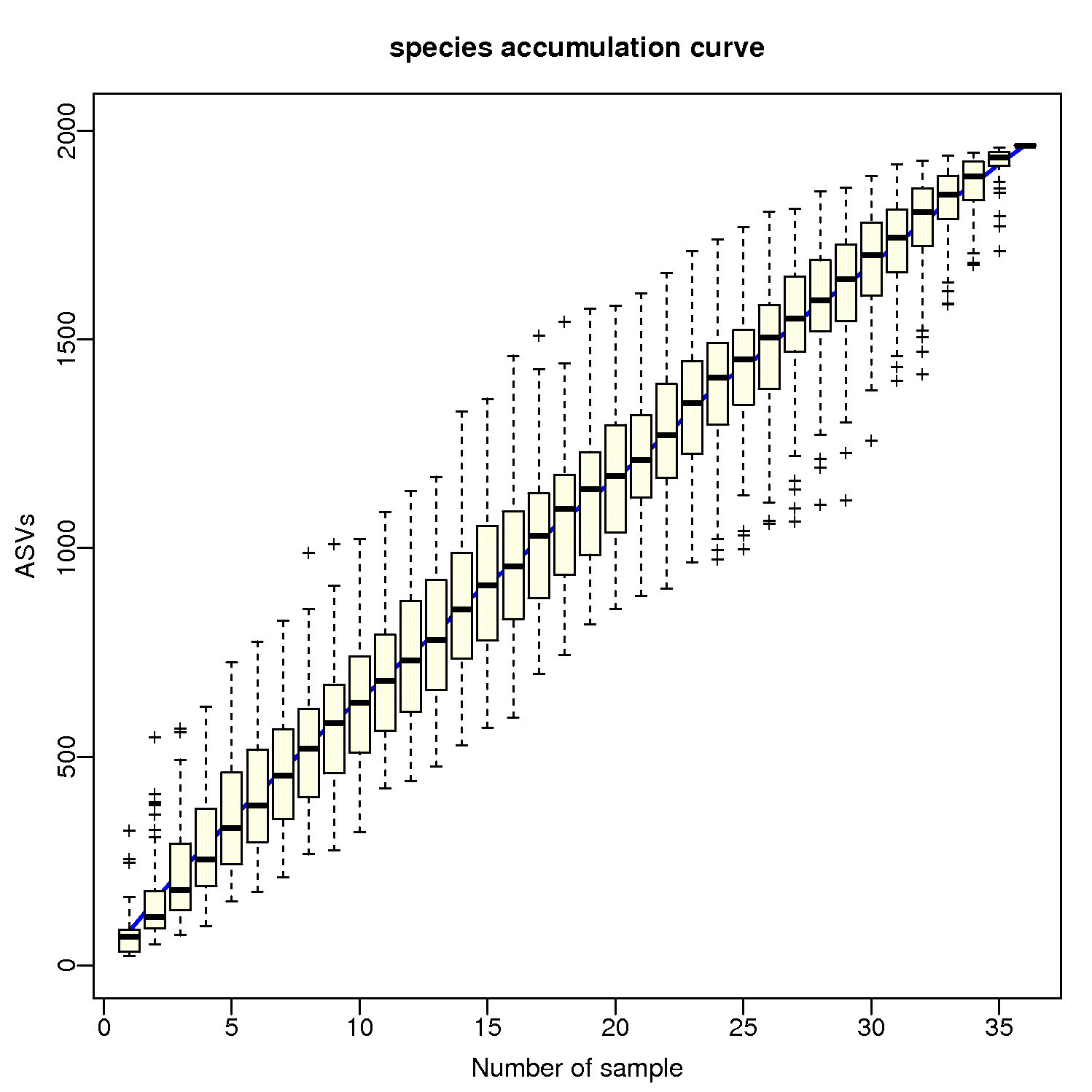

Supplement: Supplementary file 1 [file DataSheet1.zip › compare_1/AlphaDiversity/Specaccum/P.specaccum.pdf.png]

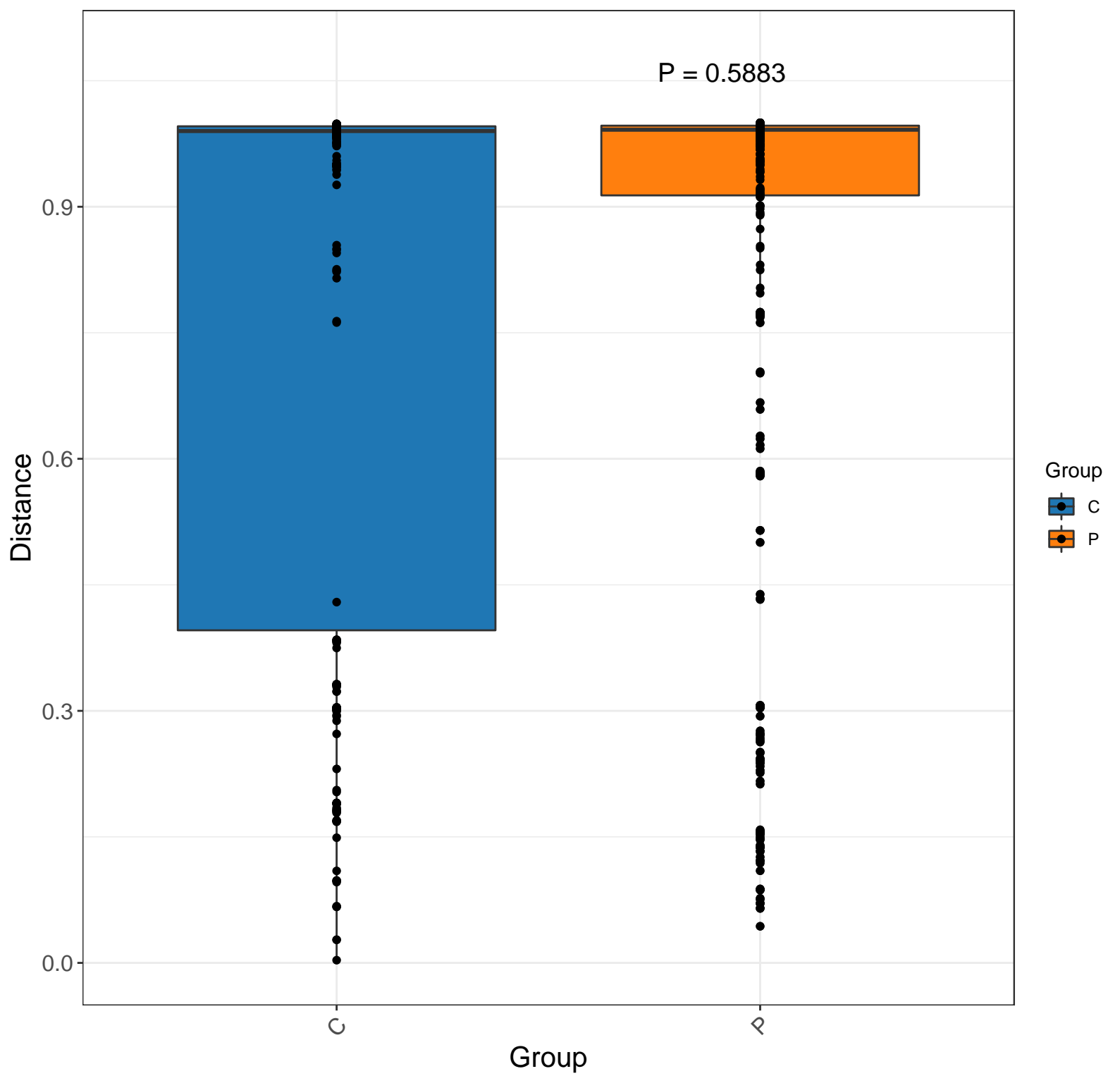

Supplement: Supplementary file 1 [file DataSheet1.zip › compare_1/BetaDiversity/ADONIS/All_group/bray.Dist.Boxplot.pdf]

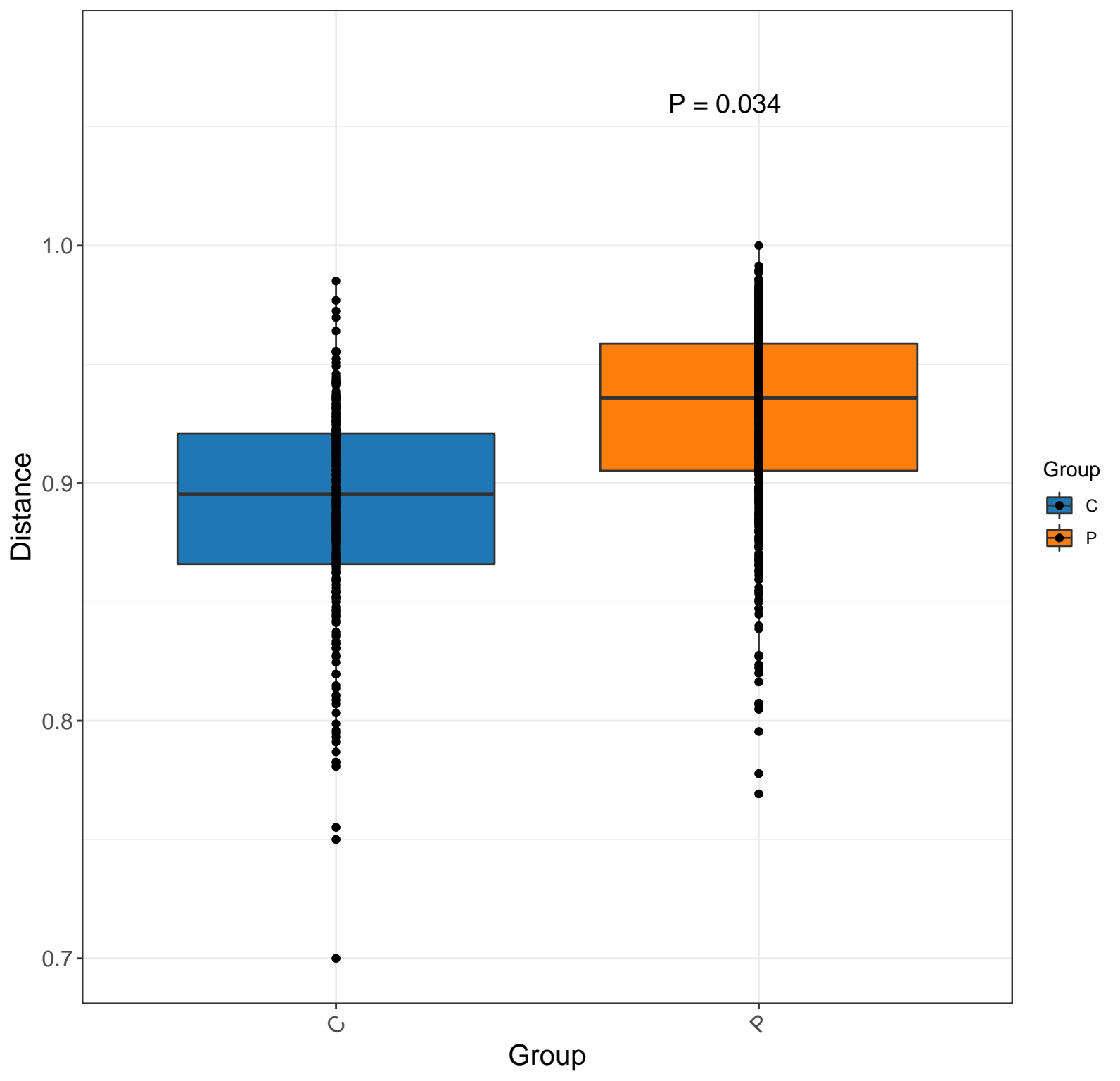

Supplement: Supplementary file 1 [file DataSheet1.zip › compare_1/BetaDiversity/ADONIS/All_group/jaccard.Dist.Boxplot.pdf]

# NMDS

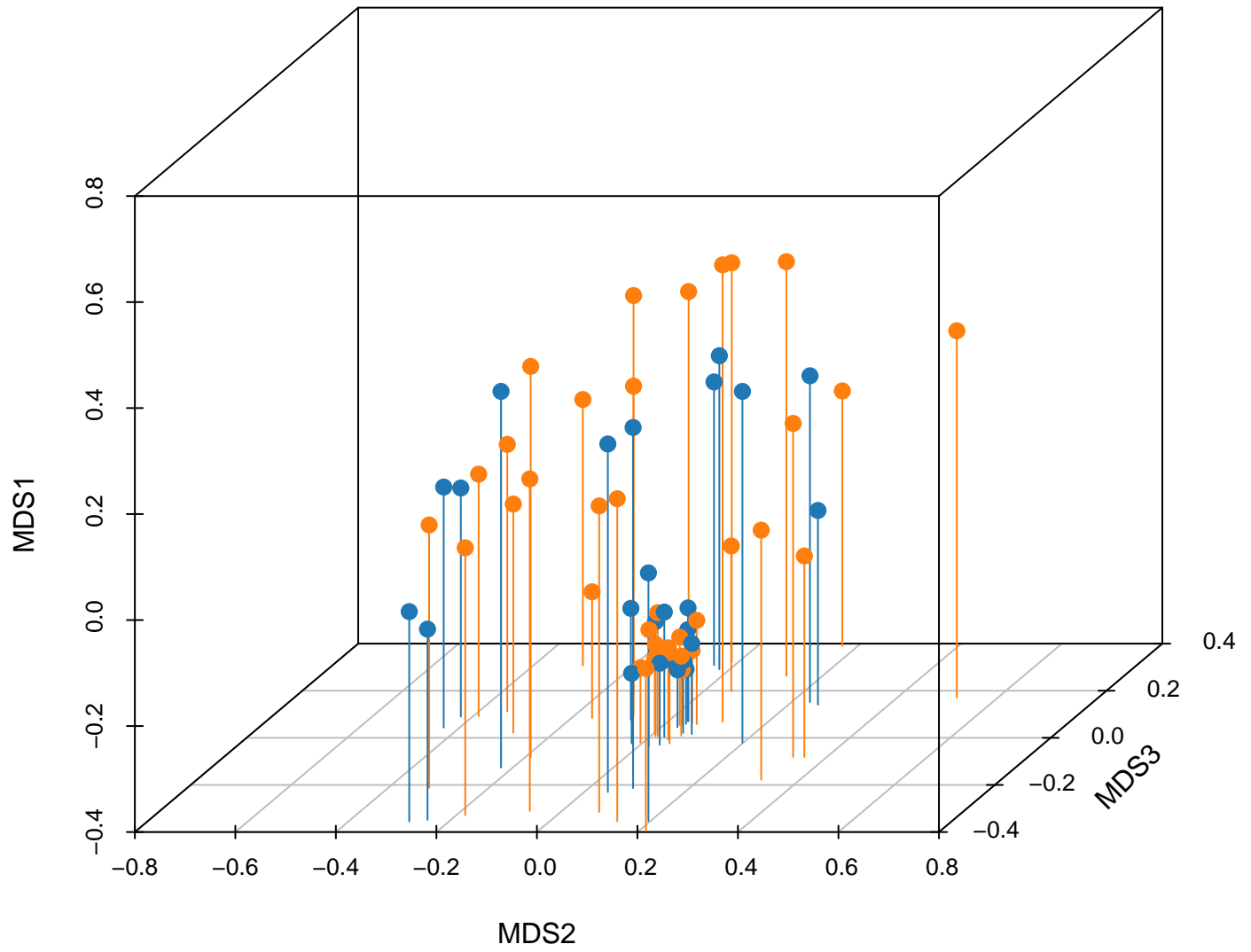

● C ● P

Supplement: Supplementary file 1 [file DataSheet1.zip › compare_1/BetaDiversity/NMDS/All_group/bray.3D.NMDS.pdf]

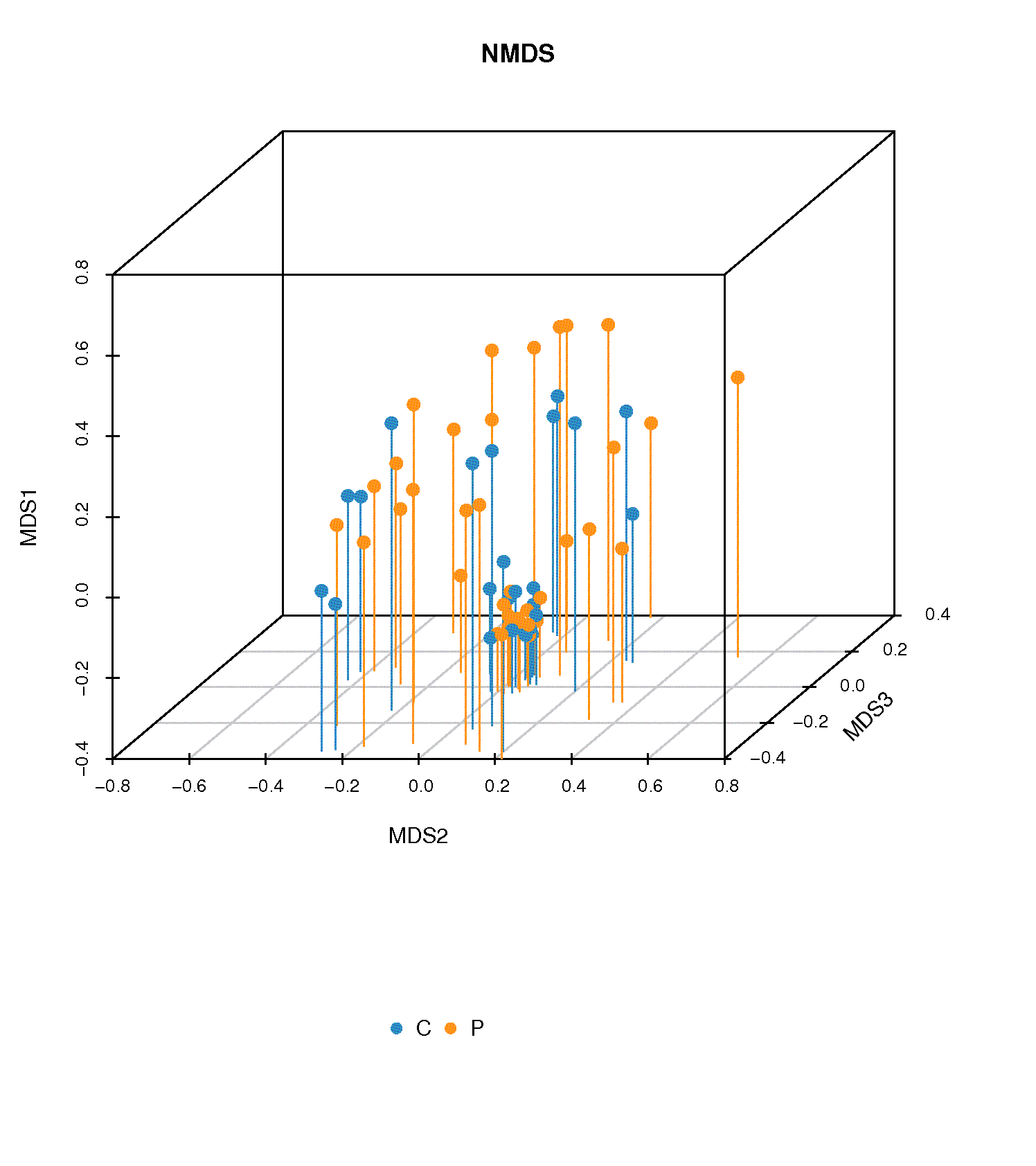

Supplement: Supplementary file 1 [file DataSheet1.zip › compare_1/BetaDiversity/NMDS/All_group/bray.3D.NMDS.pdf.png]

# NMDS

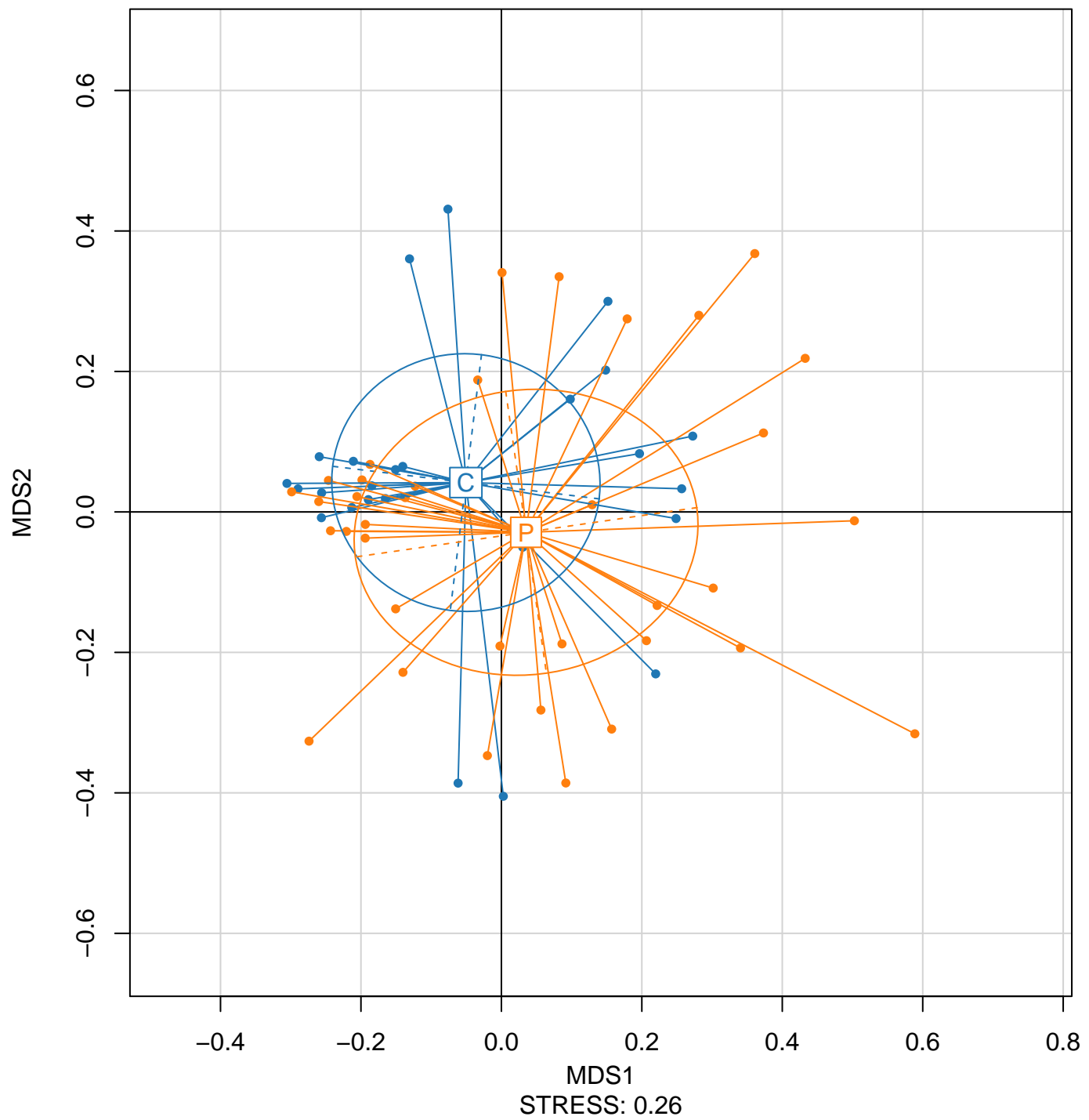

Supplement: Supplementary file 1 [file DataSheet1.zip › compare_1/BetaDiversity/NMDS/All_group/bray.NMDS.pdf]

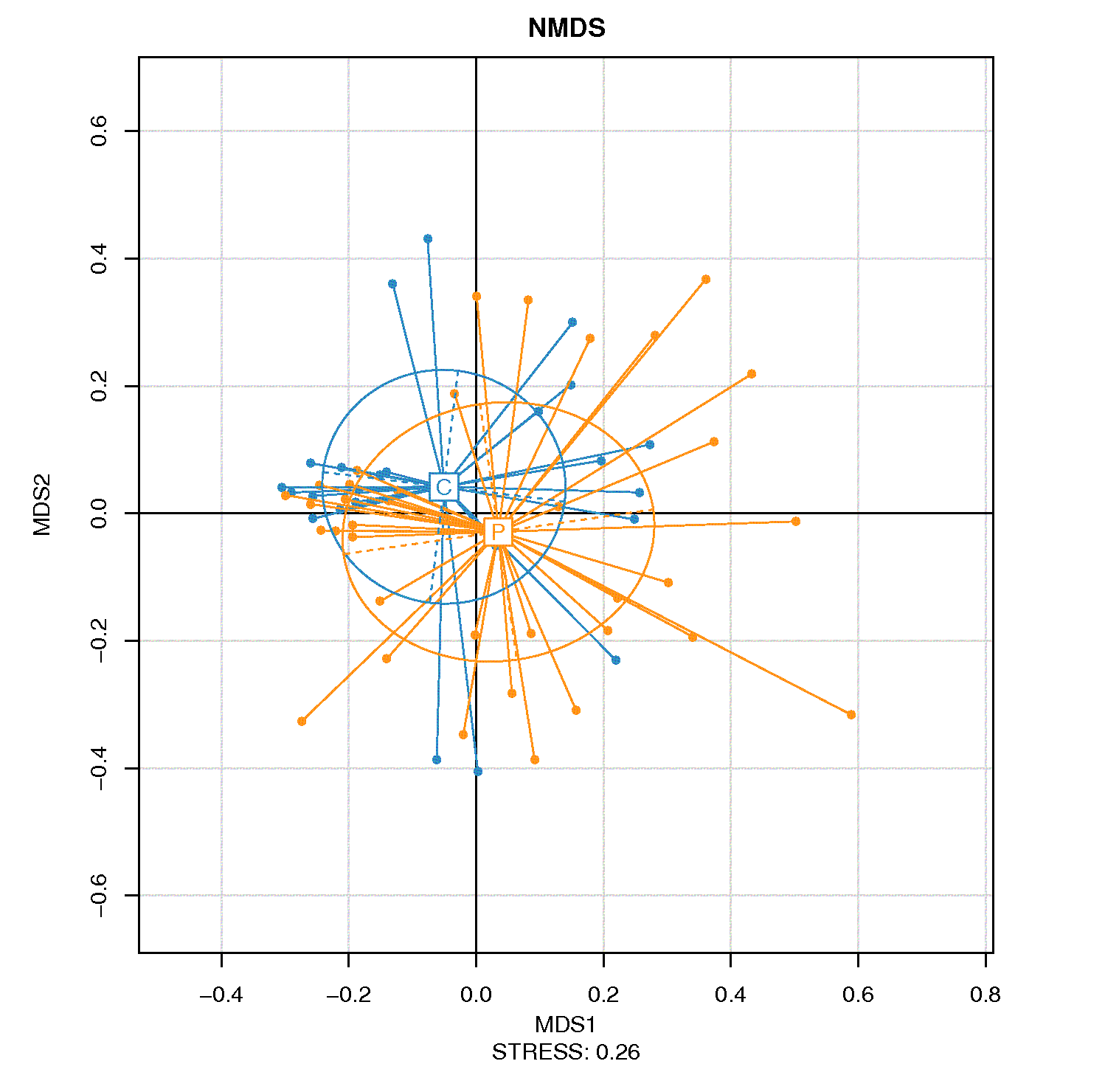

Supplement: Supplementary file 1 [file DataSheet1.zip › compare_1/BetaDiversity/NMDS/All_group/bray.NMDS.pdf.png]

# NMDS

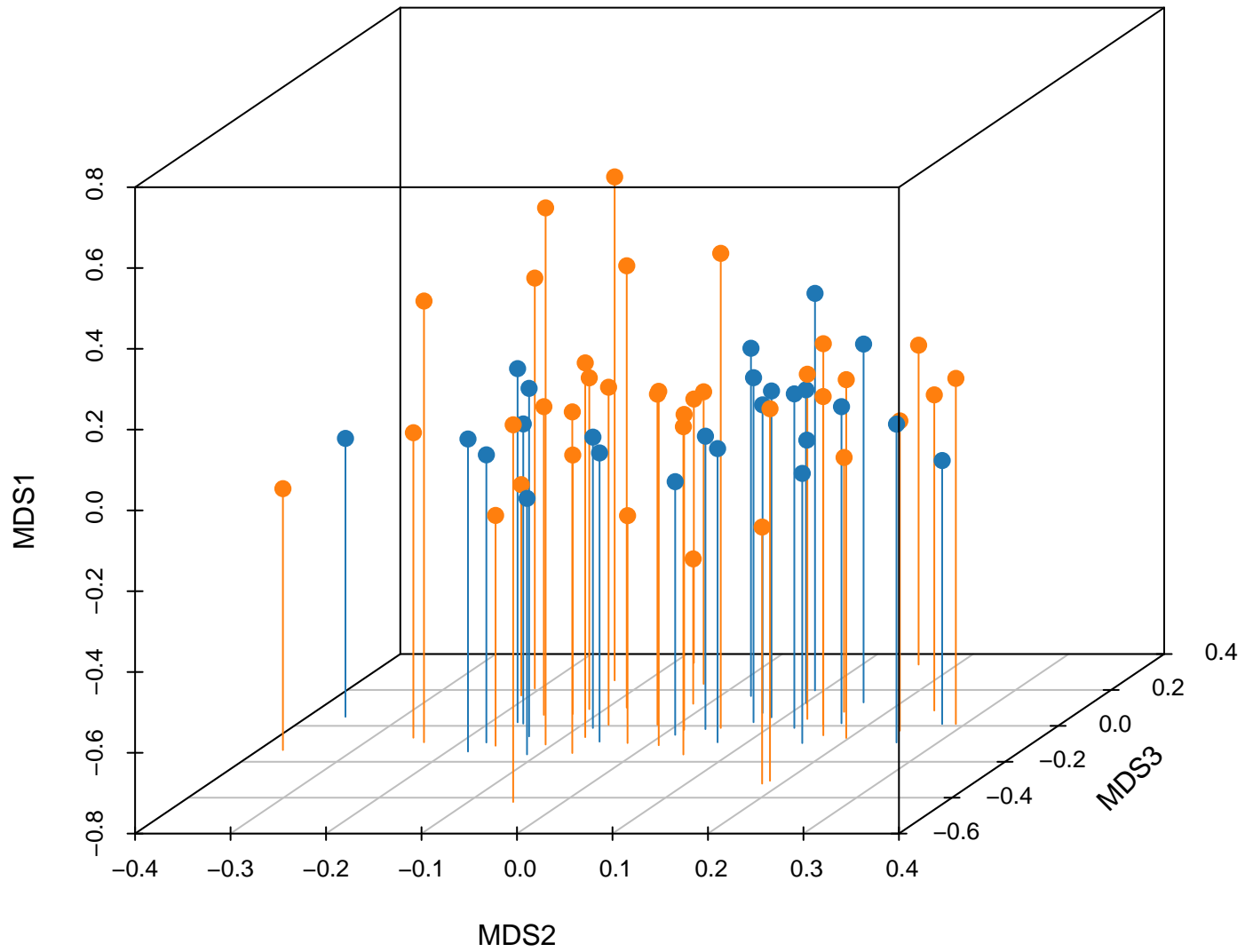

● C ● P

Supplement: Supplementary file 1 [file DataSheet1.zip › compare_1/BetaDiversity/NMDS/All_group/jaccard.3D.NMDS.pdf]

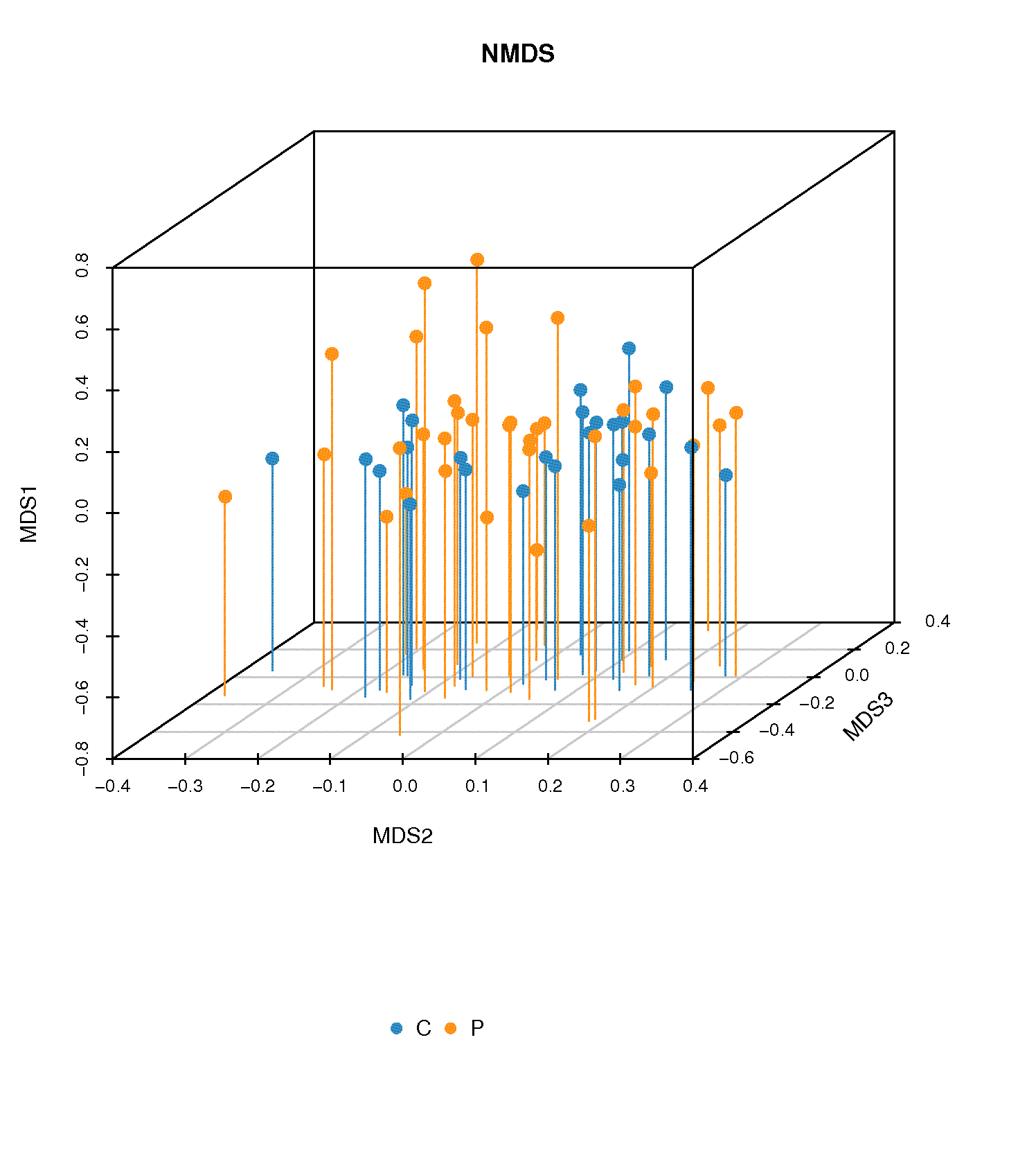

Supplement: Supplementary file 1 [file DataSheet1.zip › compare_1/BetaDiversity/NMDS/All_group/jaccard.3D.NMDS.pdf.png]

# NMDS

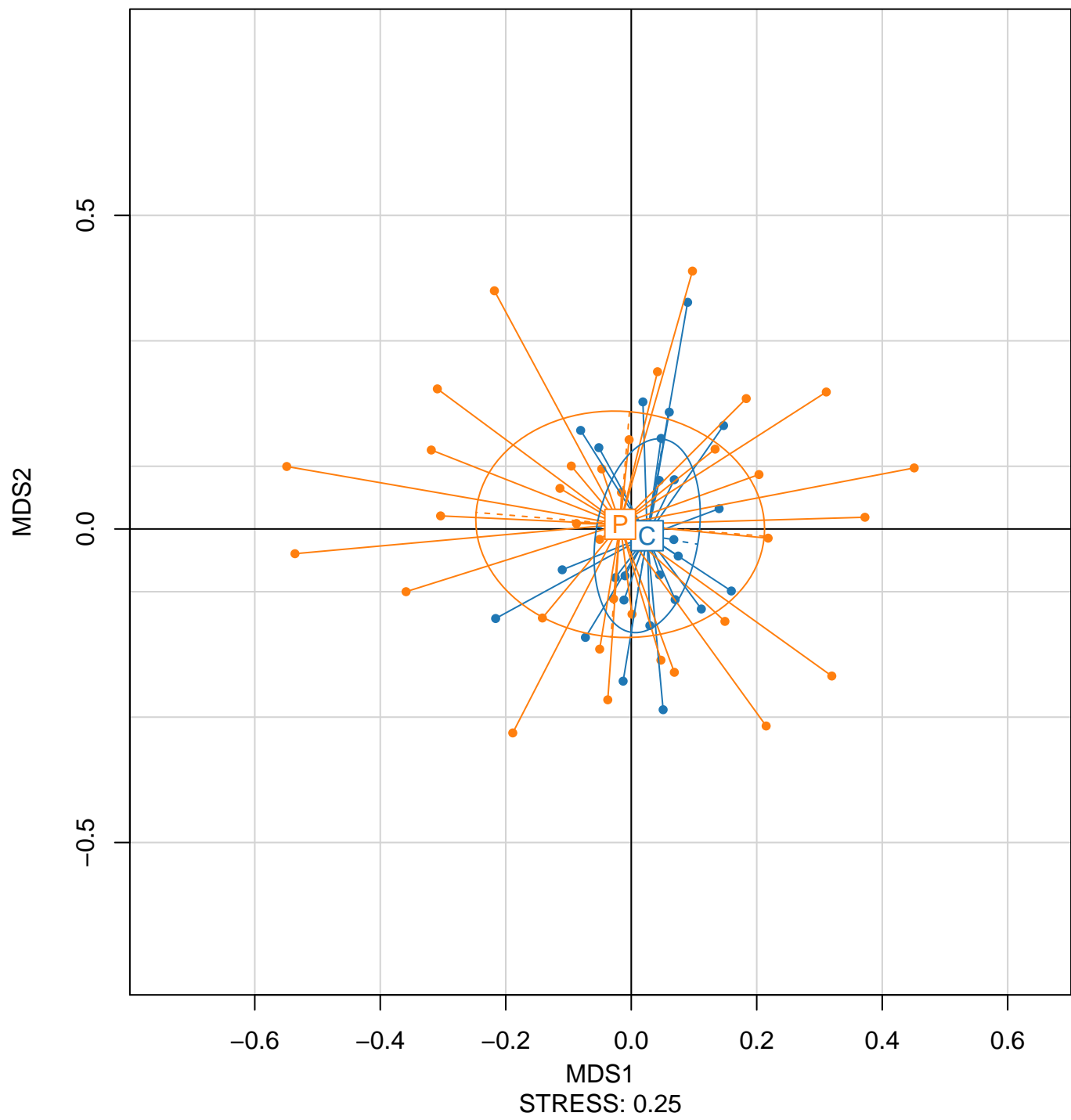

Supplement: Supplementary file 1 [file DataSheet1.zip › compare_1/BetaDiversity/NMDS/All_group/jaccard.NMDS.pdf]

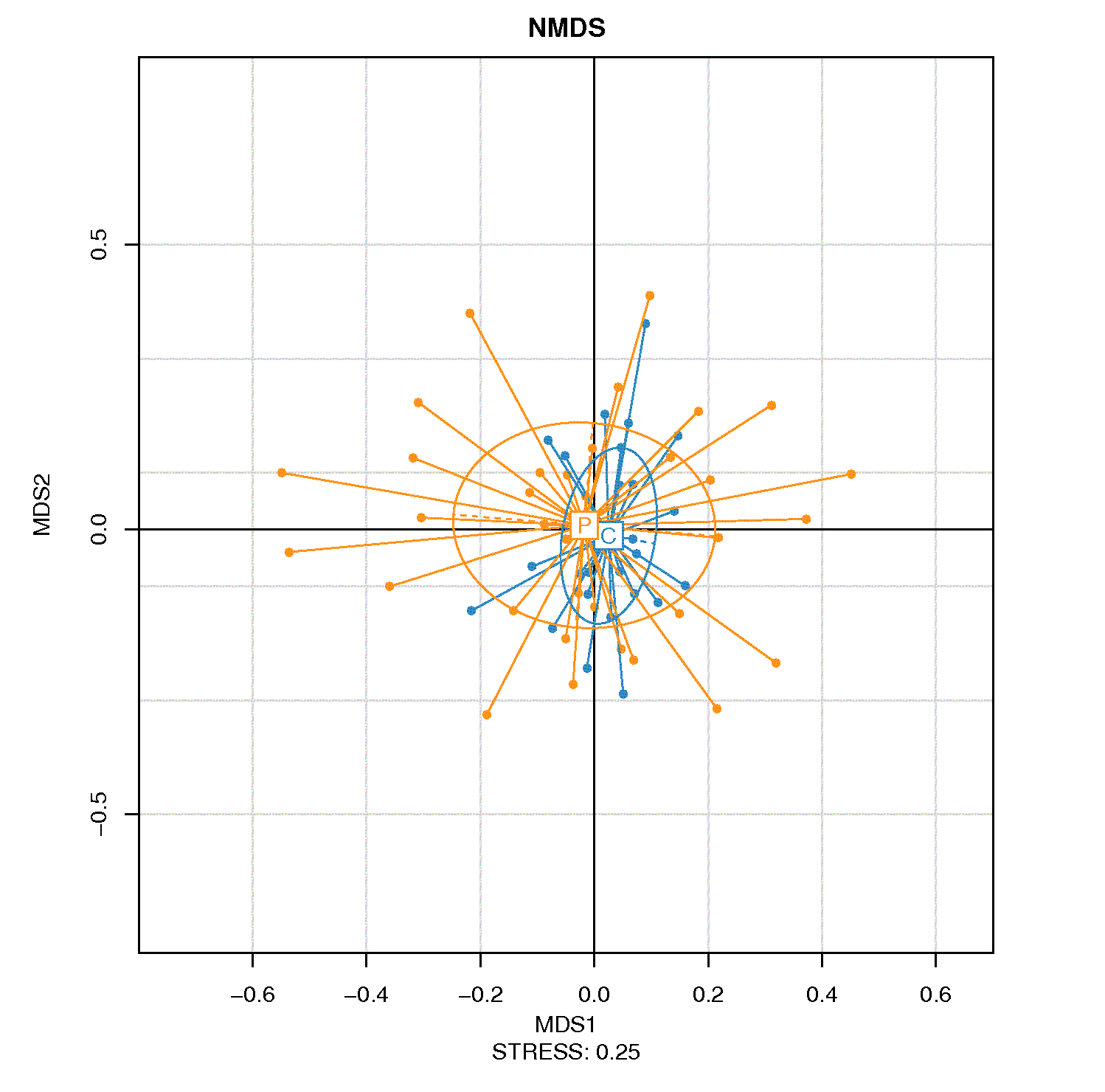

Supplement: Supplementary file 1 [file DataSheet1.zip › compare_1/BetaDiversity/NMDS/All_group/jaccard.NMDS.pdf.png]

# PCA

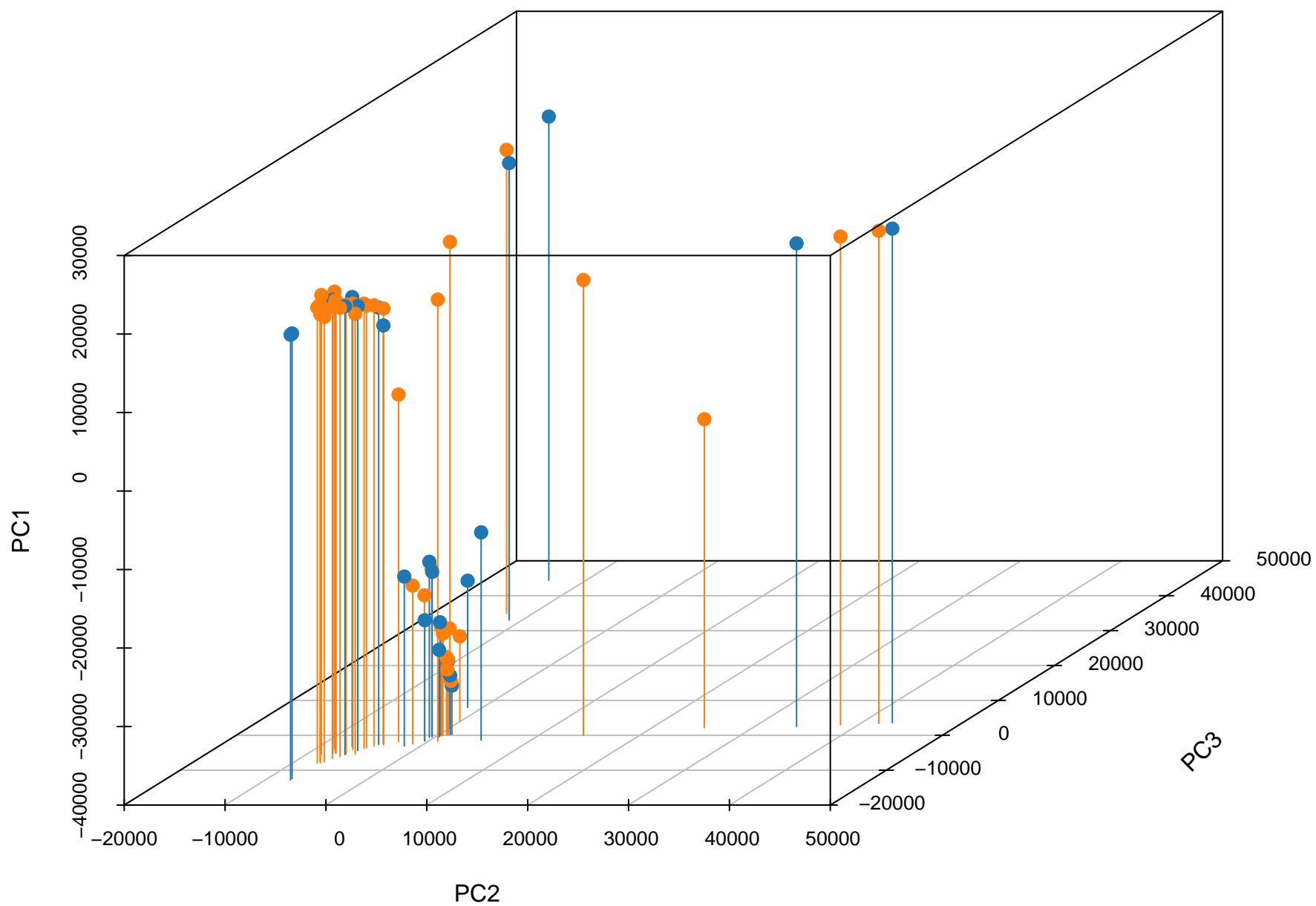

● C ● P

Supplement: Supplementary file 1 [file DataSheet1.zip › compare_1/BetaDiversity/PCA/All_group/3D.PCA.pdf]

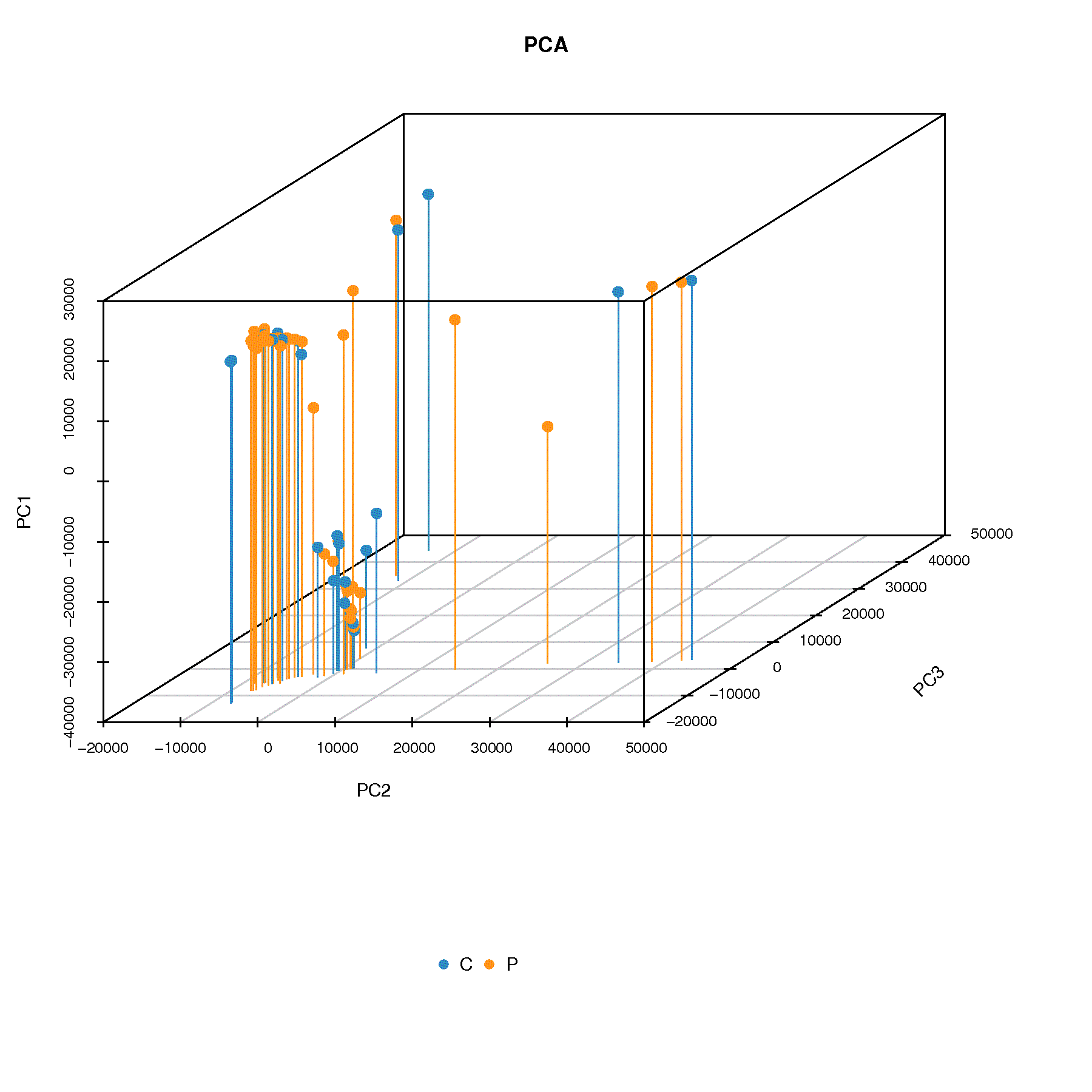

Supplement: Supplementary file 1 [file DataSheet1.zip › compare_1/BetaDiversity/PCA/All_group/3D.PCA.pdf.png]

# PCA

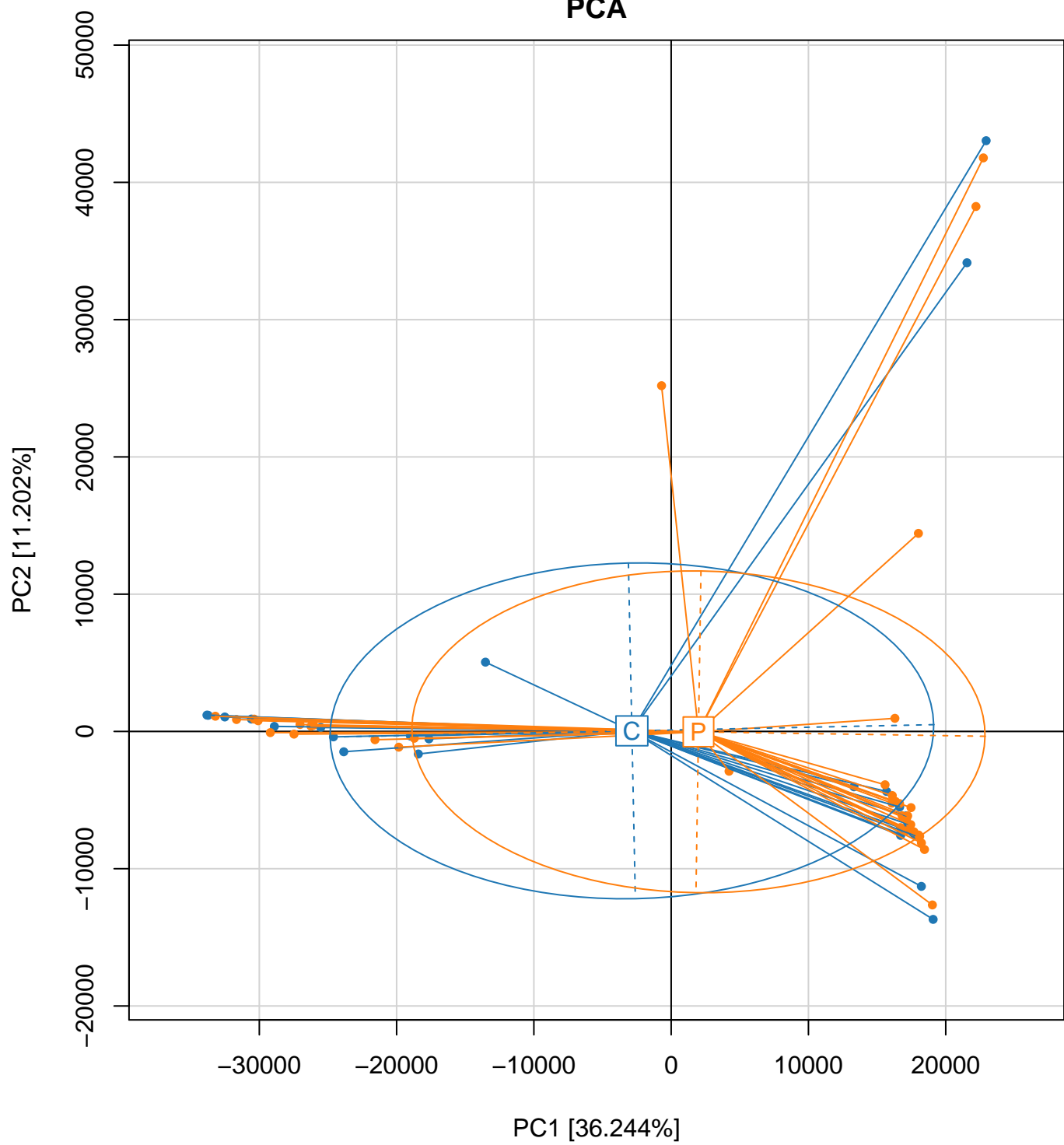

Supplement: Supplementary file 1 [file DataSheet1.zip › compare_1/BetaDiversity/PCA/All_group/PCA.pdf]

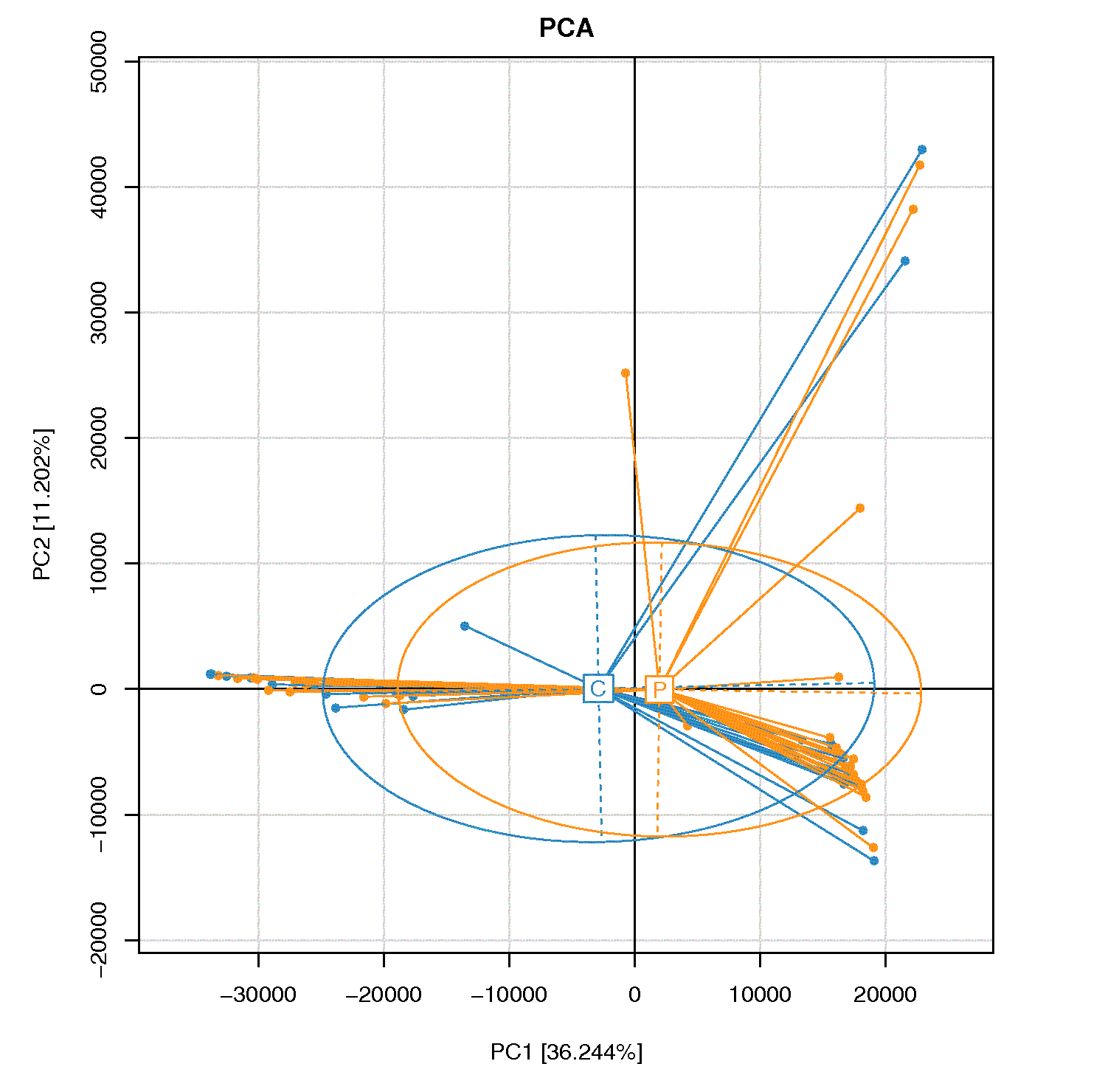

Supplement: Supplementary file 1 [file DataSheet1.zip › compare_1/BetaDiversity/PCA/All_group/PCA.pdf.png]

# PCoA

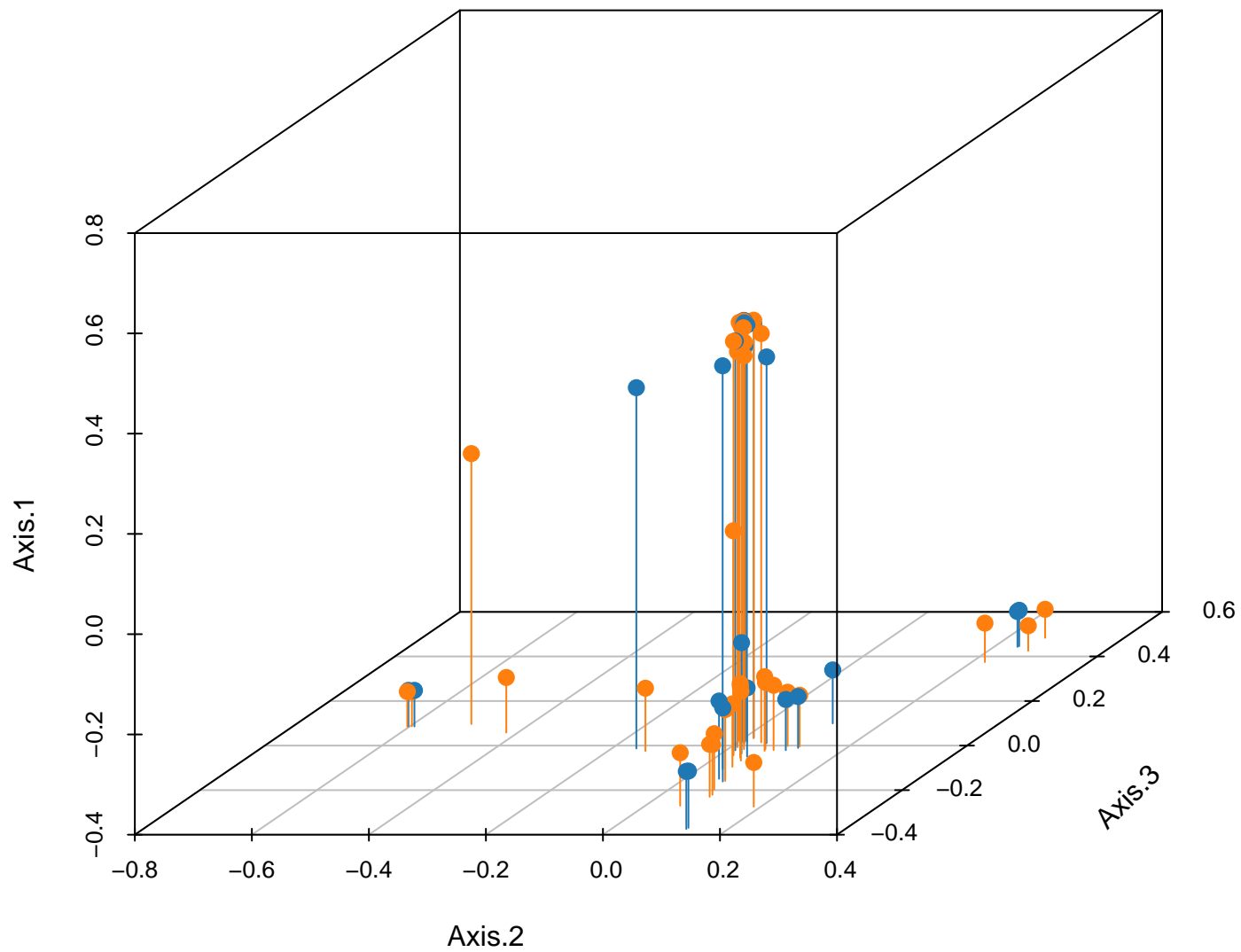

Supplement: Supplementary file 1 [file DataSheet1.zip › compare_1/BetaDiversity/PCoA/All_group/bray.3D.PCoA.pdf]

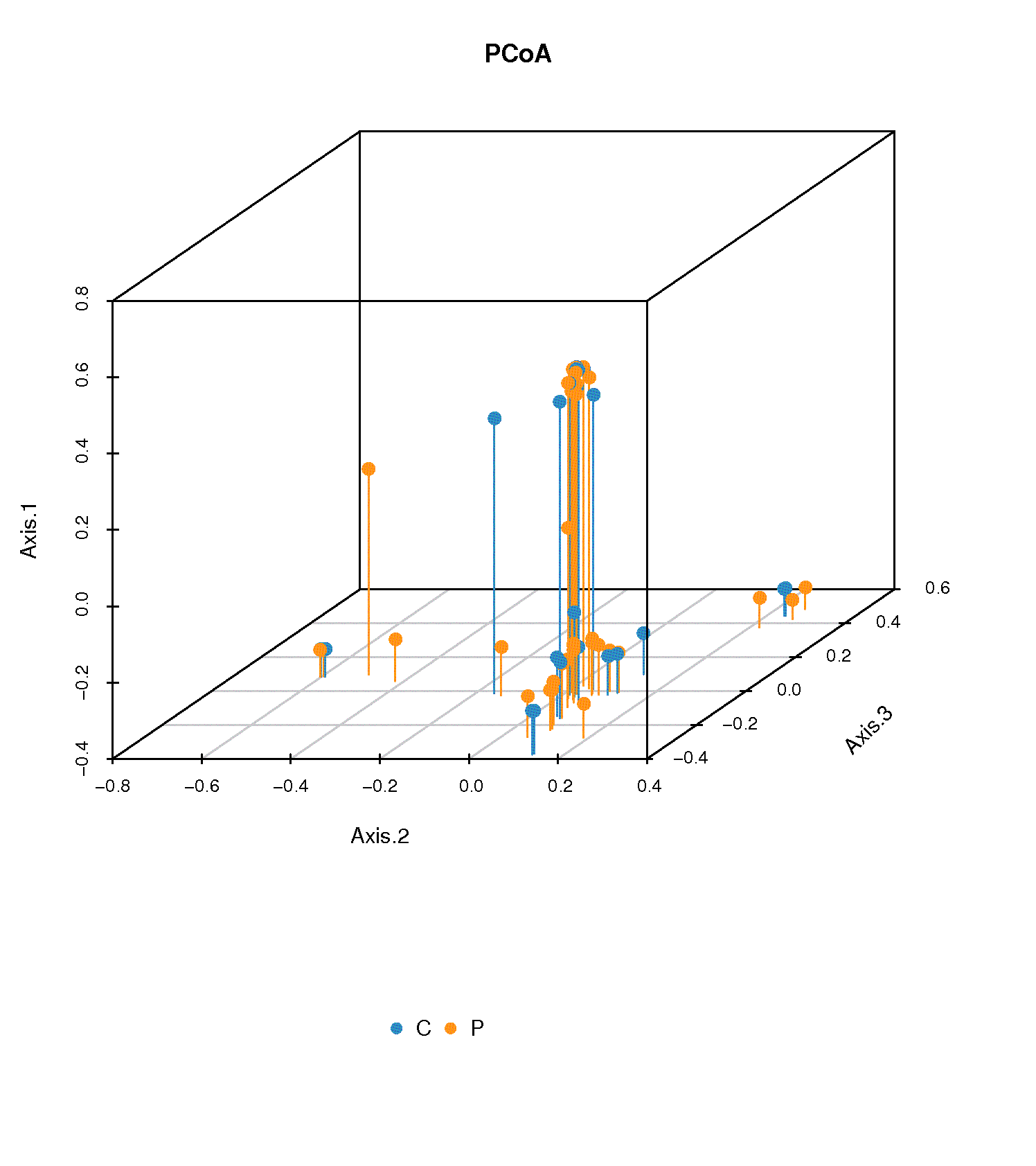

Supplement: Supplementary file 1 [file DataSheet1.zip › compare_1/BetaDiversity/PCoA/All_group/bray.3D.PCoA.pdf.png]

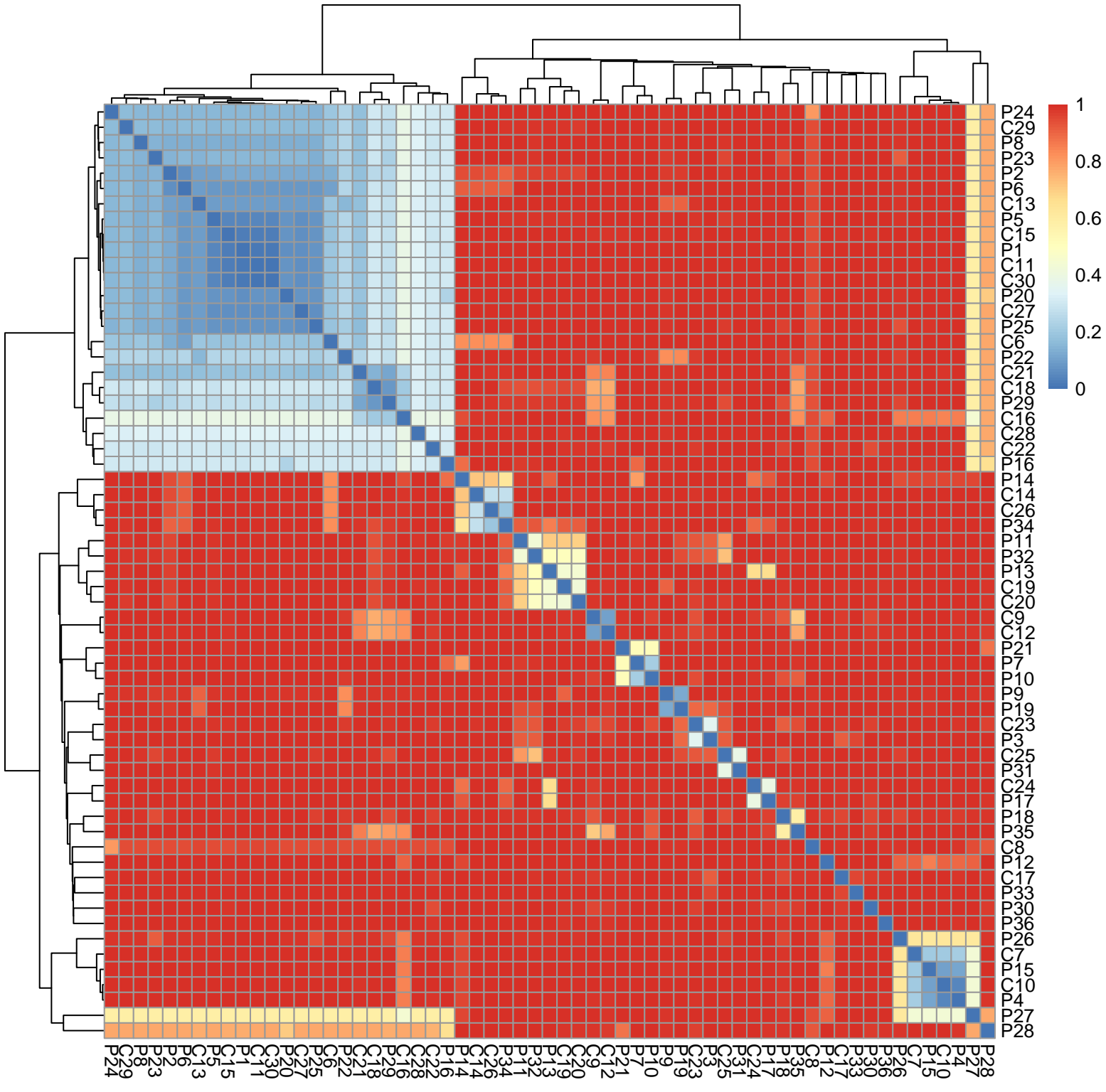

Supplement: Supplementary file 1 [file DataSheet1.zip › compare_1/BetaDiversity/PCoA/All_group/bray.dist.Heatmap.pdf]

# PCoA

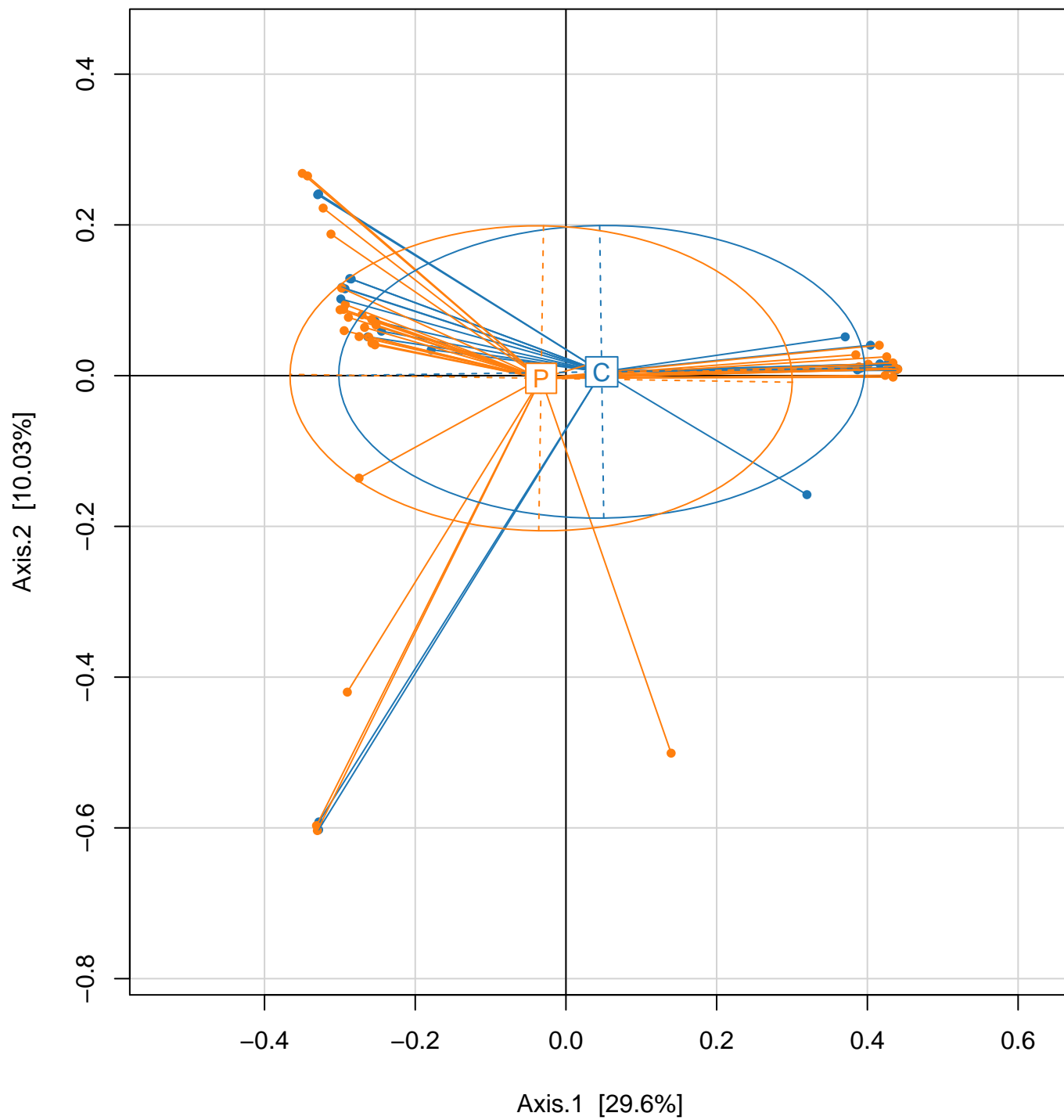

Supplement: Supplementary file 1 [file DataSheet1.zip › compare_1/BetaDiversity/PCoA/All_group/bray.PCoA.pdf]

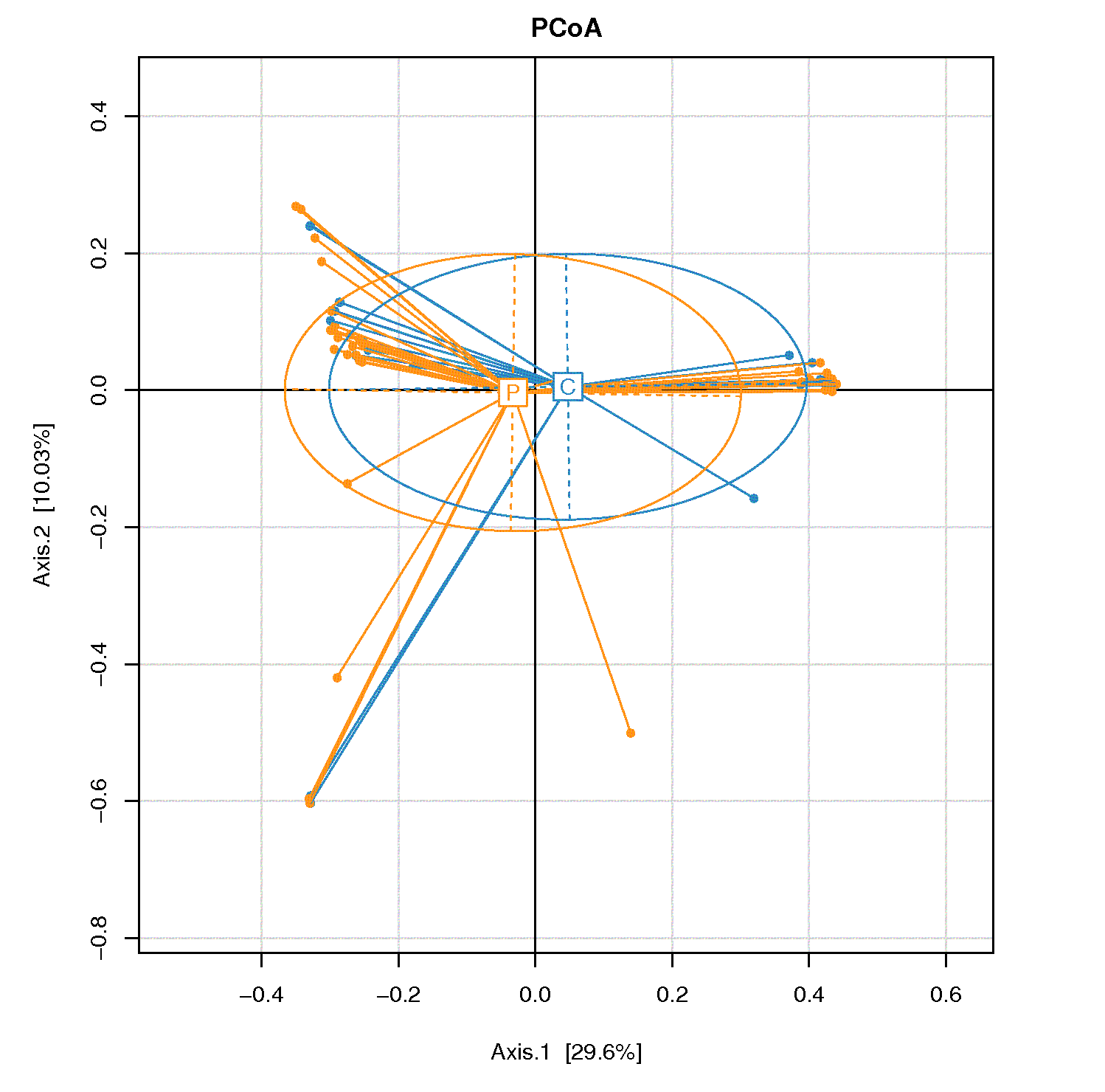

Supplement: Supplementary file 1 [file DataSheet1.zip › compare_1/BetaDiversity/PCoA/All_group/bray.PCoA.pdf.png]

# PCoA

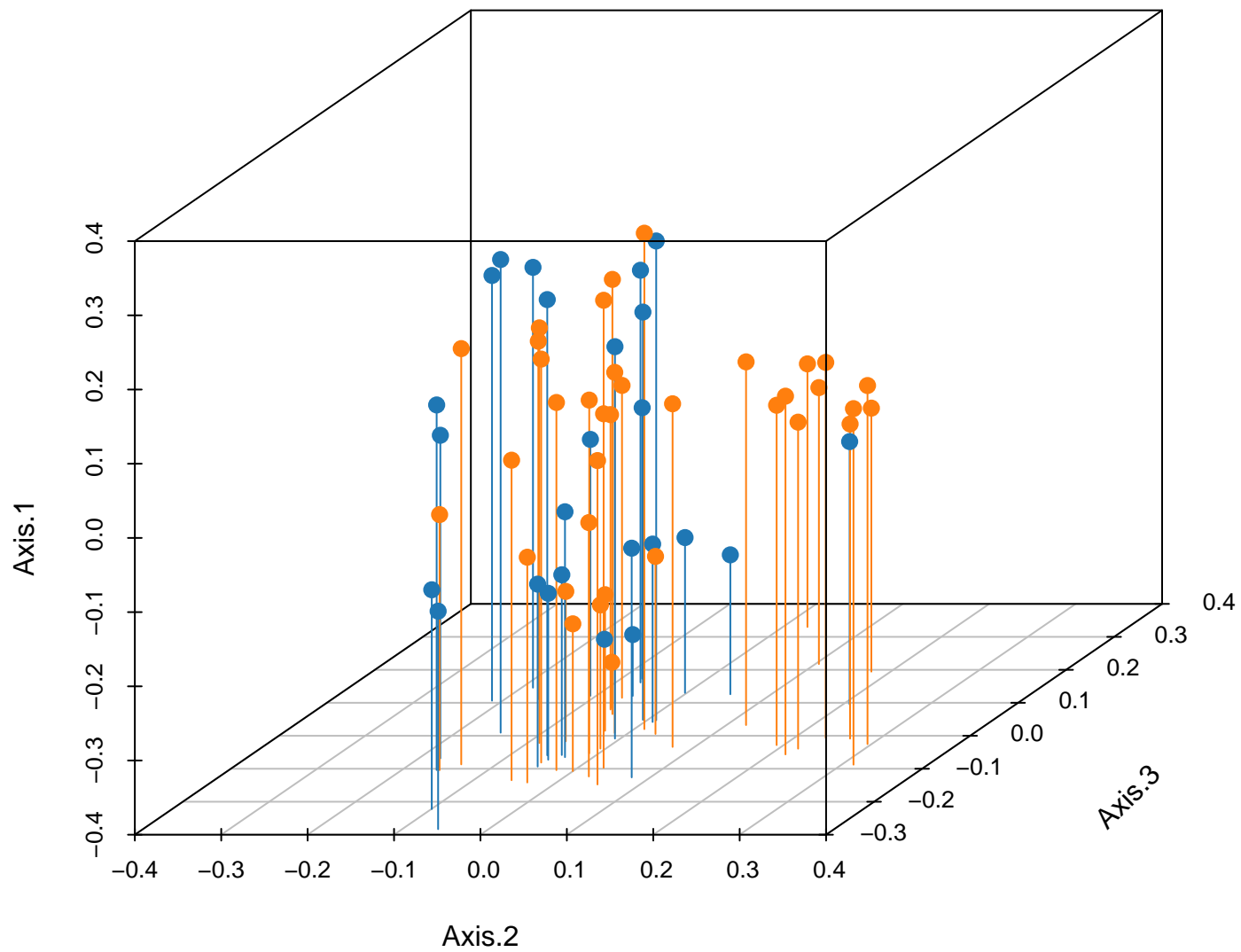

● C ● P

Supplement: Supplementary file 1 [file DataSheet1.zip › compare_1/BetaDiversity/PCoA/All_group/jaccard.3D.PCoA.pdf]

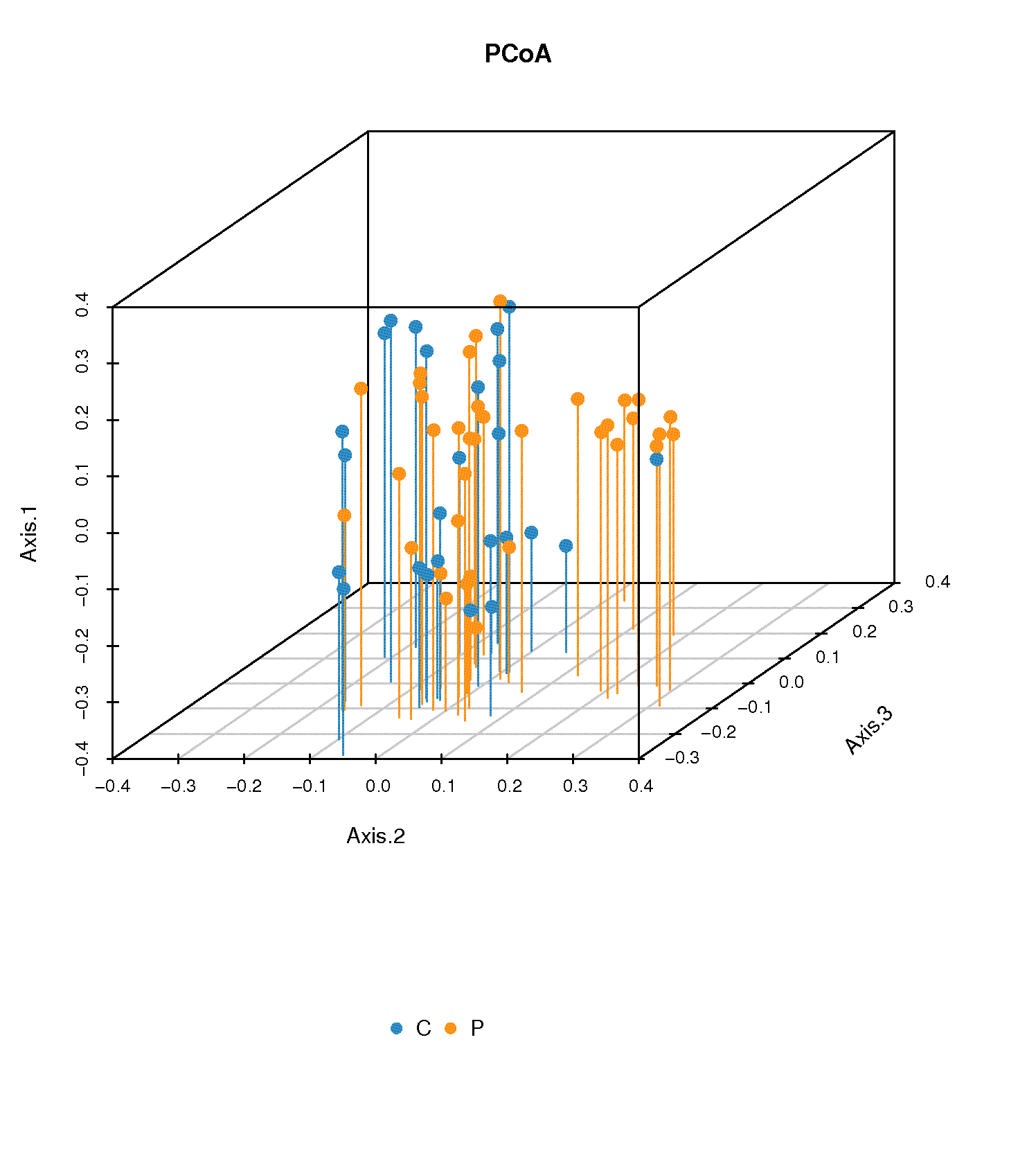

Supplement: Supplementary file 1 [file DataSheet1.zip › compare_1/BetaDiversity/PCoA/All_group/jaccard.3D.PCoA.pdf.png]

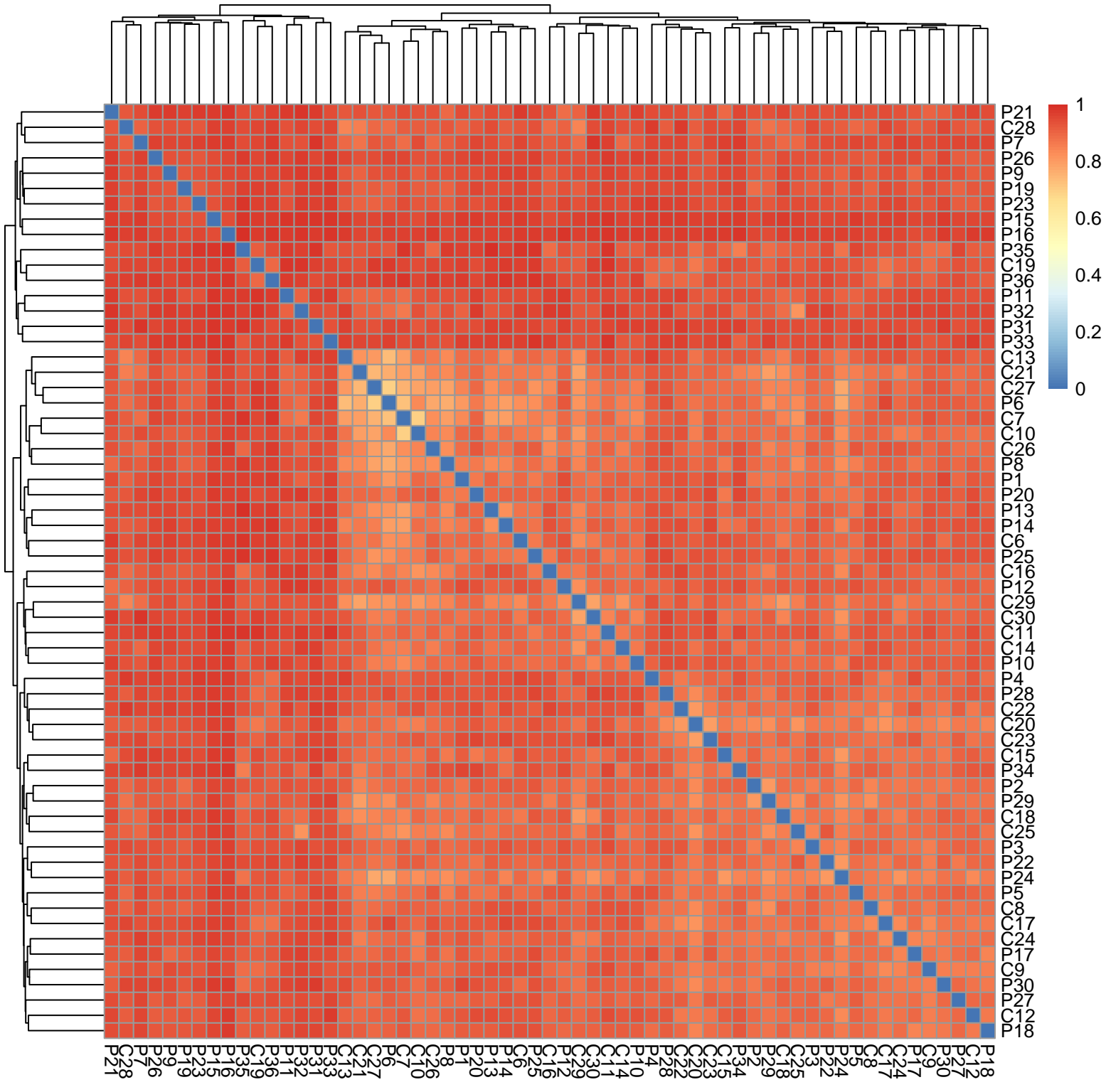

Supplement: Supplementary file 1 [file DataSheet1.zip › compare_1/BetaDiversity/PCoA/All_group/jaccard.dist.Heatmap.pdf]

# PCoA

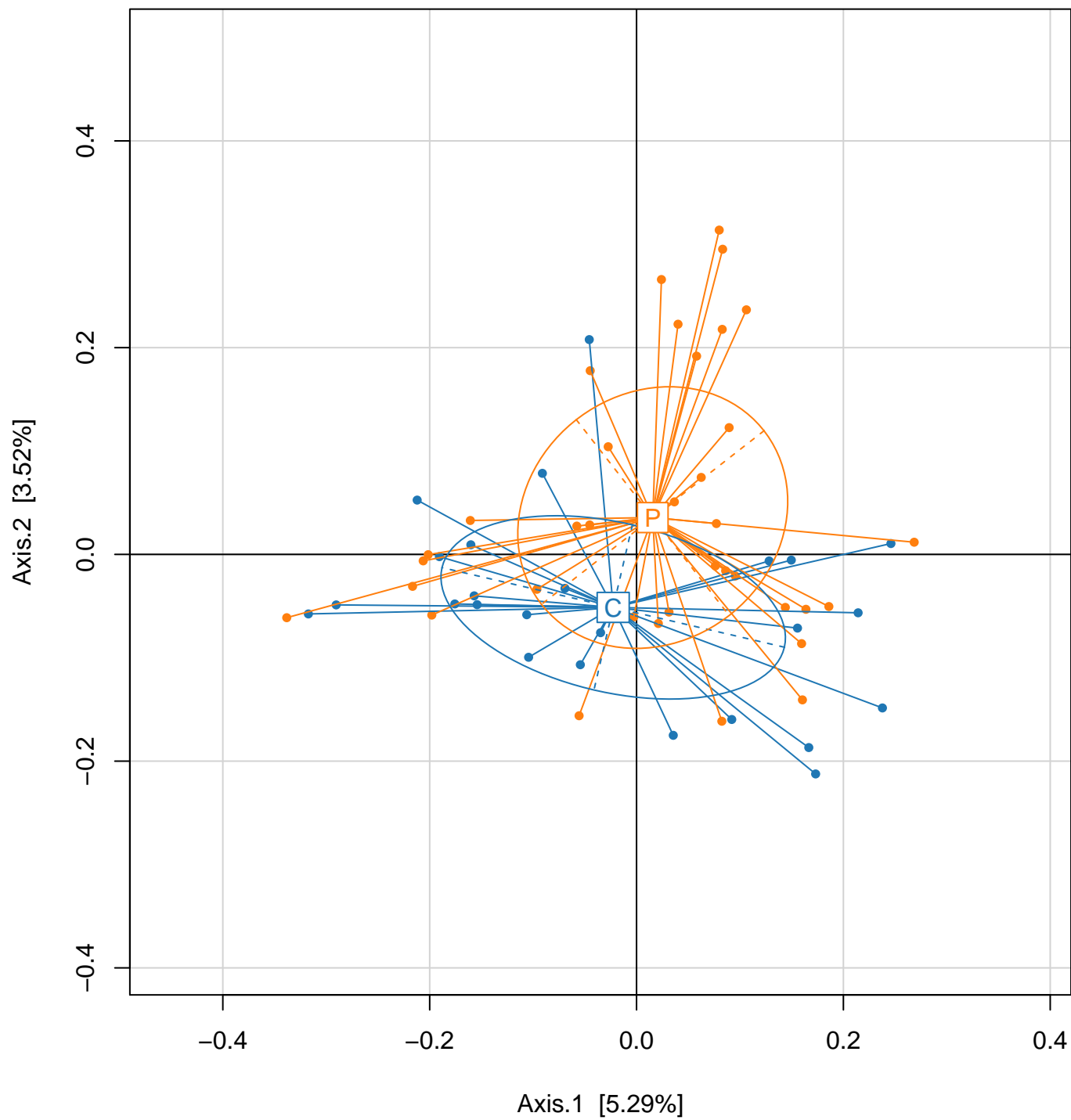

Supplement: Supplementary file 1 [file DataSheet1.zip › compare_1/BetaDiversity/PCoA/All_group/jaccard.PCoA.pdf]

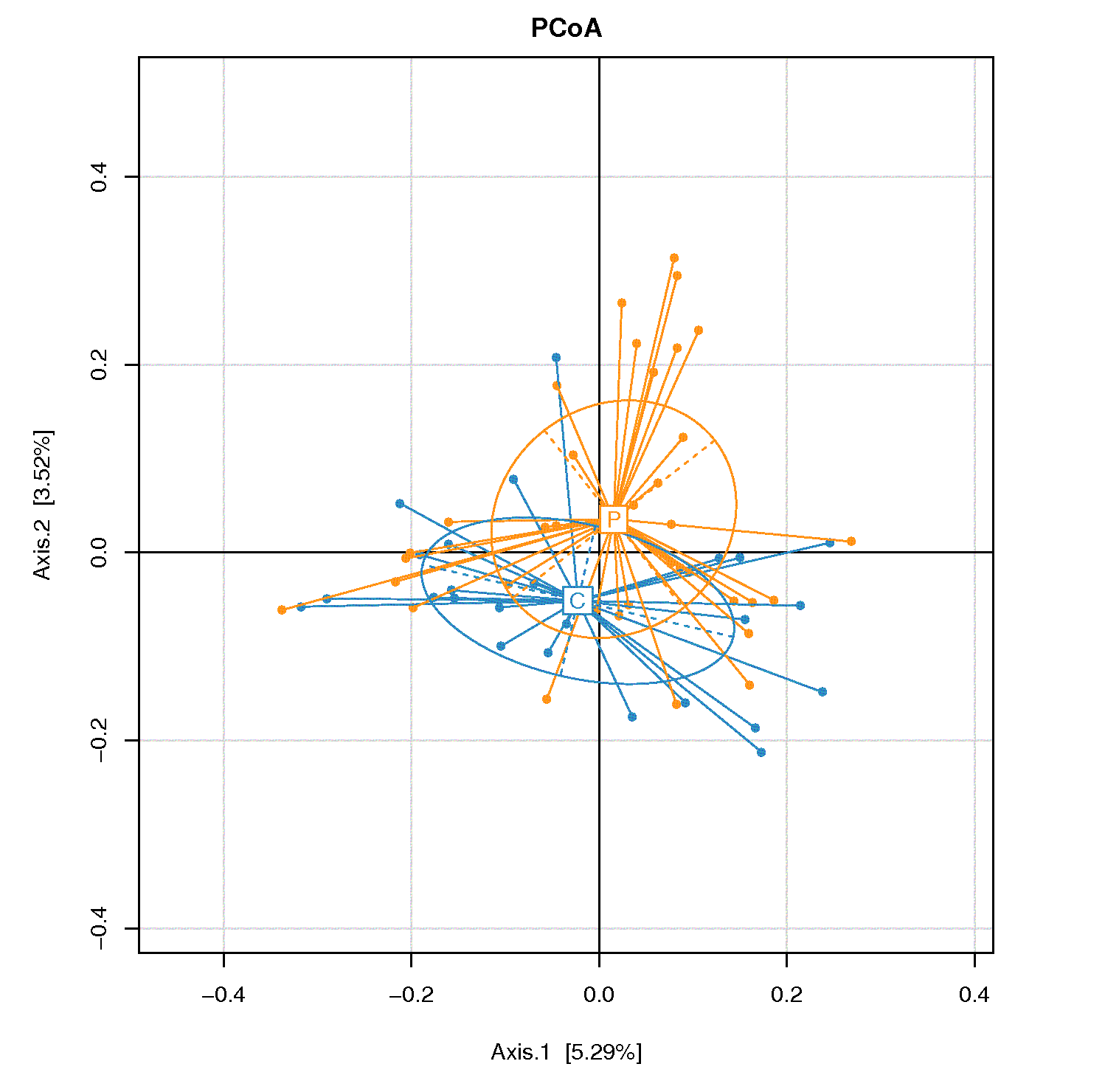

Supplement: Supplementary file 1 [file DataSheet1.zip › compare_1/BetaDiversity/PCoA/All_group/jaccard.PCoA.pdf.png]

# PLS\_DA

X-variate 2: 6.26% expl.var

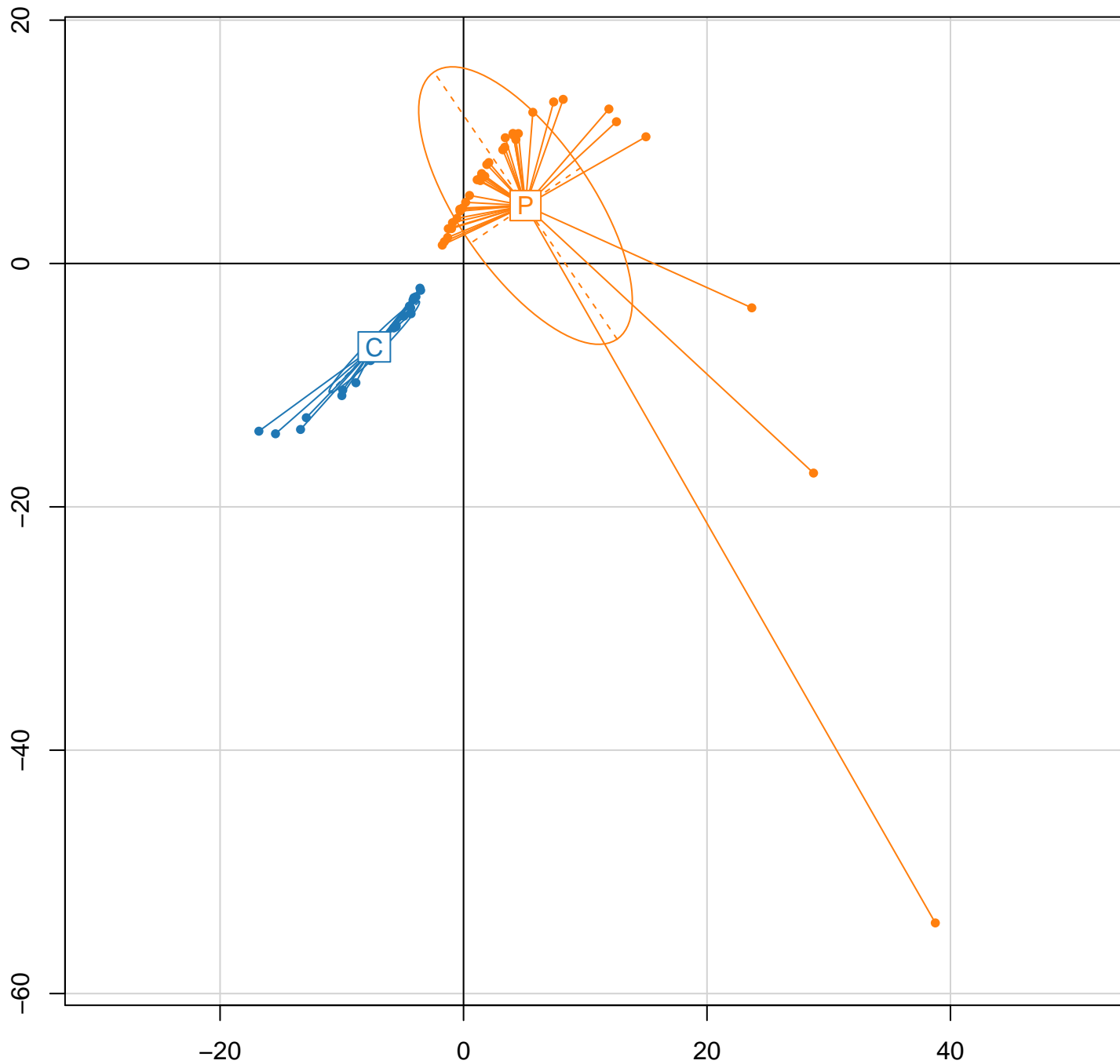

X-variate 1: 6.68% expl.var

Supplement: Supplementary file 1 [file DataSheet1.zip › compare_1/BetaDiversity/PLS_DA/All_group/PLS-DA.pdf]

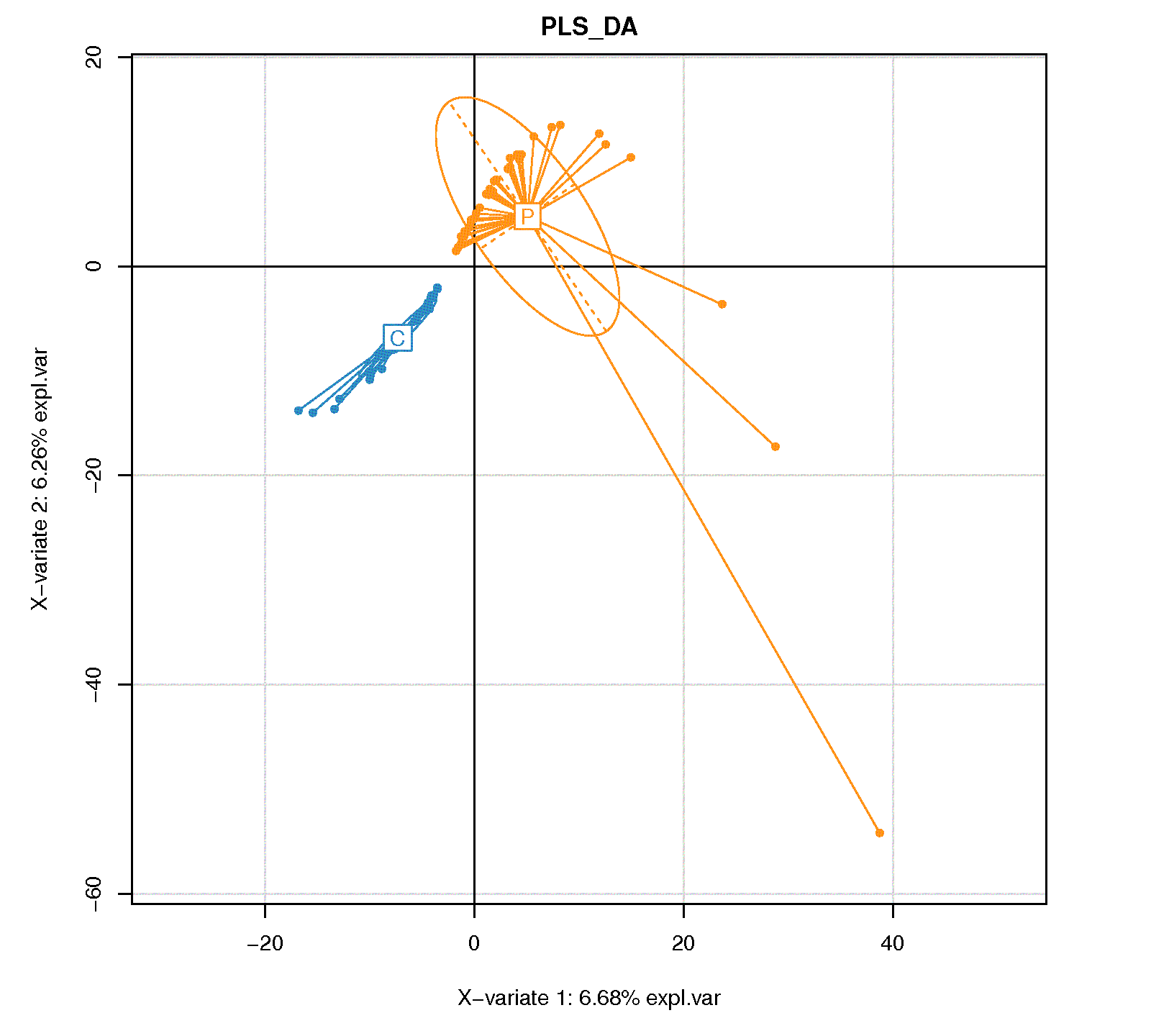

Supplement: Supplementary file 1 [file DataSheet1.zip › compare_1/BetaDiversity/PLS_DA/All_group/PLS-DA.pdf.png]

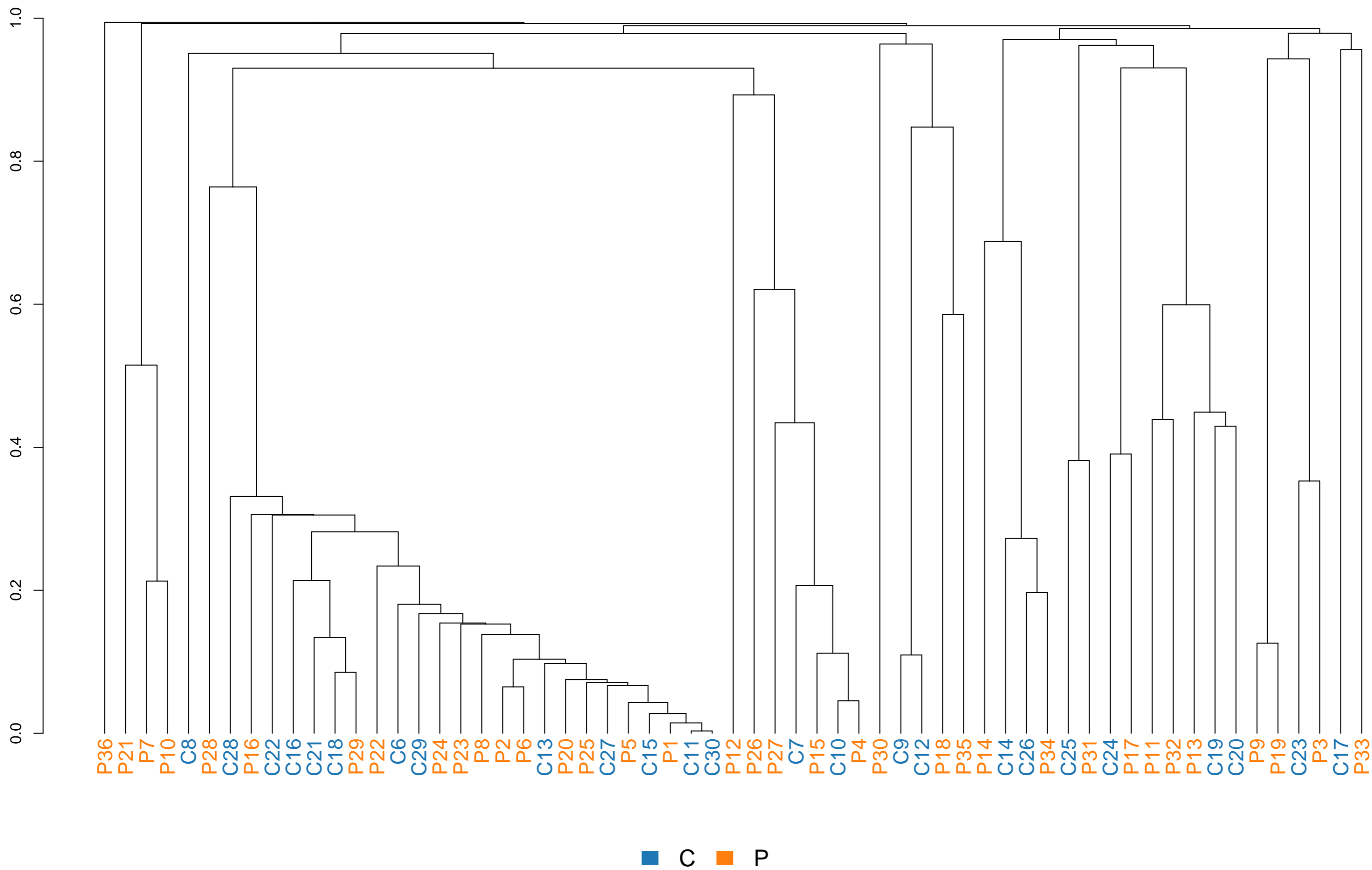

Supplement: Supplementary file 1 [file DataSheet1.zip › compare_1/BetaDiversity/SamplesTree/SamplesClusterTree.pdf]

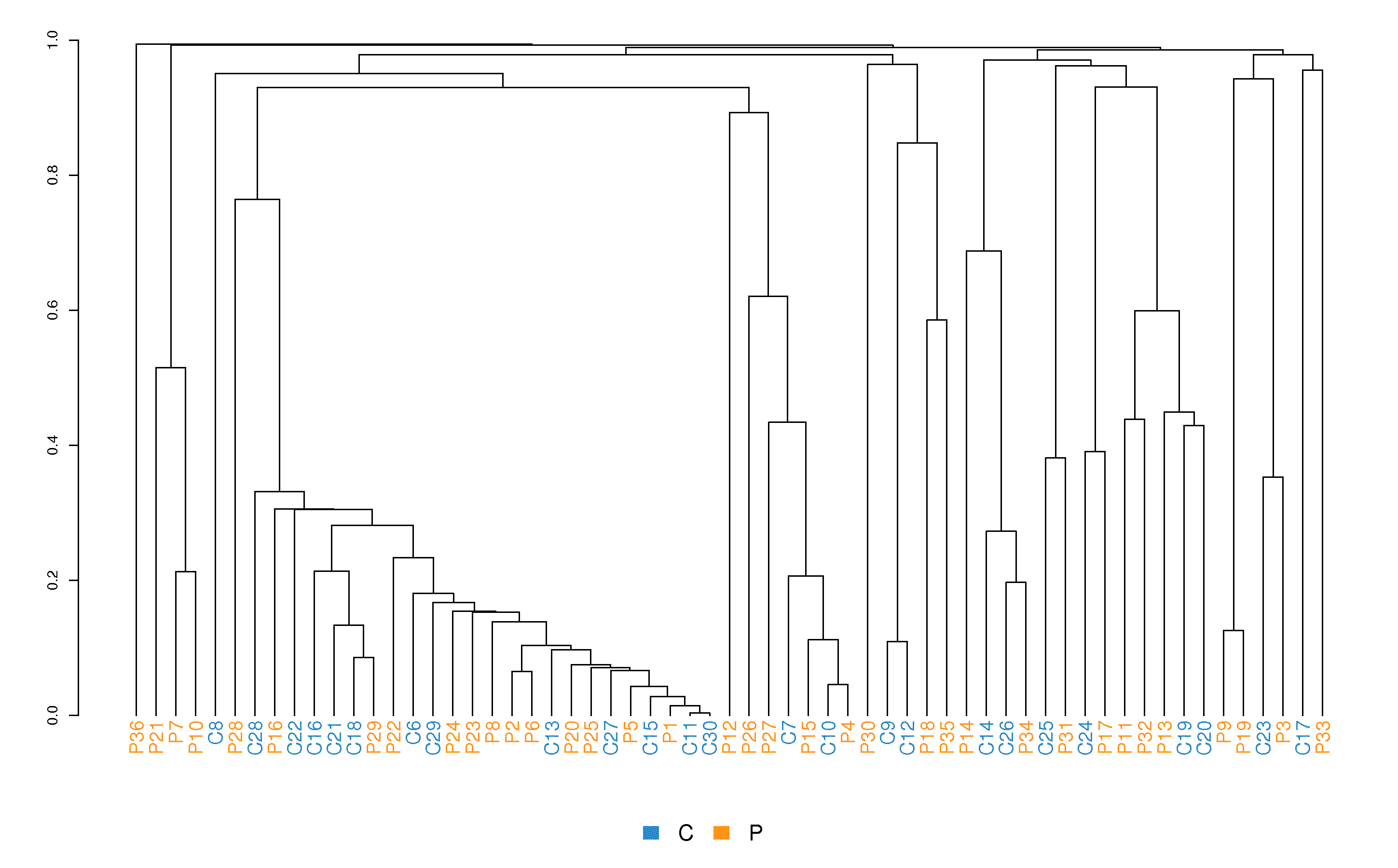

Supplement: Supplementary file 1 [file DataSheet1.zip › compare_1/BetaDiversity/SamplesTree/SamplesClusterTree.pdf.png]

Relative abundance (%)

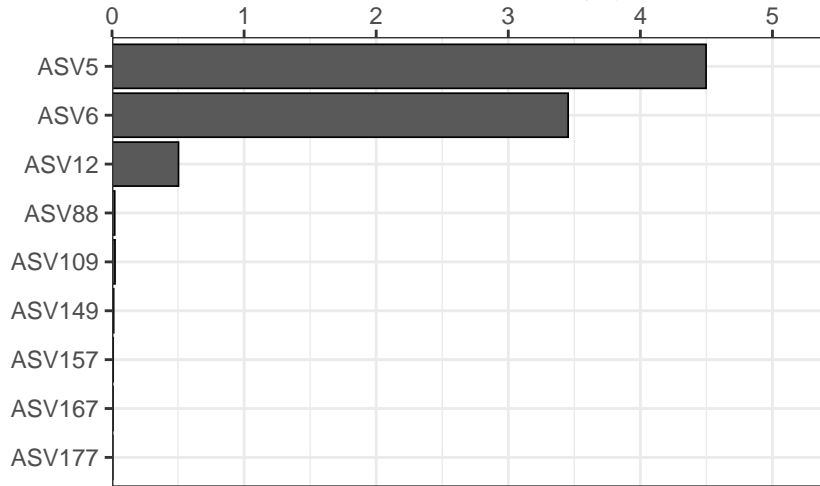

Indicator.values · 0.0 ● 0.2 ● 0.4 ● 0.6

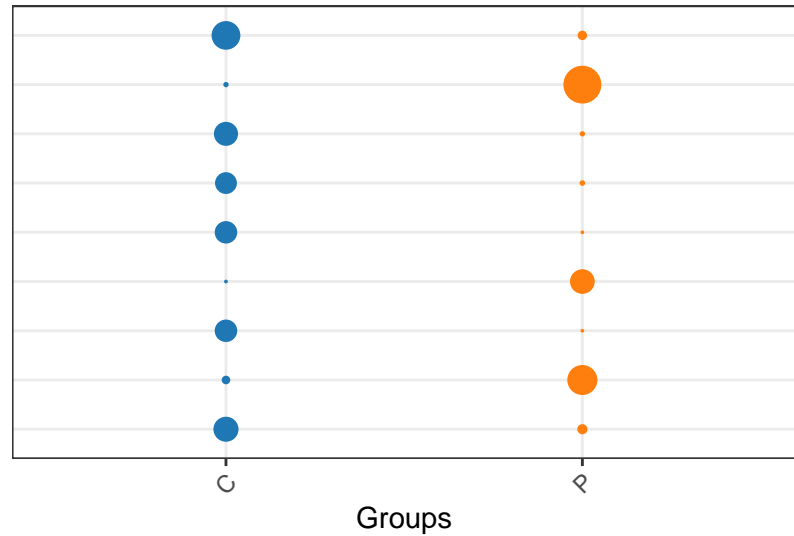

Supplement: Supplementary file 1 [file DataSheet1.zip › compare_1/Community/Indicator/indicators.pdf]

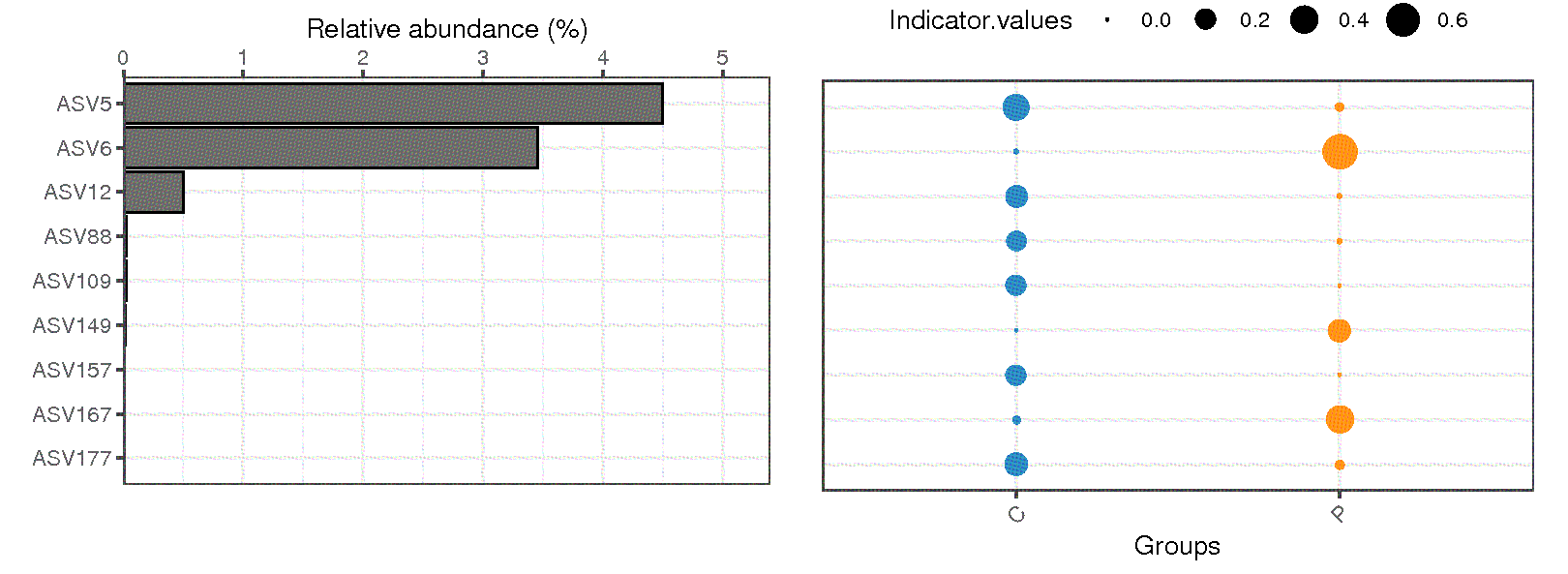

Supplement: Supplementary file 1 [file DataSheet1.zip › compare_1/Community/Indicator/indicators.pdf.png]

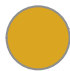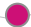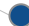

● Bacilli

● Unclassified

● Unassigned

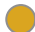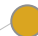

Supplement: Supplementary file 1 [file DataSheet1.zip › compare_1/Community/Network/network.with.class.classif.pdf]

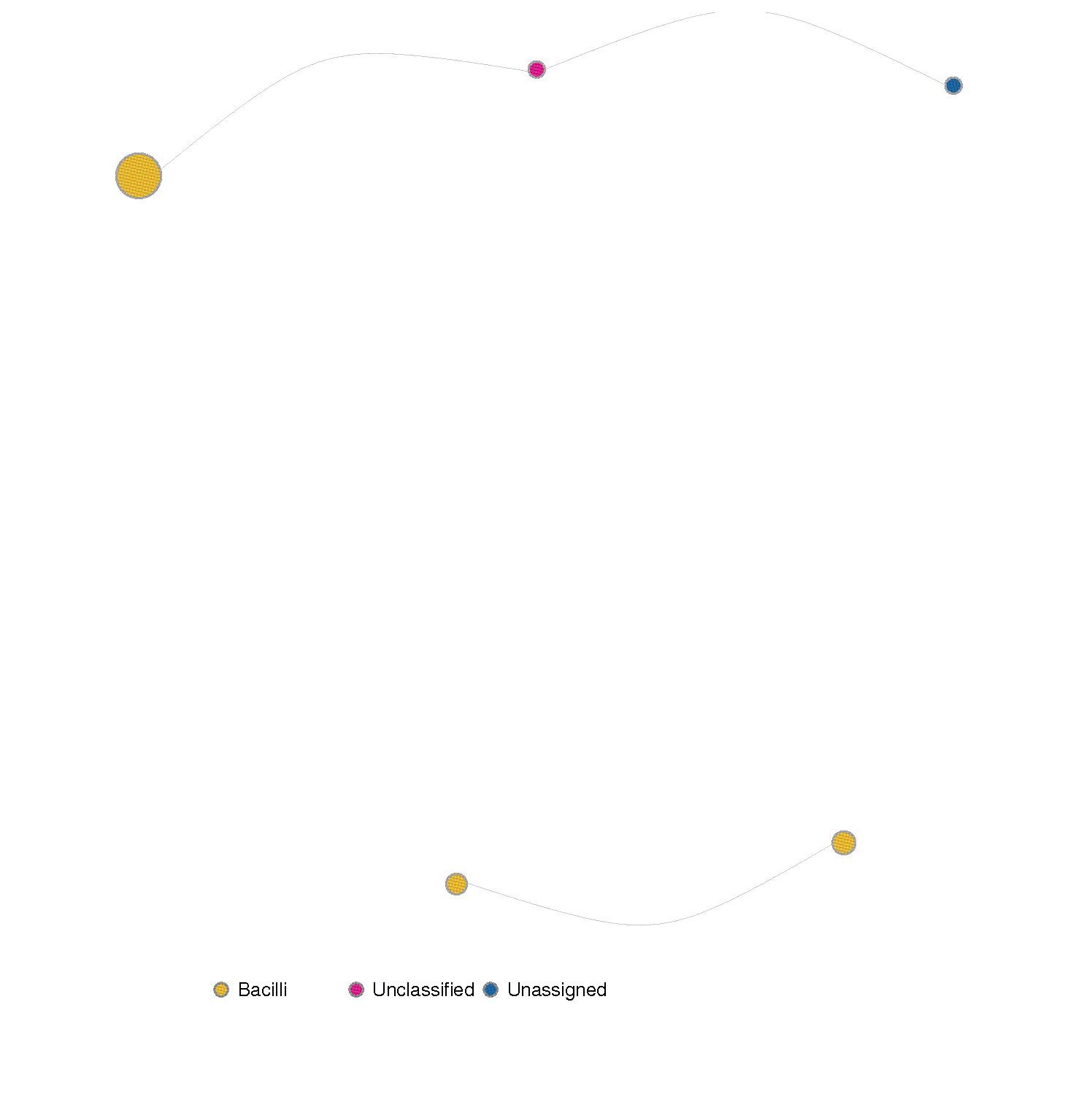

Supplement: Supplementary file 1 [file DataSheet1.zip › compare_1/Community/Network/network.with.class.classif.pdf.png]

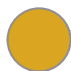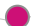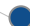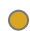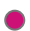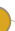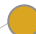

● Lactobacillaceae    ● Unclassified    ● Unassigned

Supplement: Supplementary file 1 [file DataSheet1.zip › compare_1/Community/Network/network.with.family.classif.pdf]

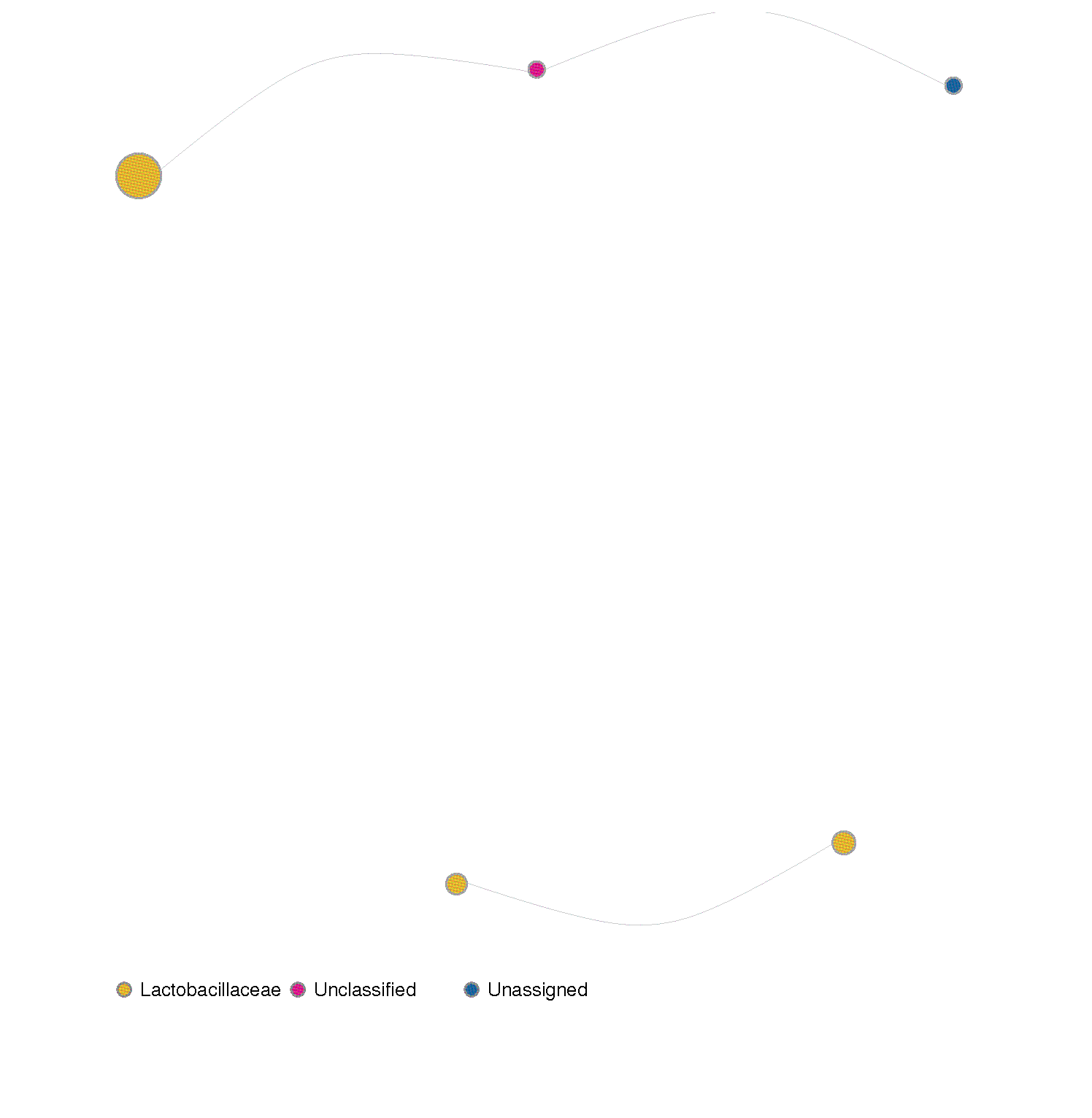

Supplement: Supplementary file 1 [file DataSheet1.zip › compare_1/Community/Network/network.with.family.classif.pdf.png]

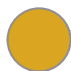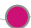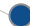

● Lactobacillus ● Unclassified ● Unassigned

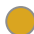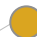

Supplement: Supplementary file 1 [file DataSheet1.zip › compare_1/Community/Network/network.with.genus.classif.pdf]

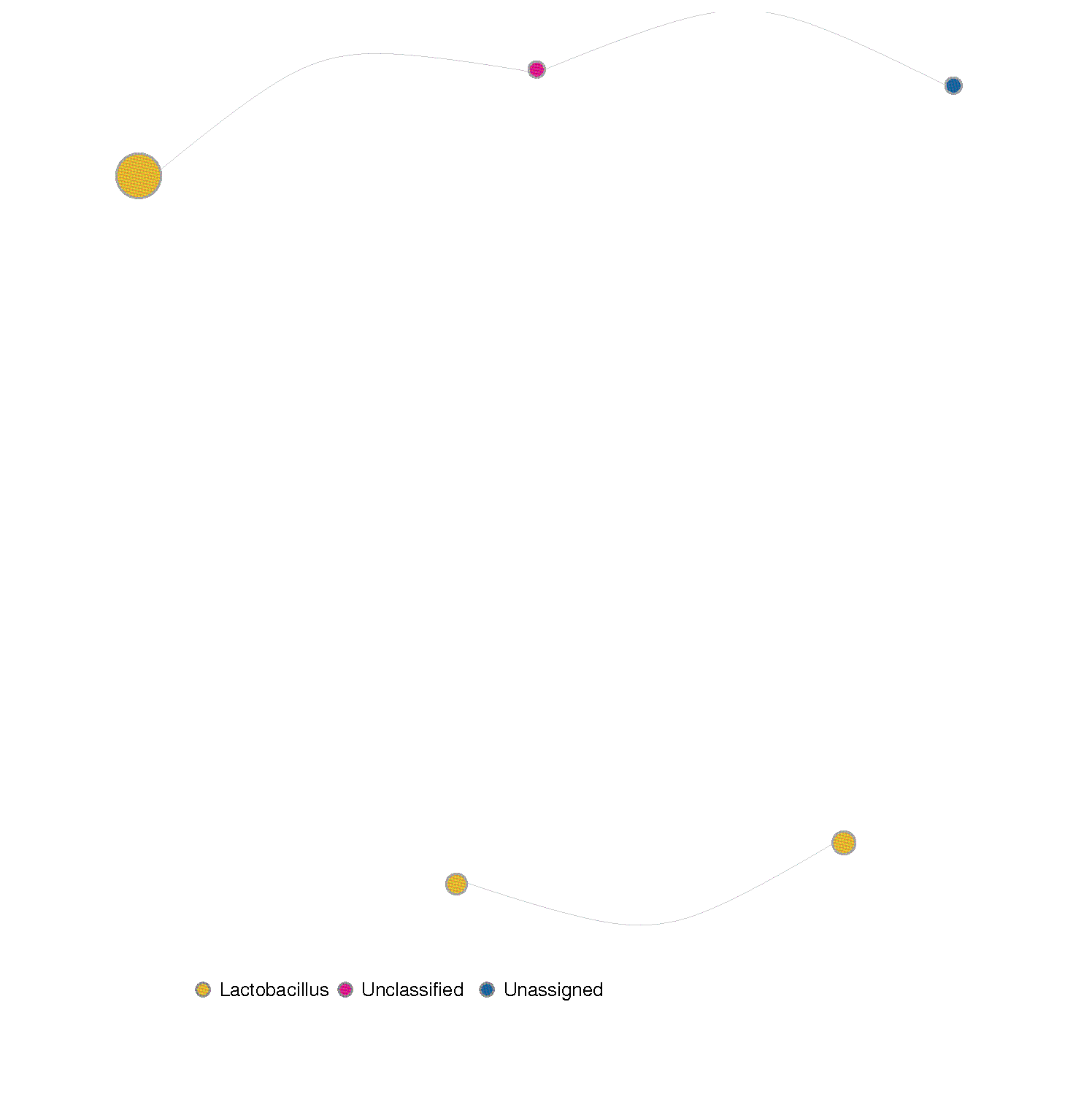

Supplement: Supplementary file 1 [file DataSheet1.zip › compare_1/Community/Network/network.with.genus.classif.pdf.png]

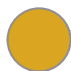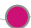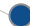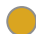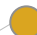

● Lactobacillales ● Unclassified ● Unassigned

Supplement: Supplementary file 1 [file DataSheet1.zip › compare_1/Community/Network/network.with.order.classif.pdf]

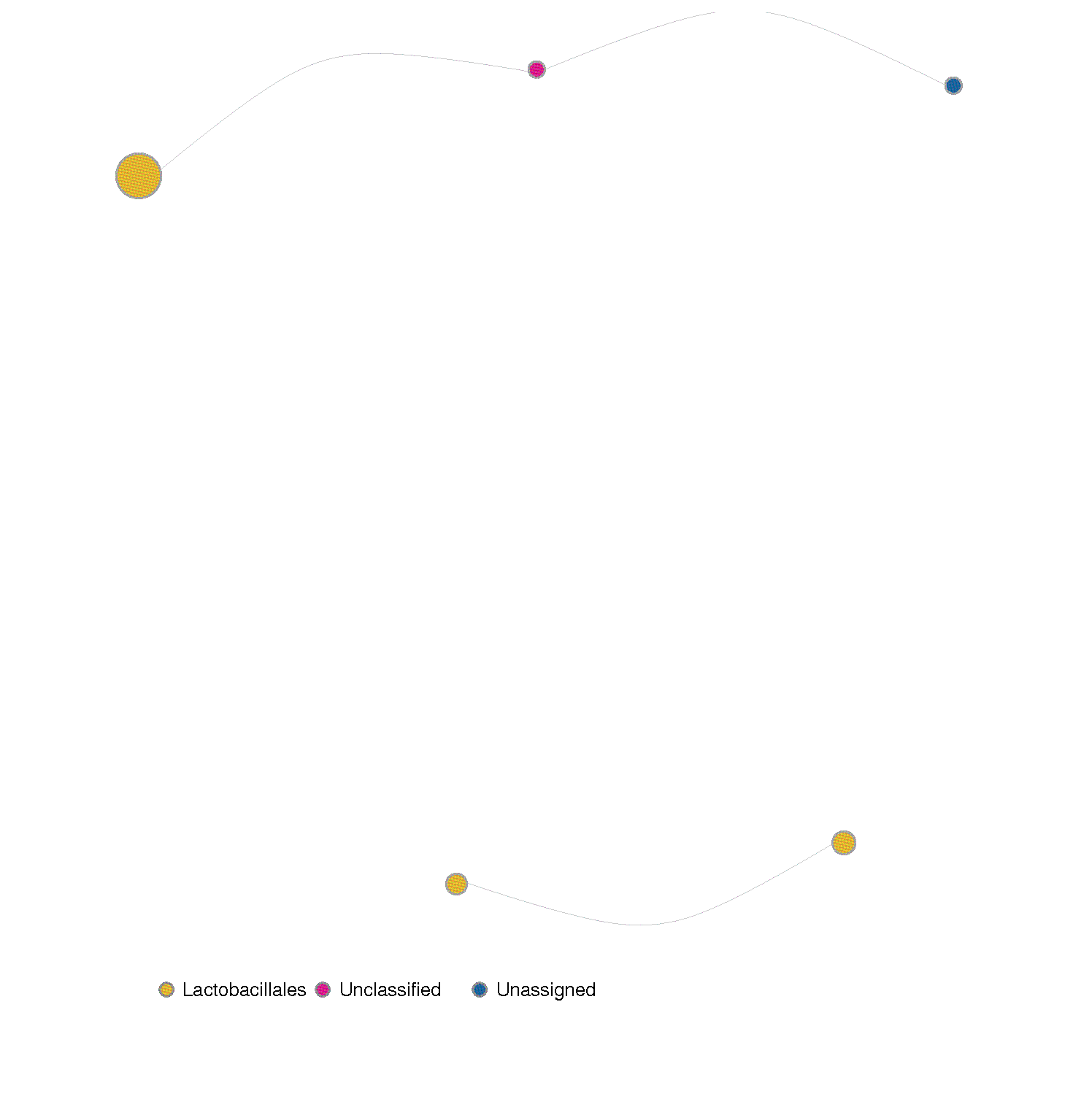

Supplement: Supplementary file 1 [file DataSheet1.zip › compare_1/Community/Network/network.with.order.classif.pdf.png]

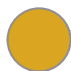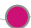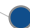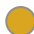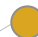

● Firmicutes ● Unclassified ● Unassigned

Supplement: Supplementary file 1 [file DataSheet1.zip › compare_1/Community/Network/network.with.phylum.classif.pdf]
